# Syntheses of Thiophene and Thiazole-Based Building Blocks and Their Utilization in the Syntheses of A-D-A Type Organic Semiconducting Materials with Dithienosilolo Central Unit

**DOI:** 10.1021/acsomega.2c02195

**Published:** 2022-07-21

**Authors:** Tomi A. O. Parviainen, Petri M. Salmela, Roosa J. Sippola, Juha P. Heiskanen

**Affiliations:** Research Unit of Sustainable Chemistry, University of Oulu, P.O. Box 4300, FI-90014 Oulu, Finland

## Abstract

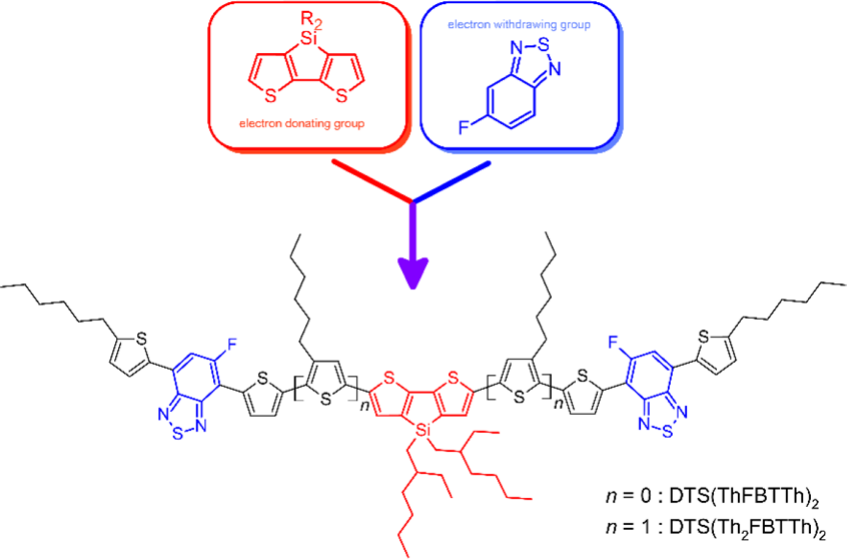

Dithienosilole moiety is an electron donating unit, and it has been applied, for example, as a part of small molecular and polymeric electron donors in high performance organic photovoltaic cells. Herein, we report efficient synthetic routes to two symmetrical, dithienosilolo-central-unit-based A-D-A type organic semiconducting materials **DTS(Th**_**2**_**FBTTh)**_**2**_ and **DTS(ThFBTTh)**_**2**_. Fine-tuned conditions in Suzuki–Miyaura couplings were tested and utilized. The effect of inserting additional hexylthiophene structures symmetrically into the material backbone was investigated, and it was noted that contrary to commonly accepted fact, the distance between electron donor and acceptor seems to play a bigger role in lowering the *E*_gap_ value of the molecule than just extending the length of the conjugated backbone. We searched for precedent cases from the literature, and these are compared to our findings. The optical properties of the materials were characterized with UV–vis spectroscopy. Majority of the intermediate compounds along the way to final products were produced with excellent yields. Our results offer highly efficient routes to many heterocyclic structures but also give new insights into the design of organic semiconducting materials.

## Introduction

In the syntheses of conjugated organic semiconductor materials, the utilization of different types of coupling reactions is of paramount importance, as constructing the chain-like conjugated structures using alternative synthetic strategies would turn out to be an extremely laborious task. It can be safely stated that coupling reactions like Suzuki–Miyaura have laid the foundation for these types of organic molecules to the extent that can be seen today. In addition, Suzuki–Miyaura cross-coupling is an established and widely used tool of organic synthetic chemistry, especially in organic materials chemistry and medicinal chemistry.^1^ Akira Suzuki, the original developer of the Suzuki coupling, was one of the three scientists awarded with Nobel prize in chemistry in 2010 “for palladium-catalyzed cross couplings in organic synthesis”.^2^

Organic semiconductors have been successfully applied in organic light emitting diode (OLED) technology,^3^ organic biosensors,^4^ and organic field-effect transistors (OFET),^5^ as well as in organic photovoltaics (OPV), a topic that has drawn considerable research interest in recent times. This could be attributed to the many attractive properties of OPVs, e.g., ease of processing (inkjet printing, roll-to-roll processing) and the possibility to produce lightweight and flexible devices. Most of the aforementioned properties are common also with other organic semiconductor applications.

Ability to fine-tune the electronic and optical properties of materials is one of the greatest strengths of organic semiconductors. Alternating the donor–acceptor sequence in organic semiconductors has had a beneficial impact on HOMO–LUMO levels of the material and promoting, e.g., charge carrier properties.^6^ For example, in small molecular OPV active layers, the most successful composition has been acceptor–donor–acceptor, where the central part of the molecule was an electron donating moiety and end groups work as electron acceptors (also known as push–pull structure).^7^

Benzothiadiazole (BT) is an abundantly present building block among organic semiconductors. In addition, several structural modifications of benzothiadiazole in organic semiconductor applications can be found from the literature. For example, fluorination of benzothiadiazole (fluorobenzothiadiazole, FBT) fragment has been shown to be an effective way to lower HOMO and LUMO energy levels of the molecule.^8^ Another highly important moiety is thiophene which has extensively been used as a building block in organic semiconductors. In fact, thiophene can be found, as a separate fragment or part in a fused ring system, from most of the recently reported active layers of the high-performance OPVs.^9^ The first thiophene-containing semiconducting polymer to gain widespread popularity was P3HT (poly(3-hexylthiophene). On the other hand, the other pentacyclic heteroaromatic compound, thiazole (Tz), is far less utilized in organic semiconducting materials, even though it is present in many natural products, and it has found various uses in the fields of material and medicinal chemistry. Our previous studies showed that the thiazole unit had a major role in the regioselectivity of bromination reactions which could be affected by pH control.^10^

The utilization of the alkylated dithienosilole (DTS) moiety in OPV application dates to 2006 to the work of Usta et al.^11^ Even though the number of publications concerning dithienosilole as a central unit has been recently decreasing, dithienosilole moiety is still a valid building block when developing high-performance electron donors in OPV applications.^12^

In this paper, we report a synthetic route for two novel organic A-D-A type semiconductor materials, DTS(Th_2_FBTTh)_2_ and DTS(ThFBTTh)_2_. These molecules differ in structure so that DTS(Th_2_FBTTh)_2_ contains two additional, symmetrically placed *n*-hexylthiophene moieties. UV–vis spectroscopy was used to characterize the optical properties of the compounds. Differential scanning calorimetry was utilized to determine melting points and the thermal stability of the materials. Most of the intermediates along the synthetic routes could be produced with good to excellent yields. The developed procedures can be applied in fine-tuning the properties of small molecular semiconductors, as well as with their polymeric counterparts from the viewpoint of monomer synthesis strategies.

## Experimental Section

Commercial reagents were used as received. Compounds **2**, **8**, and **17** were synthesized as presented in our previous work.^10,13^ All molecules were characterized using Bruker 400 MHz NMR spectrometer, and samples were prepared using deuterated chloroform as a solvent, with TMS as an internal standard. All coupling constants are reported in Hz. ESI+ TOF MS characterizations were performed using Thermo Scientific QExactive mass spectrometer. All reactions were monitored with thin-layer chromatography using silica gel-coated aluminum sheets. UV–vis absorption spectra of compounds DTS(Th_2_FBTTh)_2_ and DTS(ThFBTTh)_2_ in chloroform (concentrations 7.5 μM) were measured in quartz glass cuvettes with Shimadzu UV-1800 spectrophotometer. A Mettler Toledo DSC (DSC 821e) was used to evaluate the melting temperatures of DTS(Th_2_FBTTh)_2_ and DTS(ThFBTTh)_2_. Experiments were run under inert gas flow (50 cm^3^/min N_2_) at a temperature range of 25–280 °C. The heat flow rate was 10 °C/min. Samples (ca. 1.6–3 mg) were weighed into 40 μL aluminum crucibles, which were closed with a pierced lid.

## Syntheses

### Synthesis of 5-Fluoro-7-(5-hexylthiophen-2-yl)-4-(thiophen-2-yl)-2,1,3-benzothiadiazole (**3**)

A mixture of toluene (3.0 mL), DMA (3.0 mL), distilled water (0.6 mL), and 2-thiopheneboronic acid pinacol ester (1.22 equiv, 87.0 mg, 0.41 mmol) were deoxygenated with argon gas for 20 min in a reaction tube with a magnetic stirring bar. Compound **2** (135.1 mg, 0.34 mmol), Cs_2_CO_3_ (2.61 equiv, 288.2 mg, 0.88 mmol), (*t*-Bu)_3_P·HBF_4_ (13 mol %, 12.4 mg, 42 μmol), and Pd_2_(dba)_3_ (4 mol %, 11.6 mg, 12.7 μmol) were added to the mixture. The sealed tube was evacuated and backfilled with argon five times. The reaction mixture was stirred and heated in an oil bath (100 °C) for 6 h. The reaction mixture was filtered through a thin pad of silica gel rinsing with toluene and evaporated under reduced pressure. The product was purified by using flash chromatography (SiO_2_, toluene 1:1 *n*-hexane). The isolated **3** was collected as a red solid (134.5 mg) in 99% yield. ^1^H NMR (400 MHz, CDCl_3_) δ ppm 0.88–0.94 (m, 3 H), 1.32–1.47 (m, 6 H), 1.77 (quin, *J* = 7.6 Hz, 2 H), 2.91 (t, *J* = 7.6 Hz, 2 H), 6.90 (d, *J* = 3.7 Hz, 1 H), 7.24–7.26 (m, 1 H), 7.56 (dd, *J* = 5.1, 1.1 Hz, 1 H), 7.71 (d, *J* = 13.0 Hz, 1 H), 7.99 (d, *J* = 3.7 Hz, 1 H), 8.26 (d, *J* = 3.7 Hz, 1 H). HRMS (ESI+, TOF) *m*/*z*: M^+^ Calcd for C_20_H_19_N_2_S_3_F 402.0689; Found 402.0679.

### Synthesis of 4-(5-Bromothiophen-2-yl)-5-fluoro-7-(5-hexylthiophen-2-yl)-2,1,3-benzothiadiazole (**4**)

Compound **3** (104.8 mg, 0.26 mmol) was dissolved in THF (17 mL) and NBS (1.1 equiv, 51.0 mg, 0.29 mmol) was added. The reaction mixture was stirred at room temperature for 20 h. The solvent was evaporated, and the crude product was subjected to flash chromatography (SiO_2_, toluene 1:2 *n*-heptane). Pure **4** was isolated as a red solid (104.2 mg) in 83% yield. ^1^H NMR (400 MHz, CDCl_3_) δ ppm 0.88–0.95 (m, 3 H), 1.33–1.47 (m, 6 H), 1.76 (quin, *J* = 7.5 Hz, 2 H), 2.89 (t, *J* = 7.6 Hz, 2 H), 6.89 (d, *J* = 3.7 Hz, 1 H), 7.18 (d, *J* = 4.0 Hz, 1 H), 7.65 (d, *J* = 13.1 Hz, 1 H), 7.96 (d, *J* = 3.7 Hz, 1 H), 7.98 (d, *J* = 4.2 Hz, 1 H). HMRS (ESI+, TOF) *m*/*z*: M^+^ Calcd for C_20_H_18_BrFN_2_S_3_ 479.9794; Found 479.9786.

### Synthesis of 5-Fluoro-4-(3′-hexyl[2,2′-bithiophen]-5-yl)-7-(5-hexylthiophen-2-yl)-2,1,3-benzothiadiazole (**5**)

A mixture of toluene (3.0 mL), ethanol (3.0 mL), distilled water (0.6 mL), and 3-hexyl-2-thiopheneboronic acid pinacol ester (1.1 equiv, 69.2 mg 0.24 mmol) was deoxygenated with argon gas for 15 min in a reaction tube with a magnetic stirring bar. Compound **4** (100.5 mg, 0.21 mmol), K_3_PO_4_ (3.0 equiv, 132.6 mg, 0.62 mmol), Xantphos (5 mol %, 6.2 mg, 10.7 μmol), and Pd(OAc)_2_ (5 mol %, 2.5 mg, 11.1 μmol) were added to the mixture. The sealed tube was evacuated and backfilled with argon five times. The reaction mixture was stirred and heated in an oil bath (100 °C) for 3 h. The reaction mixture was filtered through a thin pad of silica gel rinsing with toluene and evaporated under reduced pressure. The product was purified by using flash chromatography (SiO_2_, toluene 1:2 *n*-heptane). The isolated **5** was collected as a deep red solid (115.1 mg) in 97% yield. ^1^H NMR (400 MHz, CDCl_3_) δ ppm 0.87–0.94 (m, 6 H), 1.31–1.46 (m, 12 H), 1.66–1.73 (m, 2 H), 1.73–1.81 (m, 2 H), 2.84–2.88 (m, 2 H), 2.91 (t, *J* = 7.7 Hz, 2 H), 6.90 (d, *J* = 3.7 Hz, 1 H), 6.99 (d, *J* = 5.1 Hz, 1 H), 7.23 (d, *J* = 5.3 Hz, 1 H), 7.25 (dd, *J* = 4.0, 1.3 Hz, 1 H), 7.71 (d, *J* = 13.1 Hz, 1 H), 7.98 (d, *J* = 3.7 Hz, 1 H), 8.24 (d, *J* = 4.0 Hz, 1 H). HRMS (ESI+, TOF) *m*/*z*: M^+^ Calcd for C_30_H_33_N_2_S_4_F 568.1505; Found 568.1492.

### Synthesis of 4-(5′-Bromo-3′-hexyl[2,2′-bithiophen]-5-yl)-5-fluoro-7-(5-hexylthiophen-2-yl)-2,1,3-benzothiadiazole (**6**)

Compound **5** (52.3 mg, 91.9 μmol) was dissolved in THF (6.2 mL), and NBS (1.09 equiv, 18.3 mg, 0.10 mmol) was added. The reaction mixture was stirred at rt for 22 h. The solvent was evaporated, and the crude product was subjected to flash chromatography (SiO_2_, toluene 1:2 *n*-heptane). Pure **6** was isolated as a deep red solid (53.4 mg) in 90% yield. ^1^H NMR (400 MHz, CDCl_3_) δ ppm 0.90 (m, 6 H), 1.25–1.49 (m, 12 H), 1.64 (m, 2 H), 1.70–1.79 (m, 2 H), 2.71–2.81 (m, 2 H), 2.87 (t, *J* = 7.6 Hz, 2 H), 6.86 (d, *J* = 3.8 Hz, 1 H), 6.91 (s, 1 H), 7.13 (dd, *J* = 4.0, 1.3 Hz, 1 H), 7.62 (d, *J* = 13.2 Hz, 1 H), 7.93 (d, *J* = 3.7 Hz, 1 H), 8.16 (d, *J* = 3.9 Hz, 1 H). HRMS (ESI+, TOF) *m*/*z*: [M + H]^+^ Calcd for C_30_H_32_N_2_S_4_BrF 646.0610; Found 646.0595.

### Synthesis of 2,2′-[4,4-Bis(2-ethylhexyl)-4*H*-silolo[3,2-*b*:4,5-*b*′]bisthiene-2,6-diyl]bis(4,4,5,5-tetramethyl-1,3,2-dioxaborolane) (**7**)

Magnesium chips (2.5 equiv, 36.5 mg, 1.50 mmol) and an iodine crystal were added in a reaction tube with a magnetic stirring bar. The sealed reaction system was heated until iodine sublimed. The reaction system was allowed to cool to 25 °C after which the system was purged with argon for 5 min. 2,6-Dibromo-4,4-bis(2-ethylhexyl)-4*H*-silolo[3,2-*b*:4,5-*b*′]bisthiophene (350.0 mg, 0.61 mmol) was dissolved in dry THF (2.1 mL) and added through the septum in the reaction system. Under constant stirring, pinacolborane (2.2 equiv, 0.19 mL, 1.34 mmol) was added dropwise through the septum. The reaction mixture was stirred at 25 °C ca. 2 days (until the reaction solution turned from light green into dark brown). The reaction mixture was cooled with an ice bath and toluene (5 mL) was added. 2 M aqueous HCl (2 mL) was added dropwise, and the mixture was stirred for 10 min. During that period, the released H_2_ gas escaped from the reaction system through the open needle. The reaction mixture was extracted with toluene (2 × 5 mL). The combined organic layers were dried with Na_2_SO_4_, filtered, and evaporated to dryness. The product was isolated by using flash chromatography (SiO_2_, toluene/*n*-heptane 1:1). Evaporation afforded product **7** as a viscous oil (345.2 mg) in 85% yield. ^1^H NMR (400 MHz, CDCl_3_) δ ppm 0.76 (t, *J* = 7.4 Hz, 6H), 0.81 (t, *J* = 6.6 Hz, 6H), 0.92 (dd, *J* = 6.9, 2.3 Hz, 4H), 1.06–1.28 (m, 18H), 1.36 (s, 24H), 7.58 (s, 2H).

### Synthesis of 2,5-Bis(3-hexylthiophen-2-yl)-1,3-thiazole (**10**)

Toluene (7.5 mL), EtOH (2.5 mL), distilled water (2.5 mL), and 2-(5-hexyl-2-thienyl)-4,4,5,5-tetramethyl-1,3,2-dioxaborolane (2.15 equiv, 650.8 mg, 2.21 mmol) was deoxygenated for 15 min in a reaction tube with a magnetic stirring bar. 2,5-Dibromothiatzole (250.2 mg, 1.03 mmol), K_3_PO_4_ (4.8 equiv., 1.06 g, 4.99 mmol), Xantphos (2.5 mol %, 14.9 mg, 25.8 μmol), and Pd(OAc)_2_ (2.4 mol %, 5.6 mg, 24.9 μmol) were added to a reaction tube which was sealed, evacuated, and refilled with argon gas five times. The reaction mixture was stirred in an oil bath (100 °C) for 4 h. Crude product was filtered through a thin pad of silica gel rinsing with toluene and evaporated under reduced pressure. The product was purified by using flash chromatography (SiO_2_, toluene/*n*-heptane 3:2). Pure **10** was isolated as a yellow viscous liquid (414.1 mg) in 96% yield. ^1^H NMR (400 MHz, CDCl_3_) δ ppm: 0.85–0.91 (m, 6H), 1.28–1.38 (m, 10H), 1.40–1.47 (m, 2H), 1.59–1.66 (m, 2H), 1.67–1.74 (m, 2H), 2.73 (t, *J* = 7.7 Hz, 2H), 2.93 (t, *J* = 7.8 Hz, 2H), 6.95 (dd, *J* = 5.1, 1.2 Hz, 2H), 7.22 (d, *J* = 5.1 Hz, 1H), 7.28 (d, *J* = 5.1 Hz, 1H), 7.76 (s, 1H). ^13^C NMR (100.6 MHz, CDCl_3_) δ ppm 14.0, 14.1, 22.6, 29.2, 29.3, 29.3, 29.9, 30.0, 30.7, 31.6, 124.8, 126.2, 126.5, 130.0, 130.3, 130.5, 131.6, 140.3, 141.2, 142.2, 160.4. HRMS (ESI+, TOF) *m*/*z*: [M + H]^+^ Calcd for C_23_H_32_NS_3_ 418.1697; Found 418.1696.

### Synthesis of 5-(5-Bromo-3-hexylthiophen-2-yl)-2-(3-hexylthiophen-2-yl)-1,3-thiazole (**11a**) and 2,5-Bis(5-bromo-3-hexylthiophen-2-yl)-1,3-thiazole (**11b**)

Compound **10** (423.1 mg, 1.01 mmol) was dissolved in CHCl_3_ (16 mL), and NBS (1.07 equiv., 193.1 mg, 1.08 mmol) was added. Reaction mixture was placed in a sonicator for 4 h. Crude product was purified by flash chromatography (Si_2_O, toluene/*n*-heptane 3:2). Pure **11a** was isolated as a brownish viscous liquid (461.1 mg) in 92% yield. ^1^H NMR (400 MHz, CDCl_3_) δ ppm: 0.85–0.91 (m, 6H), 1.27–1.36 (m, 10H), 1.39–1.46 (m, 2H), 1.59 (quin, *J* = 7.5 Hz, 2H), 1.70 (quin, *J* = 7.6 Hz, 2H), 2.66 (t, *J* = 7.7 Hz, 2H), 2.92 (t, *J* = 7.8 Hz, 2H), 6.92 (s, 1H), 6.96 (d, *J* = 5.1 Hz, 1H), 7.30 (d, *J* = 5.1 Hz, 1H), 7.71 (s, 1 H). ^13^C NMR (100.6 MHz, CDCl_3_) δ ppm 14.0, 14.1, 22.6, 22.6, 29.1, 29.3, 29.3, 29.9, 30.1, 30.5, 31.6, 31.6, 111.6, 126.7, 127.7, 128.9, 130.5, 131.4, 132.6, 140.7, 141.9, 142.5, 160.8. HRMS (ESI+, TOF) *m*/*z*: [M + H]^+^ Calcd for C_23_H_31_NS_3_Br 496.0802; Found 496.0791. A small amount of **11b** was isolated for analytical purposes. ^1^H NMR (400 MHz, CDCl_3_) δ ppm: 0.87–0.92 (m, 6H), 1.27–1.45 (m, 12H), 1.56–1.72 (m, 4H), 2.66 (t, *J* = 8.0 Hz, 2H), 2.85 (t, *J* = 8.0 Hz, 2H), 6.94 (s, 1H), 6.94 (s, 1H), 7.69 (s, 1H). HRMS (ESI+, TOF) *m*/*z*: [M + H]^+^ Calcd for C_23_H_30_NS_3_Br_2_ 573.9901; Found 573.9922.

### Synthesis of 5-[3-Hexyl-5-(4,4,5,5-tetramethyl-1,3,2-dioxaborolan-2-yl)thiophen-2-yl]-2-(3-hexylthiophen-2-yl)-1,3-thiazole (**12**)

Magnesium chips (1.3 equiv, 35.3 mg, 1.45 mmol) and an iodine crystal were added in a reaction tube with a magnetic stirring bar. The sealed reaction system was heated until iodine sublimed. The reaction system was allowed to cool to 25 °C which after the system was purged with argon for 5 min. Compound **11a** (491.9 mg, 0.99 mmol) was dissolved in dry THF (2.8 mL) and added through the septum in the reaction system. Under constant stirring, pinacolborane (1.1 equiv, 0.16 mL, 1.10 mmol) was added dropwise through the septum. The reaction mixture was stirred at 25 °C overnight. The reaction mixture was cooled with ice bath, 2 M aqueous HCl (3 mL) was added dropwise, and the mixture was stirred for 10 min. During that period, the released H_2_ gas escaped from the reaction system through the open needle. The reaction mixture was extracted with toluene (3 × 10 mL). The combined organic layers were dried with Na_2_SO_4_, filtered, and evaporated to dryness. The product was isolated by using flash chromatography (SiO_2_). The column was eluated with toluene until the impurities run out. In the second stage, the column was eluated with acetone to isolate the desired product. Finally, evaporation gave compound **12** as a viscous oil (405.3 mg) in 75% yield. ^1^H NMR (400 MHz, CDCl_3_) δ ppm 0.86–0.91 (m, 6H), 1.30–1.45 (m, 24H), 1.62–1.74 (m, 4H), 2.76 (t, *J* = 7.8 Hz, 2H), 2.92 (t, *J* = 7.6 Hz, 2H), 6.95 (d, *J* = 5.1 Hz, 1H), 7.29 (d, *J* = 5.1 Hz, 1H), 7.49 (s, 1H), 7.84 (s, 1H). ^13^C NMR (100.6 MHz, CDCl_3_) δ ppm 14.0, 22.5, 22.5, 24.6, 29.1, 29.2, 29.8, 30.0, 30.5, 31.5, 31.5, 84.1, 126.5, 128.1, 130.3, 130.4, 131.4, 133.4, 139.9, 140.2, 142.0, 142.2, 160.4. HRMS (ESI+, TOF) *m*/*z*: [M + H]^+^ Calcd for C_29_H_43_NO_2_S_3_B 544.2549; Found 544.2536.

### Synthesis of 5-Fluoro-4-(4-hexylthiophen-2-yl)-7-(5-hexylthiophen-2-yl)-2,1,3-benzothiadiazole (**13**)

Compound **13** was synthesized from **2**, following the synthetic procedure for **3**. The specific amounts of chemicals were as follows: compound **2** (103.9 mg, 0.26 mmol), 2-(4-hexylthiophen-2-yl)-4,4,5,5-tetramethyl-1,3,2-dioxaborolane (1.2 equiv, 90.5 mg, 0.31 mmol), Cs_2_CO_3_ (2.5 equiv, 210.7 mg, 0.65 mmol), (*t*-Bu)_3_P·HBF_4_ (12 mol %, 8.9 mg, 31 μmol), and Pd_2_(dba)_3_ (3.6 mol %, 8.5 mg, 9.3 μmol). The method afforded **13** as a red solid (124.3 mg) in 98% yield. ^1^H NMR (400 MHz, CDCl_3_) δ ppm: 0.90–0.93 (m, 6H), 1.32–1.45 (m, 12H), 1.68–1.80 (m, 4H), 2.72 (t, *J* = 7.7 Hz, 2H), 2.90 (t, *J* = 7.6 Hz, 2H), 6.89 (d, *J* = 3.8 Hz, 1H), 7.15 (d, *J* = 1.1 Hz, 1H), 7.68 (d, *J* = 13.1 Hz, 1H), 7.96 (d, *J* = 3.7 Hz, 1 H), 8.09 (d, *J* = 0.9 Hz, 1 H). HRMS (ESI+, TOF) *m*/*z*: [M + H]^+^ Calcd for C_26_H_32_N_2_S_3_F 487.1706; Found 487.1698.

### Synthesis of 4-(5-Bromo-4-hexylthiophen-2-yl)-5-fluoro-7-(5-hexylthiophen-2-yl)-2,1,3-benzothiadiazole (**14**)

Compound **13** (164.5 mg, 0.34 mmol) was dissolved in THF (27 mL), and NBS (1.1 equiv., 67.5 mg, 0.38 mmol) was added. Reaction mixture was placed in a sonicator for 50 min. The solvent was evaporated, and the crude product was purified by flash chromatography (Si_2_O, toluene/*n*-hexane 1:5). Pure **14** was isolated as a red solid (181.4 mg) in 95% yield. ^1^H NMR (400 MHz, CDCl_3_) δ ppm: 0.90–0.93 (m, 6H), 1.32–1.45 (m, 12H), 1.68 (quin, *J* = 7.5 Hz, 2H), 1.76 (quin, *J* = 7.5 Hz, 2H), 2.66 (t, *J* = 7.6 Hz, 2H), 2.89 (t, *J* = 7.6 Hz, 2H), 6.89 (d, *J* = 3.8 Hz, 1H), 7.64 (d, *J* = 13.2 Hz, 1H), 7.93 (s, 1 H), 7.96 (d, *J* = 3.7 Hz, 1 H). HRMS (ESI+, TOF) *m*/*z*: [M + H]^+^ Calcd for C_26_H_31_N_2_S_3_FBr 565.0811; Found 565.0805.

### Synthesis of 5-Fluoro-4-{4-hexyl-5-[2-(3-hexylthiophen-2-yl)-1,3-thiazol-5-yl]thiophen-2-yl}-7-(5-hexylthiophen-2-yl)-2,1,3-benzothiadiazole (**15**)

Compound **15** was synthesized from **14**, following the synthetic procedure for **3**. The specific amounts of chemicals were as follows: compound **14** (152.8 mg, 0.27 mmol), 2-(3-hexylthiophen-2-yl)-5-(4,4,5,5-tetramethyl-1,3,2-dioxaborolan-2-yl)-1,3-thiazole (1.1 equiv, 108.6 mg, 0.29 mmol), Cs_2_CO_3_ (2.5 equiv, 218.3 mg, 0.67 mmol), (*t*-Bu)_3_P·HBF_4_ (12 mol %, 9.5 mg, 33 μmol), and Pd_2_(dba)_3_ (4.8 mol %, 12.3 mg, 13 μmol). The method afforded **15** as a deep red solid (163.0 mg) in 82% yield. ^1^H NMR (400 MHz, CDCl_3_) δ ppm 0.89–0.93 (m, 9H), 1.32–1.49 (m, 18H), 1.69–1.80 (m, 6H), 2.84 (t, *J* = 8.1 Hz, 2H), 2.89 (t, *J* = 7.6 Hz, 2H), 2.97 (t, *J* = 7.8 Hz, 2H), 6.89 (d, *J* = 3.8 Hz, 1H), 6.99 (d, *J* = 5.1 Hz, 1H), 7.33 (d, *J* = 5.1 Hz, 1H), 7.67 (d, *J* = 13.2 Hz, 1H), 7.90 (s, 1H), 7.98 (d, *J* = 3.7 Hz, 1H), 8.11 (s, 1H). HRMS (ESI+, TOF) *m*/*z*: [M + H]^+^ Calcd for C_39_H_47_N_3_S_5_F 736.2352; Found 736.2332.

### Synthesis of 4-{5-[2-(5-Bromo-3-hexylthiophen-2-yl)-1,3-thiazol-5-yl]-4-hexylthiophen-2-yl}-5-fluoro-7-(5-hexylthiophen-2-yl)-2,1,3-benzothiadiazole (**16**)

Compound **15** (114.1 mg, 0.16 mmol) was dissolved in THF (9.7 mL), and NBS (1.06 equiv, 30.9 mg, 0.17 mmol) was added. The stirring of the reaction mixture was initiated at 0 °C and the temperature was allowed to rise to room temperature over the next 24 h. The solvent was evaporated, and the crude product was subjected to flash chromatography (SiO_2_, toluene 2:1 *n*-hexane). Pure **16** was isolated as a deep red solid (70.6 mg) in 56% yield. ^1^H NMR (400 MHz, CDCl_3_) δ ppm 0.89–0.93 (m, 9H), 1.32–1.47 (m, 18H), 1.67–1.80 (m, 6H), 2.82–2.92 (m, 6H), 6.90 (d, *J* = 3.7 Hz, 1H), 6.96 (s, 1H), 7.70 (d, *J* = 13.2 Hz, 1H), 7.88 (s, 1H), 7.99 (d, *J* = 3.8 Hz, 1H), 8.12 (s, 1H). HRMS (ESI+, TOF) *m*/*z*: [M + H]^+^ Calcd for C_39_H_46_N_3_S_5_FBr 814.1457; Found 814.1450.

### Synthesis of 4,4′-{[4,4-Bis(2-ethylhexyl)-4*H*-silolo[3,2-*b*:4,5-*b*′]bis-thiene-2,6-diyl]bis[(3′-hexyl[2,2′-bithiophene]-5′,5-diyl)]}bis[5-fluoro-7-(5-hexylthiophen-2-yl)-2,1,3-benzothiadiazole] (**DTS(Th**_**2**_**FBTTh)**_**2**_)

Compound **7** (13.6 mg, 20 μmol) was added into a reaction tube with a magnetic stirring bar, along with ethanol (0.15 mL), distilled water (0.1 mL), and toluene-Pd(OAc)_2_ solution (0.85 mL, corresponding to 10 mol %, 400 μg, 2 μmol of Pd(OAc)_2_). The solution was deoxygenated with argon for 15 min. Compound **6** (2.0 equiv, 25.9 mg, 40 μmol), Na_2_CO_3_ (5.5 equiv, 11.7 mg, 0.11 mmol), and Xantphos (10 mol %, 1.2 mg, 2 μmol) were added in the reaction system. The sealed tube was evacuated and backfilled with argon five times. The reaction mixture was stirred and heated in an oil bath (80 °C) for 4 h. The reaction mixture was filtered through a thin pad of silica gel rinsing with toluene and evaporated under reduced pressure. The crude product was purified by using flash chromatography (SiO_2_, toluene 1:5 *n*-hexane) and washed with 4 × 5 mL methanol, 3 × 5 mL acetone, and 2 × 5 mL methanol. The isolated **DTS(Th**_**2**_**FBTTh)**_**2**_ was collected as a dark purple solid (18.2 mg) in 59% yield. Mp 133 °C. ^1^H NMR (400 MHz, CDCl_3_) δ ppm 0.81–1.06 (m, 28H), 1.21–1.48 (m, 42H), 1.71–1.80 (m, 8H), 2.84–2.92 (m, 8H), 6.90 (d, *J* = 3.7 Hz, 2H), 7.03 (s, 2H), 7.16 (s, 2H), 7.26 (s, 2H), 7.71 (d, *J* = 13.1 Hz, 2H), 7.98 (d, *J* = 3.7 Hz, 2H), 8.25 (d, *J* = 3.9 Hz, 2H). HRMS (ESI+, TOF) *m*/*z*: [M + H]^+^ Calcd for C_84_H_101_N_4_S_10_F_2_Si 1551.4915; Found 1551.4923.

### Synthesis of 4,4′-{[4,4-Bis(2-ethylhexyl)-4*H*-silolo[3,2-*b*:4,5-*b*′]bisthiene-2,6-diyl]di(thiene-5,2-diyl)}bis[5-fluoro-7-(5-hexylthiophen-2-yl)-2,1,3-benzothiadiazole] (**DTS(ThFBTTh)**_**2**_)

**DTS(ThFBTTh)**_**2**_ was synthesized from **4**, following the synthetic procedure for **DTS(Th**_**2**_**FBTTh)**_**2**_. The specific amounts of chemicals were as follows: compound **7** (14.3 mg, 21 μmol), ethanol (0.15 mL), distilled water (0.1 mL) and toluene-Pd(OAc)_2_ -solution (0.85 mL, corresponding to 8 mol %, 400 μg, 2 μmol of Pd(OAc)_2_), compound **4** (2.1 equiv, 21.7 mg, 45 μmol), Na_2_CO_3_ (6.2 equiv, 13.4 mg, 0.13 mmol), and Xantphos (17 mol %, 2.0 mg, 3.5 μmol). The crude product was purified by using flash chromatography (SiO_2_, dichloromethane 2:5 *n*-hexane) and washed with total volume of 30 mL of methanol and 70 mL of acetone. The isolated **DTS(ThFBTTh)**_**2**_ was collected as a dark purple solid (12.1 mg) in 47% yield. Mp 164 °C. ^1^H NMR (400 MHz, CDCl_3_) δ ppm 0.82–0.94 (m, 18H), 0.98–1.09 (m, 4H), 1.22–1.45 (m, 30 H), 1.77 (quin, *J* = 7.6 Hz 4H), 2.90 (t, *J* = 7.6 Hz, 4H), 6.89 (d, *J* = 3.7 Hz, 2H), 7.26 (dd, *J* = 1.1 Hz, 2H), 7.30 (s, 2H), 7.69 (d, *J* = 13.2 Hz, 2H), 7.97 (d, *J* = 3.7 Hz, 2H), 8.19 (d, *J* = 4.0 Hz, 2H). HRMS (ESI+, TOF) *m*/*z*: M^+^ Calcd for C_64_H_72_N_4_S_8_F_2_Si 1218.3254; Found 1218.3232.

## Results and Discussion

### Syntheses

**Scheme 1:**
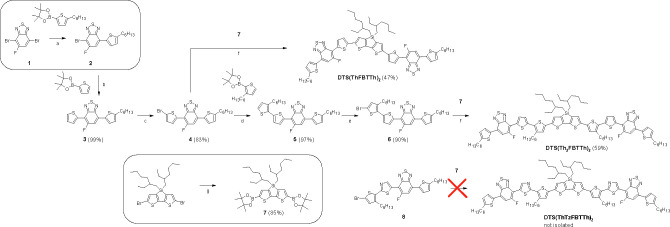


Compound **2** (Scheme 1) was received from 4,7-dibromo-5-fluoro-2,1,3-benzothiadiazole (**1**) using a selective Suzuki–Miyaura cross-coupling procedure developed by our group.^13^ The method relies on an efficient Pd(OAc)_2_ and Xantphos catalyst system. By contrast, the same catalyst system gave **3** only in 50% conversion in 22 h in the consequent reaction step where compound **2** was utilized as a starting material. After short optimizations, compound **3** was synthesized in nearly quantitative yield (99%) from **2** using Pd_2_(dba)_3_ and (*t*-Bu)_3_P·HBF_4_ as a catalyst system of Suzuki–Miyaura cross-coupling. Bromination of **3** with NBS in THF gave **4** in good yield (83%) which underwent Suzuki–Miyaura cross-coupling affording **5** in high yield (97%) in the presence of Pd(OAc)_2_/Xantphos catalyst system. A consequential reaction with NBS gave **6** in high yield (90%).

**Scheme 2:**
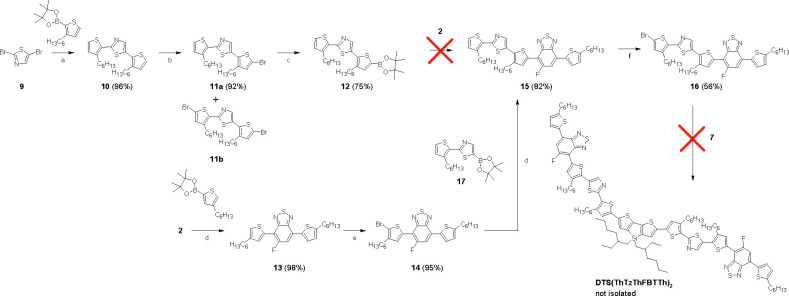


Compound **7** (Scheme 1) has previously been synthesized in 65% yield utilizing *n*-BuLi and 2-isopropoxy-4,4,5,5-tetramethyl-1,3,2-dioxaborolane (IPTMDOB).^14^ The method developed by Singaram et al.,^15^ which our group showed to be a reliable method to synthesize 2-(3-hexylthiophen-2-yl)-5-(4,4,5,5-tetramethyl-1,3,2-dioxaborolan-2-yl)-1,3-thiazole^10^ (compound **17**, Scheme 2), gave **7** in highly improved yield (85%). However, the utilization of **7** as a building block proved to be troublesome. Three different catalyst systems Pd(OAc)_2_/Xantphos, Pd_2_(dba)_3_/(*t*-Bu)_3_P·HBF_4_, and a special catalyst for sensitive boronic acids, XPhosPdG2, with aqueous Cs_2_CO_3_ in toluene/DMA not only catalyzed the desired Suzuki–Miyaura cross-coupling between **6** and **7** but also led to simultaneous hydrolysis of dithienosilole unit. The possible lability of dithienosilole in the presence of an aqueous base has previously been reported by Nguyen et al.^16^ Compound **6** did not show any conversion in the presence of Pd(OAc)_2_/Xantphos in dioxane/H_2_O. In toluene/DMA (anhydrous conditions), the same catalyst system showed formation of multiple byproducts. Previously, Pd(PPh_3_)_4_ along with aqueous Na_2_CO_3_ in dimethoxyethane (DME) has shown relatively good catalytic activity with compound **7** and its derivatives during polymer syntheses.^14,17^ In toluene/ethanol, Pd(PPh_3_)_4_ along with aqueous Na_2_CO_3_ showed only minor catalytic activity in the Suzuki–Miyaura cross-coupling between **6** and **7**. However, signs from hydrolysis of dithienosilole unit could not be observed anymore. The desired compound **DTS(Th**_**2**_**FBTTh)**_**2**_ was finally received in 59% yield using Pd(OAc)_2_/Xantphos with aqueous Na_2_CO_3_ in toluene/ethanol. The same reaction system gave compound **DTS(ThFBTTh)**_**2**_ in 47% yield in the reaction between **7** and **4**. However, the method was inefficient for Suzuki–Miyaura cross-coupling between compounds **7** and **8**, and the target product **DTS(ThTzFBTTh)**_**2**_ could not be separated from the crude reaction mixture that also contained unreacted **8** and debrominated form of compound **8**. Compound **8** has previously shown efficient reactivity in Suzuki-Miayura cross-couplings in the presence of Pd(OAc)_2_/Xantphos with aqueous Cs_2_CO_3_ in toluene/DMA.^13^ The cross-coupling experiments carried out here underline the importance of delicate fine-tuning of reaction conditions. The results here and our previous results^10,13^ show that in most cases Pd(OAc)_2_/Xantphos is an extremely efficient catalyst system in Suzuki–Miyaura to construct C–C bonds between thiophene, thiazole, benzothiadiazole, and carbazole units. For unreactive C–Br bonds such as in compound **2**, Pd_2_(dba)_3_/(*t*-Bu)_3_P·HBF_4_ catalyst system can be the right choice.

Compound **DTS(ThTzThFBTTh)**_**2**_ (Scheme 2) was selected for the next target after facing problems with the synthesis of compound **DTS(ThTzFBTTh)**_**2**_ (Scheme 1). To achieve the target, needed building blocks had to be designed and synthesized. Suzuki–Miyaura cross-coupling between **9** and 2-(3-hexylthiophen-2-yl)-4,4,5,5-tetramethyl-1,3,2-dioxaborolane gave **10** in high yield (96%). The bromination of **10** with NBS in an ultrasonic bath gave selectively monobrominated product **11a** in high yield (92%). The chemical structure of **11a** was confirmed by ^1^H, ^13^C, and 2D NMR measurements (see Supporting Information) to verify the site of bromination. A small amount of dibrominated byproduct **11b** along with the unreacted starting material **10** could be separated from the product mixture. The borylation method applied for the synthesis of **7** was also efficient for **11a** affording **12** in good yield (75%). Unfortunately, several attempted Suzuki–Miyaura cross-couplings between **12** and **2** were unsuccessful, and the desired product **15** could not be separated. Based on TLC and NMR analyses, the major problem seems to be unwanted deborylation of **12** in the presence of Pd_2_(dba)_3_ and (*t*-Bu)_3_P·HBF_4_ or Xantphos. XPhosPdG2, which has previously been shown to act as an efficient catalyst for labile pinacol esters, showed only low conversion of **2** into product **15** at 30 °C. Increasing temperature up to 60 °C was shown to facilitate deborylation of **12**.

The successful route to **15** was developed starting with cross-coupling between **2** and 2-(4-hexylthiophen-2-yl)-4,4,5,5-tetramethyl-1,3,2-dioxaborolane that afforded **13** in high yield (98%). Consequent ultrasonic supported bromination and Suzuki–Miyaura reactions afforded **14** and **15** in 95 and 82% yields, respectively. Next, bromination of **15** and flash-chromatographic separation gave monobrominated **16** in medium yield (56%). TLC analysis implied that compound **15** has a strong tendency to give several different bromination products which are challenging to separate. Finally, **16** showed complete unreactivity with **7** under Suzuki–Miyaura reaction conditions with Pd(OAc)_2_ or Pd_2_(dba)_3_ and (*t*-Bu)_3_P·HBF_4_ or Xantphos as a catalyst system with aqueous Na_2_CO_3_ or Cs_2_CO_3_ in toluene/EtOH. In the presence of Pd(OAc)_2_ with either Xantphos or (*t*-Bu)_3_P·HBF_4_, the cross-coupling reaction did not proceed at all, and with Pd_2_(dba)_3_ and (*t*-Bu)_3_P·HBF_4_ catalyst system complete debromination of **16** was observed in few hours. Thus, compound **DTS(ThTzThFBTTh)**_**2**_ remained as an unachieved target.

### Differential Scanning Calorimetry

To determine the melting temperatures and heat stability of **DTS(Th**_**2**_**FBTTh)**_**2**_ and **DTS(ThFBTTh)**_**2**_, DSC measurements were performed for the materials. DSC curves of the materials can be found in Supporting Information (S53 and S54). **DTS(ThFBTTh)**_**2**_ showed a higher melting temperature of 164 °C compared to the melting temperature of **DTS(Th**_**2**_**FBTTh)**_**2**_, which was measured to be 133 °C. This result can be rationalized by the lack of two *n*-hexyl chains in **DTS(ThFBTTh)**_**2**_, which could be responsible for better intermolecular π–π -interactions of the material.

After the DSC measurements, ^1^H NMR spectra from both material samples were recorded, and it was noted that heating under an inert atmosphere to 280 °C had no effect on the integrity of the materials. Thus, the materials can withstand temporary heating to 280 °C under an inert atmosphere without degradation, far exceeding the temperatures encountered in, e.g., typical OPV applications.

### UV–vis Measurements

**Figure 1:**
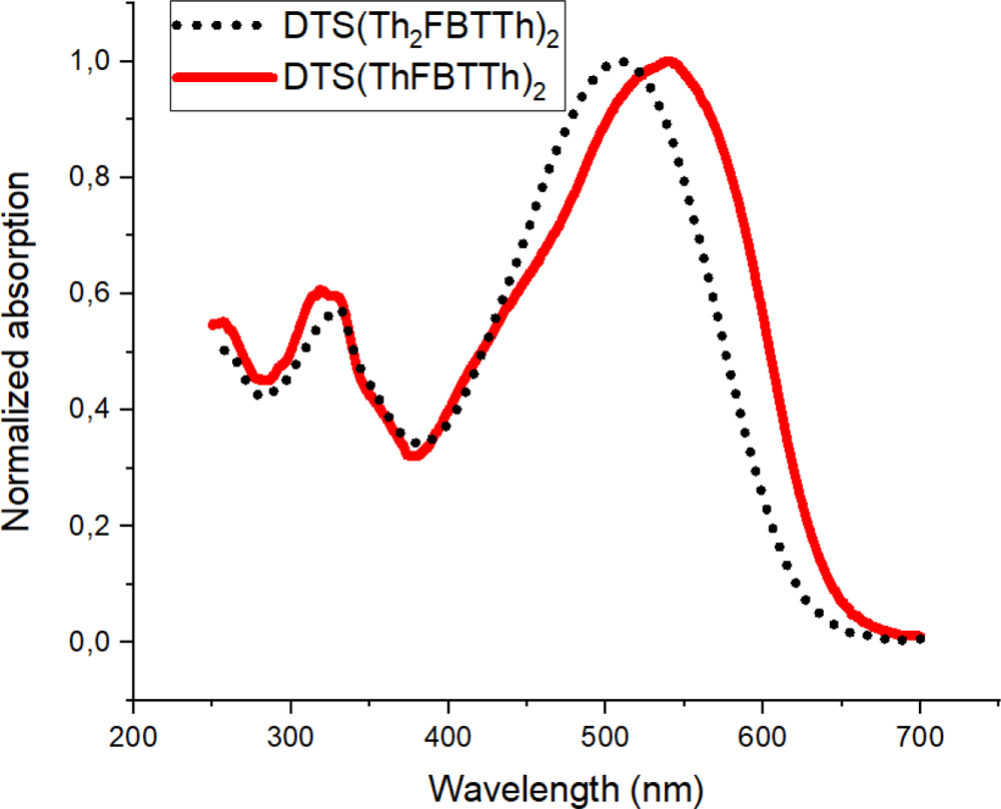
Normalized absorption spectra of compounds **DTS(Th**_**2**_**FBTTh)**_**2**_ and **DTS(ThFBTTh)**_**2**_ in CHCl_3_.

In order to study the effect of structural variation on electrical and spectral properties, UV–vis absorption spectra of **DTS(Th**_**2**_**FBTTh)**_**2**_ and **DTS(ThFBTTh)**_**2**_ were measured with a spectrophotometer. The normalized absorption spectra of the compounds in CHCl_3_ solution are presented in Figure 1. **DTS(Th**_**2**_**FBTTh)**_**2**_ shows an absorption maximum at 510 nm, whereas the absorption maximum of **DTS(ThFBTTh)**_**2**_ is red-shifted 30 nm located at 540 nm.

Optical band gaps (*E*_gap_) were determined from the absorption edges of the lowest energy absorption band.^18^ The calculated values were 2.00 and 1.93 eV for compounds **DTS(Th**_**2**_**FBTTh)**_**2**_ and **DTS(ThFBTTh)**_**2**_, respectively. In Table 1, the results are compared with the previous studies found in the literature.^19−22^ The observed *E*_gap_ values are higher compared to the value of **DTS(FBTTh**_**2**_**)**_**2**_ (1.85 eV). By comparing the results of **DTS(Th**_**2**_**FBTTh)**_**2**_ and **DTS(ThFBTTh)**_**2**_ to the results of DTS(FBTTh_2_)_2_ (entries 1–3), it can be observed, in addition, that both absorption maxima and edges are blue-shifted with the increasing distance of DTS and FBT units which results in simultaneous increase of *E*_gap_ values. On the other hand, *E*_gap_ can be decreased by increasing the number of thiophene (Th) end units as demonstrated in the series of DTS(PTTh)_2_, DTS(PTTh_2_)_2_, and DTS(PTTh_3_)_2_ (entries 4–6). The series of DTGe(FBTTh_2_)_2_, DTGe(FBTTh_3_)_2_, and DTGe(ThFBTTh_2_)_2_ (entries 7–9) show also that *E*_gap_ can be decreased by increasing the Th end units and decreasing the distance between central donor unit (DTGe) and electron accepting FBT units, even though the effect is much less pronounced. For benzodithiophene (BDT) central donors (entries 10–16), this effect seems to significantly increase with smaller distances between central donor and acceptors as in the case of the DTS central unit.^23,24^ From the results presented here and others found from literature, it can be concluded that bringing the acceptor moiety closer to the central donor unit by shortening the intermediate π-bridge decreases the *E*_gap_-value of the compound most efficiently.

**Table 1:**
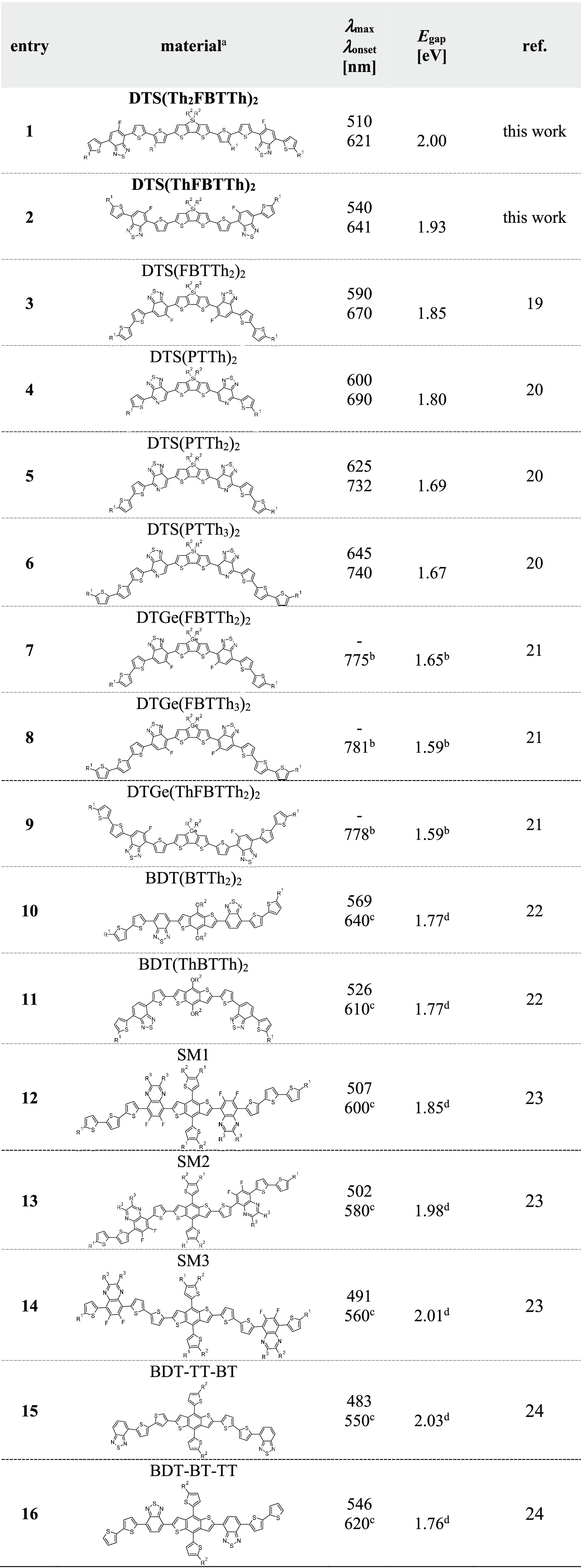
Comparison of Optical Properties of Presented and Literature-Reported A-D-A Type Organic Semiconductors^a^

a R^1^ = *n*-hexyl, R^2^ = 2-ethylhexyl, R^3^ = *n*-butyl. ^b^Determined with cyclic voltammetry. ^c^Estimated from presented UV–vis spectra. ^d^Calculated from onset of UV–vis absorption of thin-films.

## Conclusions

In summary, two novel A-D-A type organic semiconductors were designed and synthesized. Incorporation of additional thiophene spacers into the backbone structure led to higher *E*_gap_ value, thus decreasing the absorption onset wavelength. Lowering the optical band gap is considered beneficial in most applications, and these results suggest that a careful optimization of the moiety sequence in organic semiconductors plays a potentially larger role in decreasing the optical band gap than the overall size of the conjugated structure alone.

Even though only two out of four target compounds were eventually isolated, a significant amount of information concerning optimized synthetic procedures for novel building blocks was produced along the way. Our findings offer efficient routes to access a variety of small molecular units indispensable especially in the field of organic semiconductors.

## Supporting Information Available

NMR spectra of synthesized compounds with assignments and DSC curves of compounds **DTS(Th**_**2**_**FBTTh)**_**2**_ and **DTS(ThFBTTh)**_**2**_ (PDF)
