# Supplementary material for: Syntheses of Thiophene and Thiazole-Based Building Blocks and Their Utilization in the Syntheses of A-D-A Type Organic Semiconducting Materials with Dithienosilolo Central Unit
Source: ACS Omega. 2022 Jul 21;7(30):26328–35. doi: 10.1021/acsomega.2c02195 (PMC9352338; doi:10.1021/acsomega.2c02195)

SUPPORTING INFORMATION

Syntheses of thiophene and thiazole-based building blocks and their utilization in the syntheses of A-D-A type organic semiconducting materials with dithienosilolo central unit

*Tomi A. O. Parviainen, Petri M. Salmela, Roosa J. Sippola, and Juha P. Heiskanen**
Research Unit of Sustainable Chemistry, University of Oulu, P.O. Box 4300, FI-90014 Oulu, Finland
**Corresponding Author: juha.heiskanen@oulu.fi*

Contents:

^{1}H NMR spectrum of 3 in $CDCl_3$ S2
Local zoom of assigned ^{1}H NMR spectrum of 3 .... S3
H peak list and assignments of compound 3 .... S4
^{1}H NMR spectrum of 4 in $CDCl_3$ S5
Local zoom of assigned ^{1}H NMR spectrum of 4 .... S6
H peak list and assignments of compound 4 .... S7
^{1}H-^{1}H 2D NOE NMR of compound 4 in $CDCl_3$ S8
^{1}H NMR spectrum of 5 in $CDCl_3$ S9
Local zoom of assigned ^{1}H NMR spectrum of 5 .... S10
H peak list and assignments of compound 5 .... S11
^{1}H NMR spectrum of 6 in $CDCl_3$ S12
Local zoom of assigned ^{1}H NMR spectrum of 6 .... S13
H peak list and assignments of compound 6 .... S14
^{1}H-^{1}H 2D NOE NMR of compound 6 in $CDCl_3$ S15-S16
^{1}H NMR spectrum of 7 in $CDCl_3$ S17
^{1}H NMR spectrum of 10 in $CDCl_3$ S18
Local zoom of assigned ^{1}H NMR spectrum of 10 .... S19
^{13}C NMR spectrum of 10 in $CDCl_3$ S20
Peak list of ^{13}C NMR spectrum of 10 in $CDCl_3$ S21
^{1}H NMR spectrum of 11a in $CDCl_3$ S22
Local zoom of assigned ^{1}H NMR spectrum of 11a S23-S24
^{13}C NMR spectrum of 11a in $CDCl_3$ S25
Peak list of ^{13}C NMR spectrum of 11a in $CDCl_3$ S26
Local zoom of HSQC spectrum of 11a with assignments S27
^{13}C DEPT45 and the local zoom of HSQC spectrum of 11a S28
HMBC spectrum of 11a S29
Local zoom and peak list of assigned HMBC spectrum of 11a S30-S32
^{1}H NMR spectrum of 11b in $CDCl_3$ S33
Local zoom of assigned ^{1}H NMR spectrum of 11b S34
^{1}H NMR spectrum of 12 in $CDCl_3$ S35
Local zoom of assigned ^{1}H NMR spectrum of 12 .... S36
^{13}C NMR spectrum of 12 in $CDCl_3$ S37
Peak list of ^{13}C NMR spectrum of 12 in $CDCl_3$ S38
^{1}H NMR spectrum of 13 in $CDCl_3$ S39
Local zoom of assigned ^{1}H NMR spectrum of 13 .... S40
^{1}H NMR spectrum of 14 in $CDCl_3$ S41
Local zoom of assigned ^{1}H NMR spectrum of 14 .... S42
^{1}H NMR spectrum of 15 in $CDCl_3$ S43
Local zoom of assigned ^{1}H NMR spectrum of 15 .... S44
^{1}H NMR spectrum of 16 in $CDCl_3$ S45
Local zoom of assigned ^{1}H NMR spectrum of 16 .... S46
^{1}H NMR spectrum of compound DTS(Th_2FBTTh$)_2$ in $CDCl_3$ S47
Local zoom of assigned ^{1}H NMR spectrum of compound DTS(Th_2FBTTh$)_2$ in $CDCl_3$ S48
^{1}H NMR spectrum of compound DTS(Th_2FBTTh$)_2$ in CD_2Cl_2 .... S49
Local zoom of assigned ^{1}H NMR spectrum of compound DTS(Th_2FBTTh$)_2$ in CD_2Cl_2 .... S50
^{1}H NMR spectrum of compound DTS(ThFBTTh$)_2$ in $CDCl_3$ S51
Local zoom of assigned ^{1}H NMR spectrum of compound DTS(ThFBTTh$)_2$ in $CDCl_3$ S52
Differential scanning calorimetry curve of DTS(Th_2FBTTh$)_2$ S53
Differential scanning calorimetry curve of DTS(ThFBTTh$)_2$ S54

Figure S1. ^{1}H NMR spectrum of 3 in $CDCl_3$

J628.001.001.1r.esp

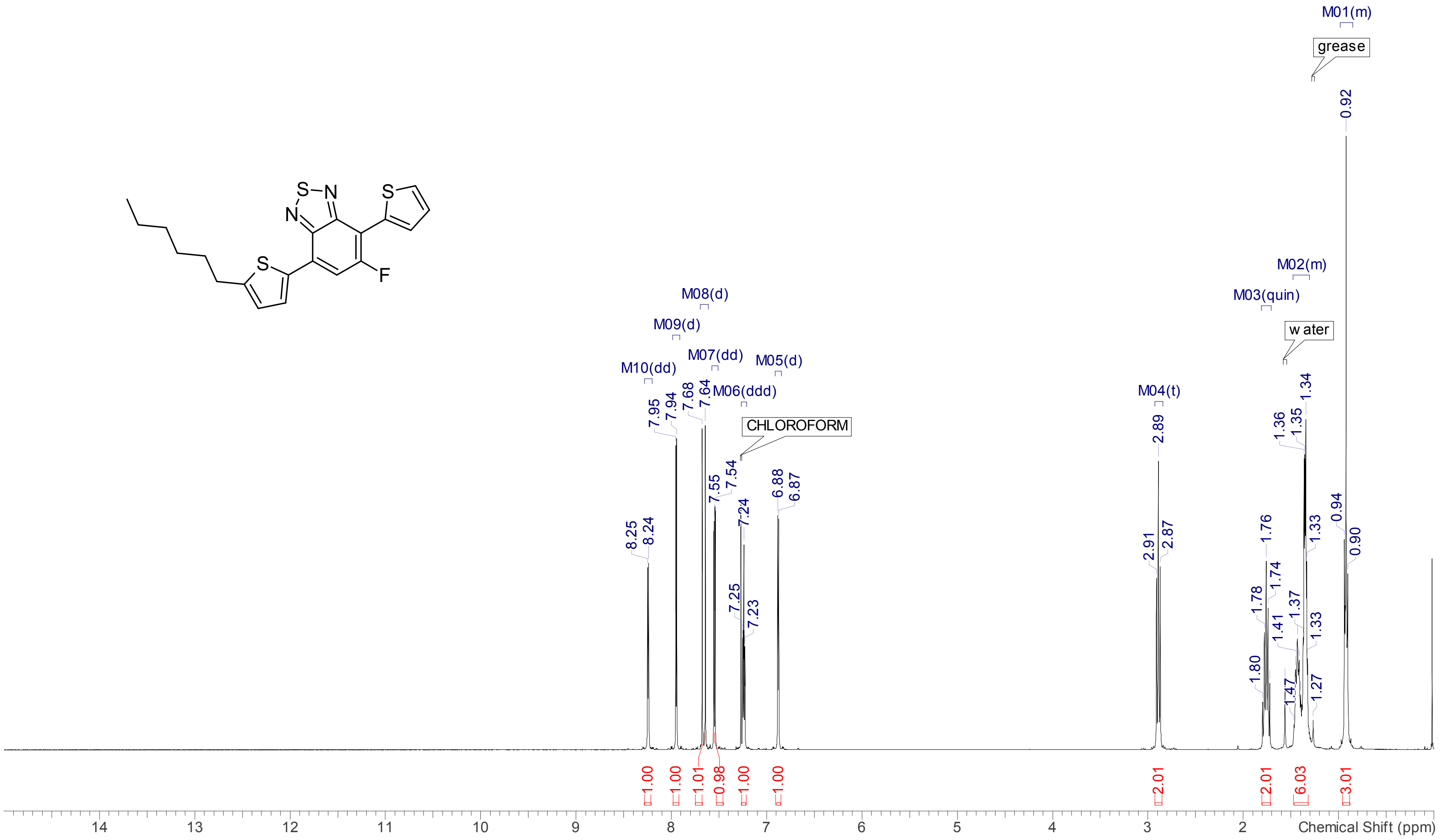

Figure S2. Local zoom of assigned ^{1}H NMR spectrum of 3

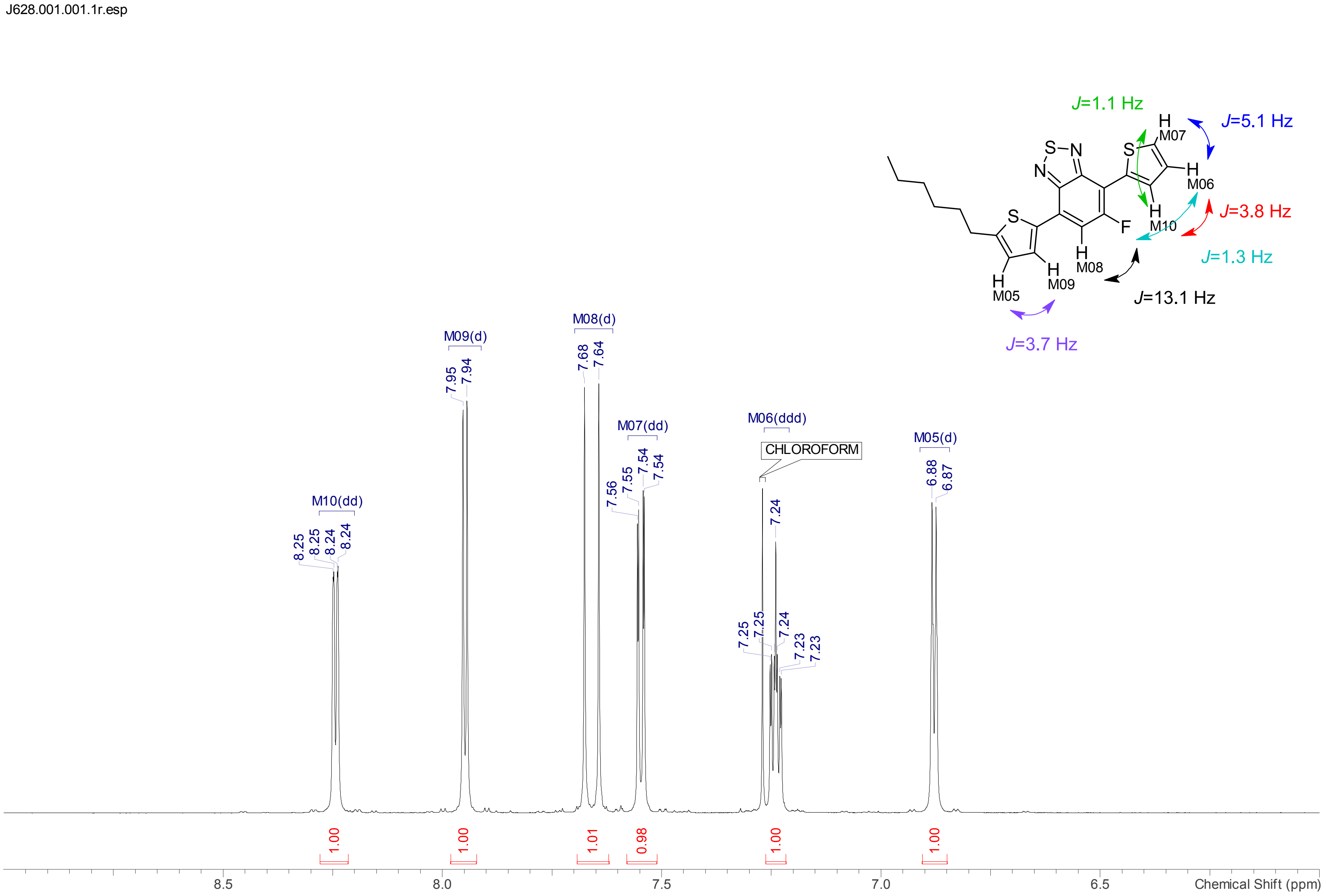

Figure S3. H peak list and assignments of compound 3

No.	Shift1 (ppm)	H's	Type	J (Hz)	Multiplet1	(ppm)
1	0.92	3	m	-	M01	[0.85 .. 0.98]
2	1.37	6	m	-	M02	[1.31 .. 1.48]
3	1.76	2	quin	7.55	M03	[1.71 .. 1.81]
4	2.89	2	t	7.64	M04	[2.84 .. 2.93]
5	6.88	1	d	3.79	M05	[6.84 .. 6.91]
6	7.24	1	ddd	5.14, 3.79,	M06	[7.21 .. 7.27]
7	7.55	1	dd	5.14, 1.10	M07	[7.51 .. 7.58]
8	7.66	1	d	13.08	M08	[7.61 .. 7.70]
9	7.95	1	d	3.67	M09	[7.91 .. 7.99]
10	8.24	1	dd	3.79, 0.61	M10	[8.20 .. 8.28]

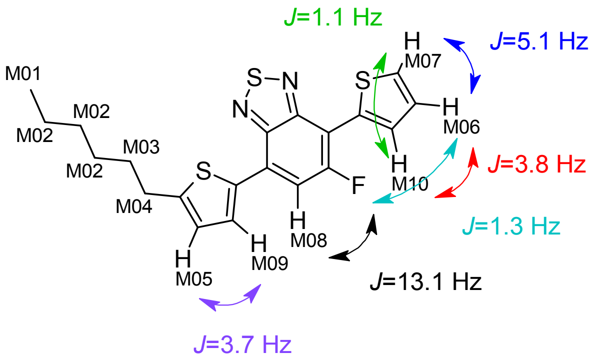

Figure S4. ^{1}H NMR spectrum of 4 in $CDCl_3$

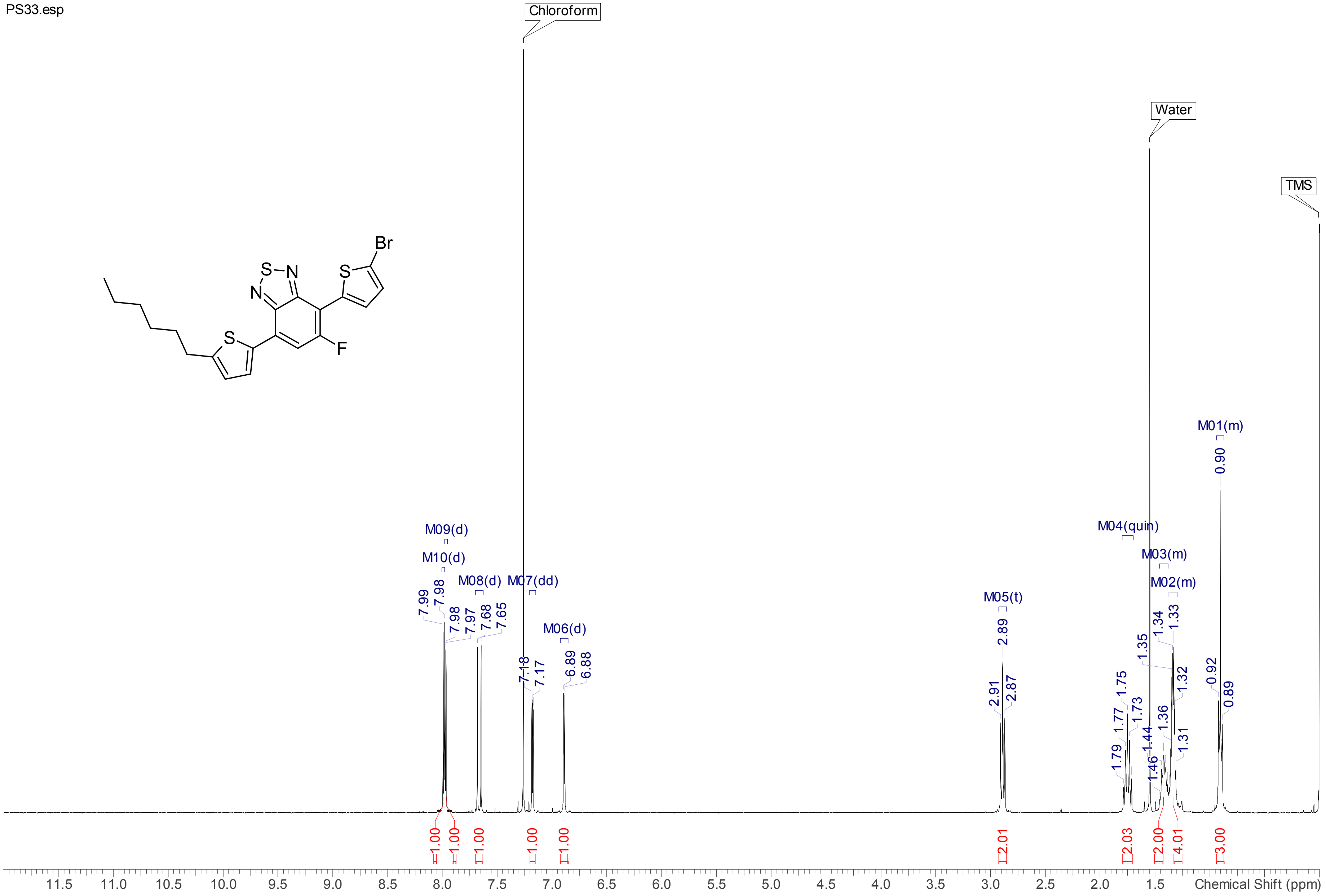

Figure S5. Local zoom of assigned ^{1}H NMR spectrum of 4

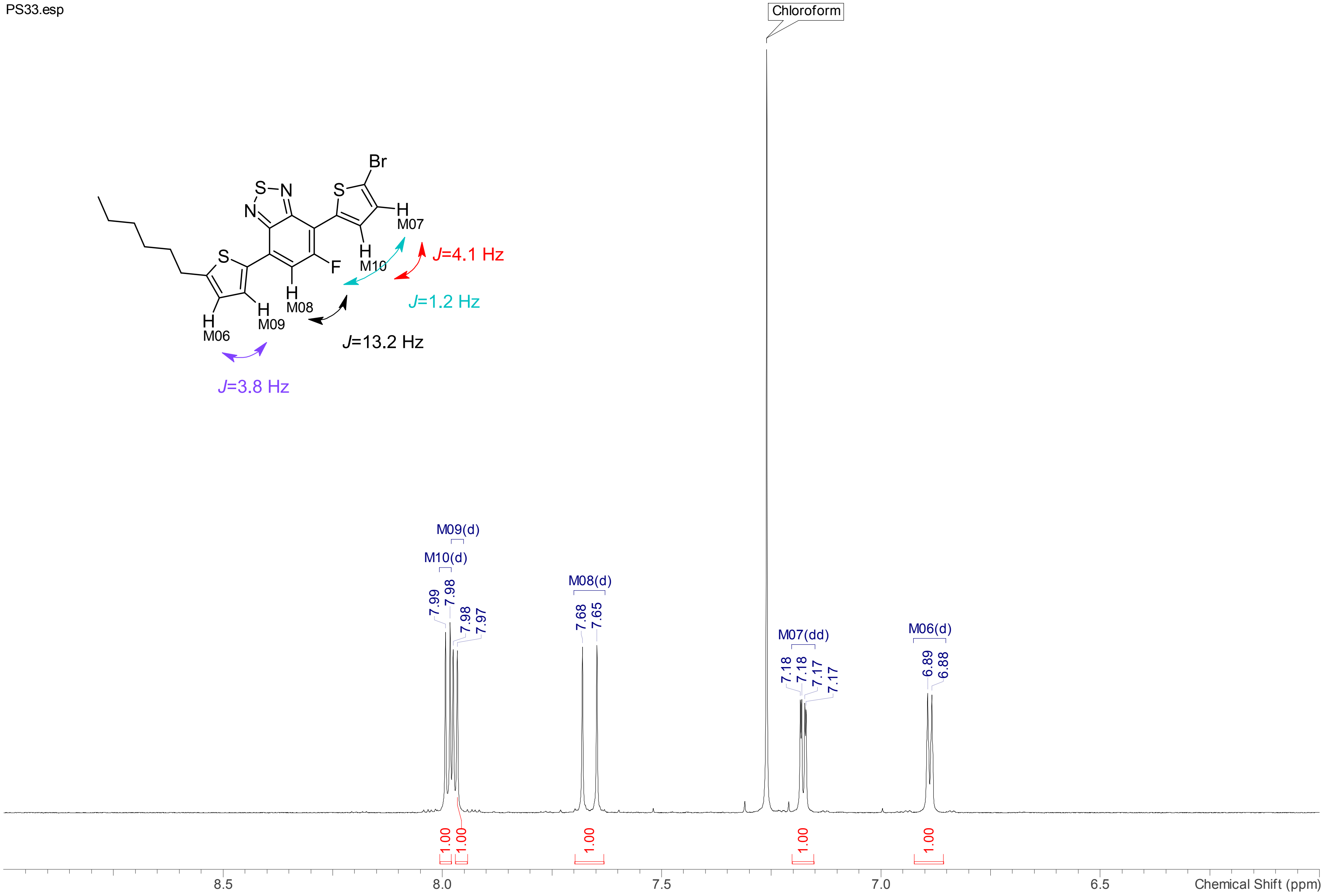

Figure S6. H peak list and assignments of compound 4

No.	Shift1 (ppm)	H's	Type	J (Hz)	Multiplet1	(ppm)
1	0.90	3	m	-	M01	[0.87 .. 0.94]
2	1.34	4	m	-	M02	[1.30 .. 1.37]
3	1.42	2	m	-	M03	[1.38 .. 1.46]
4	1.75	2	quin	7.55	M04	[1.70 .. 1.80]
5	2.89	2	t	7.58	M05	[2.85 .. 2.93]
6	6.89	1	d	3.79	M06	[6.85 .. 6.93]
7	7.18	1	dd	4.10, 1.16	M07	[7.15 .. 7.20]
8	7.66	1	d	13.20	M08	[7.63 .. 7.70]
9	7.97	1	d	3.79	M09	[7.95 .. 7.98]
10	7.99	1	d	4.03	M10	[7.98 .. 8.01]

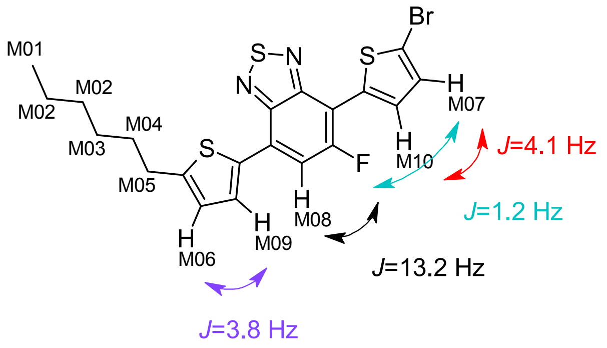

Figure S7. ^1H-^1H 2D NOE NMR of compound 4 in $CDCl_3$

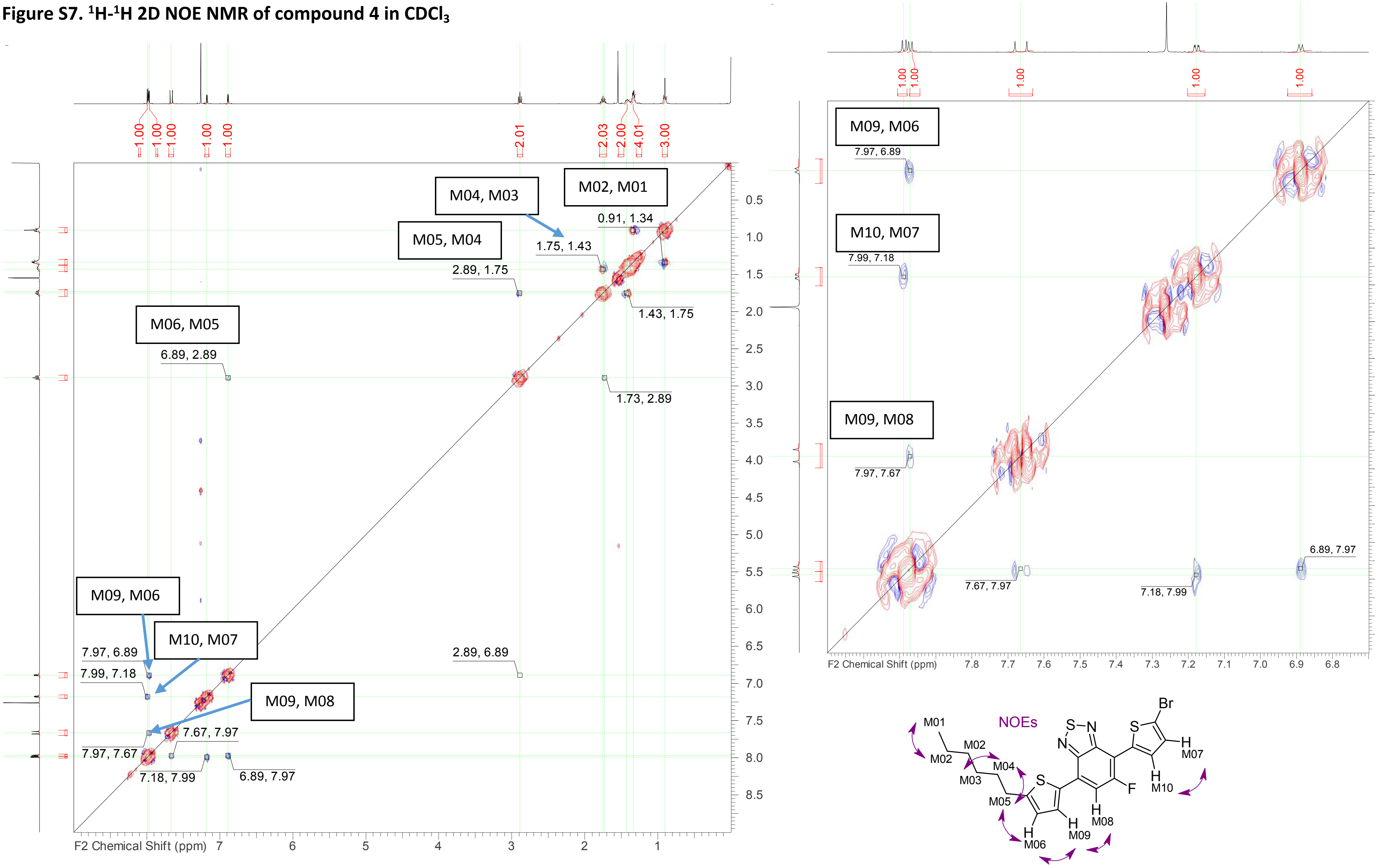

Figure S8. ^{1}H NMR spectrum of 5 in $CDCl_3$

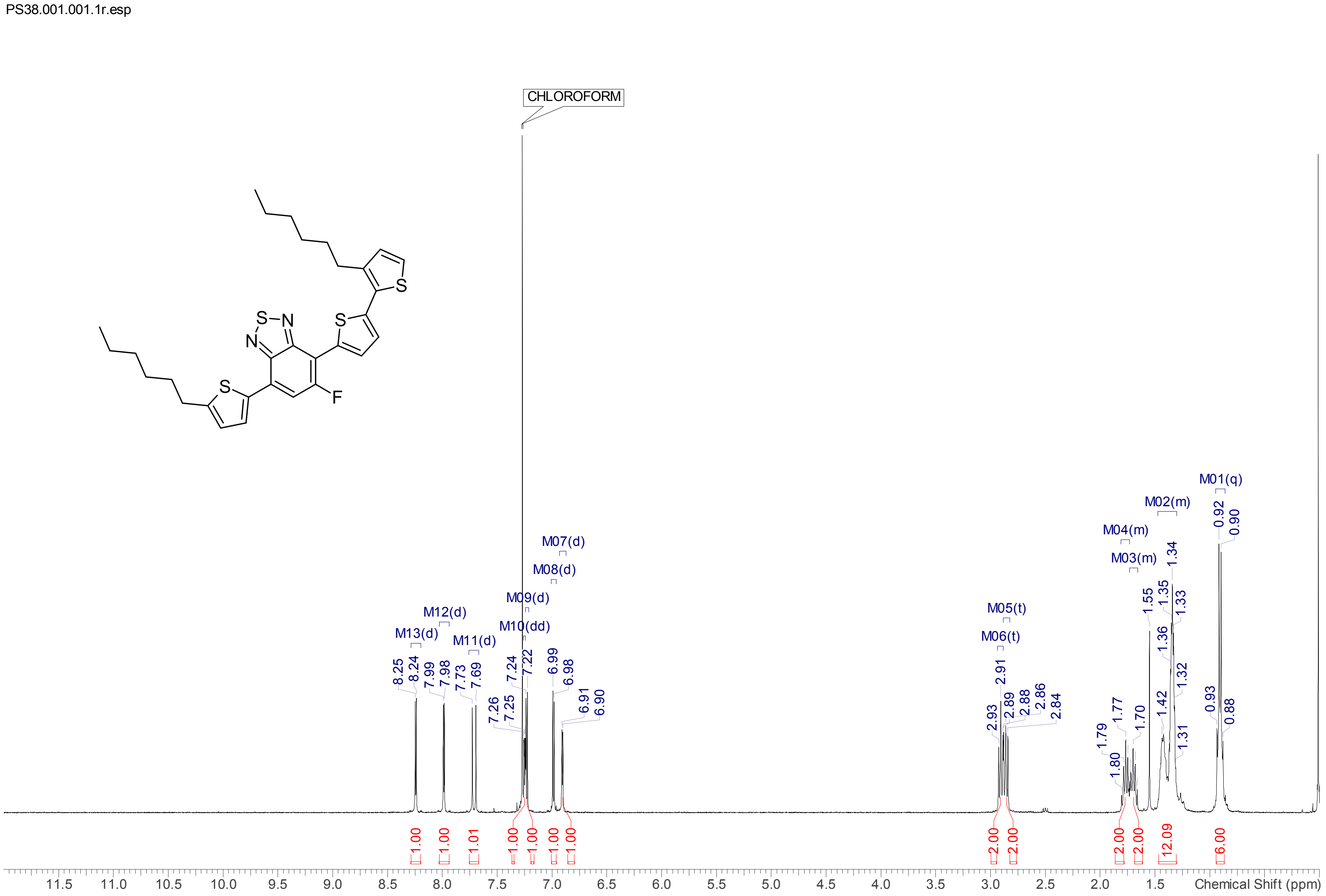

Figure S9. Local zoom of assigned ^{1}H NMR spectrum of 5

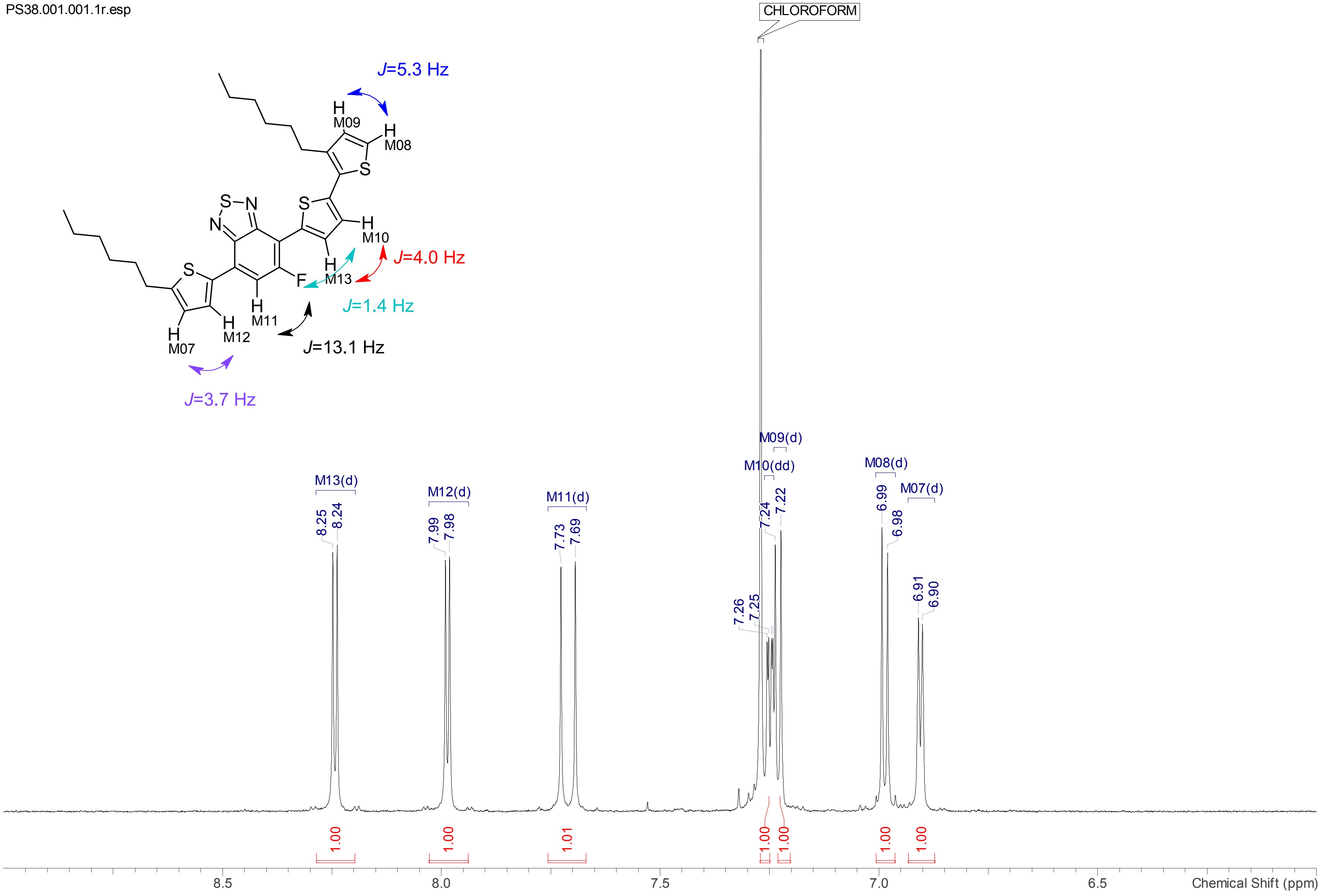

Figure S10. H peak list and assignments of compound 5

No.	Shift1 (ppm)	H's	Type	J (Hz)	Multiplet1	(ppm)
1	0.91	6	q	7.09	M01	[0.86 .. 0.95]
2	1.36	12	m	-	M02	[1.30 .. 1.47]
3	1.70	2	m	-	M03	[1.66 .. 1.73]
4	1.77	2	m	-	M04	[1.73 .. 1.81]
5	2.86	2	t	7.80	M05	[2.82 .. 2.88]
6	2.91	2	t	7.64	M06	[2.88 .. 2.94]
7	6.91	1	d	3.67	M07	[6.87 .. 6.93]

No.	Shift1 (ppm)	H's	Type	J (Hz)	Multiplet1	(ppm)
8	6.99	1	d	5.26	M08	[6.96 .. 7.01]
9	7.23	1	d	5.26	M09	[7.21 .. 7.24]
10	7.25	1	dd	3.97, 1.41	M10	[7.24 .. 7.26]
11	7.71	1	d	13.08	M11	[7.67 .. 7.76]
12	7.99	1	d	3.67	M12	[7.94 .. 8.03]
13	8.24	1	d	4.04	M13	[8.20 .. 8.29]

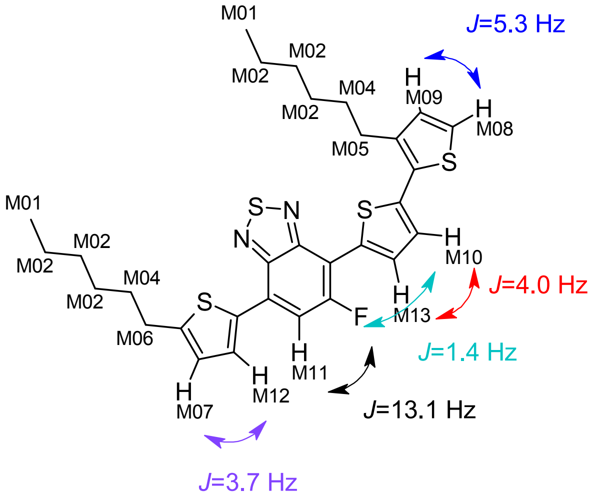

Figure S11. ^{1}H **NMR spectrum of 6 in** $CDCl_3$

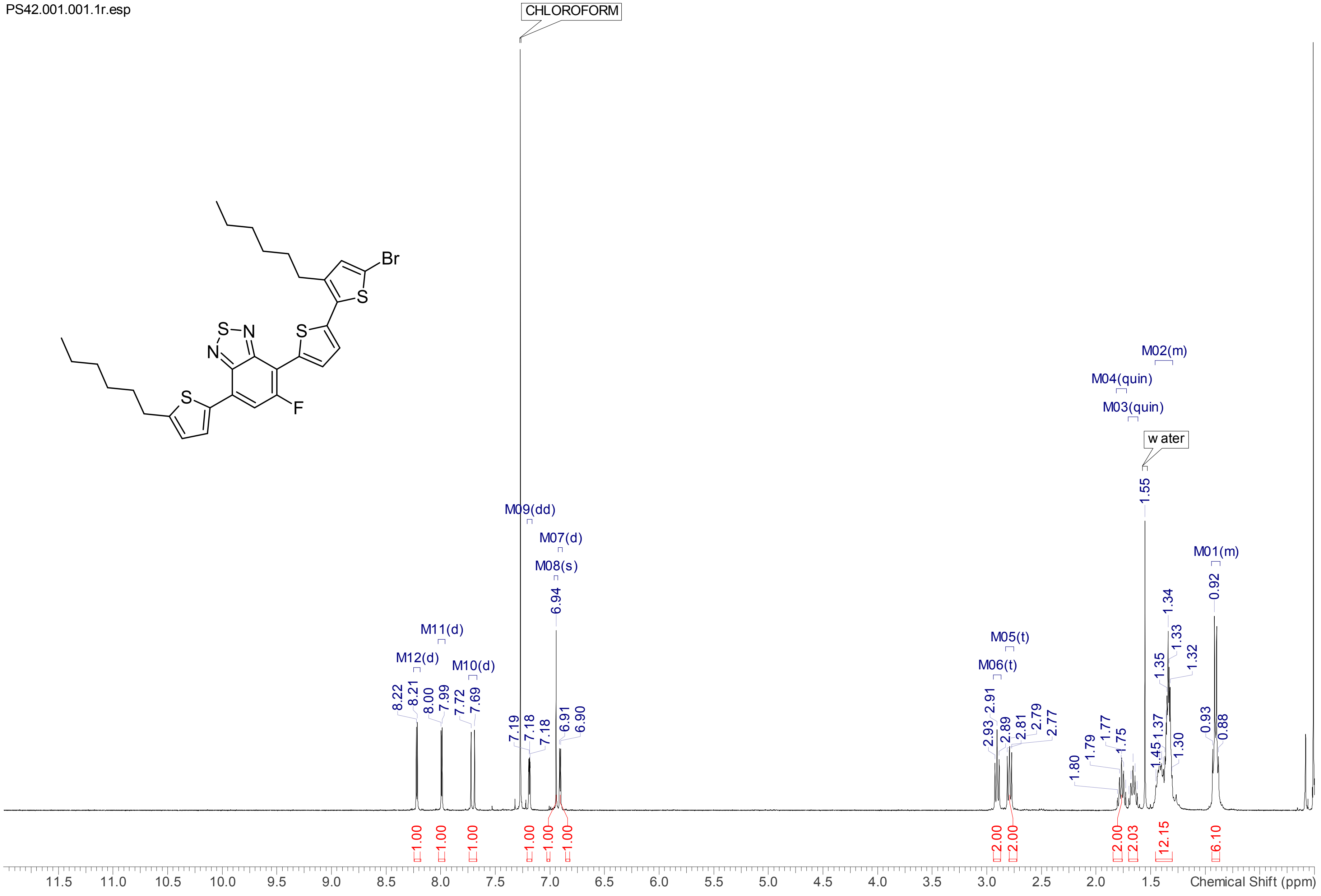

Figure S12. Local zoom of assigned ^{1}H NMR spectrum of 6

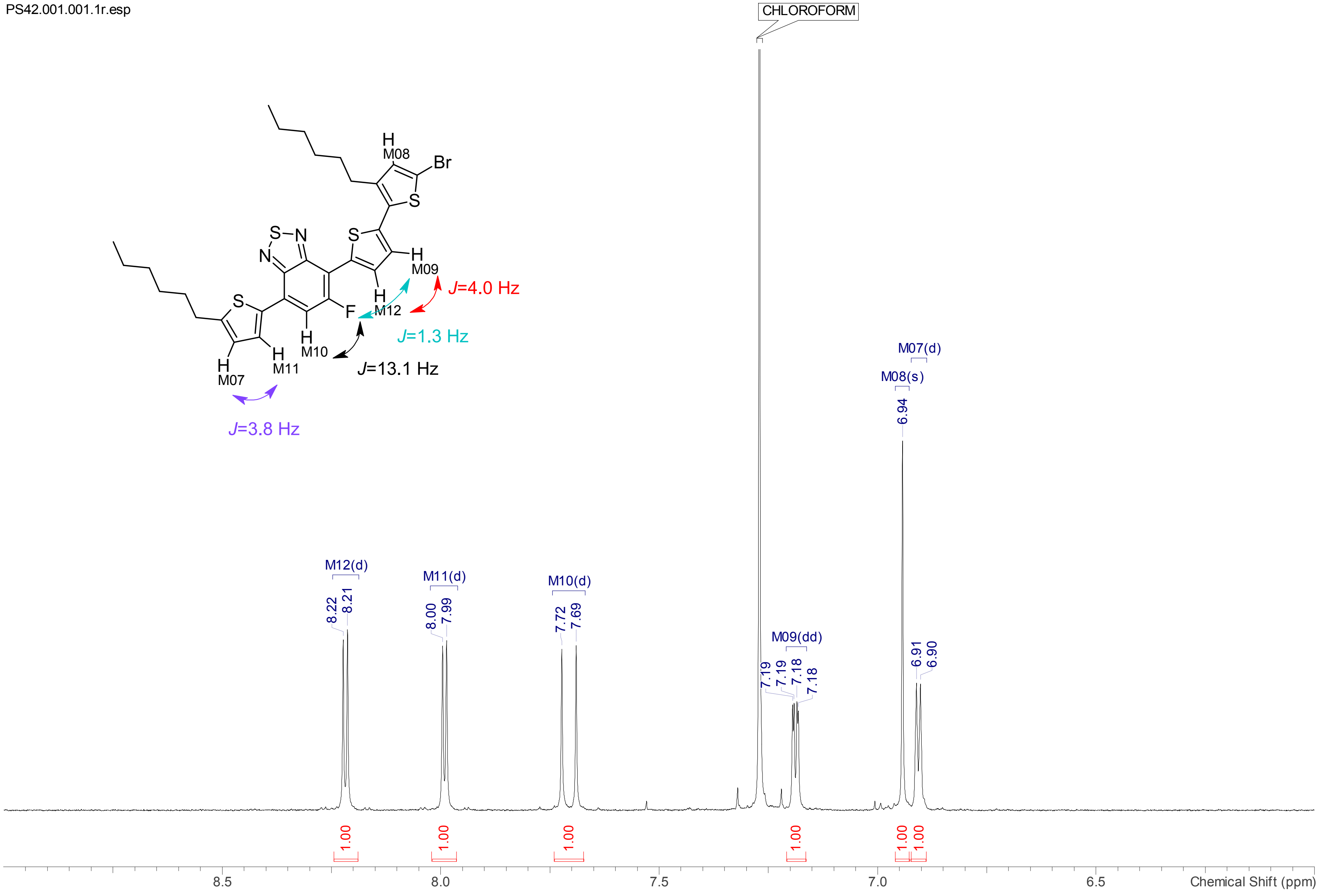

Figure S13. H peak list and assignments of compound 6

No.	Shift1 (ppm)	H's	Type	J (Hz)	Multiplet1	(ppm)
1	0.91	6	m	-	M01	[0.86 .. 0.94]
2	1.35	12	m	-	M02	[1.30 .. 1.46]
3	1.66	2	quin	7.61	M03	[1.62 .. 1.71]
4	1.77	2	quin	7.52	M04	[1.72 .. 1.81]
5	2.79	2	t	7.80	M05	[2.75 .. 2.83]
6	2.91	2	t	7.64	M06	[2.87 .. 2.94]

No.	Shift1 (ppm)	H's	Type	J (Hz)	Multiplet1	(ppm)
7	6.91	1	d	3.67	M07	[6.89 .. 6.92]
8	6.94	1	s	-	M08	[6.93 .. 6.96]
9	7.19	1	dd	3.97, 1.28	M09	[7.16 .. 7.21]
10	7.71	1	d	13.08	M10	[7.67 .. 7.74]
11	7.99	1	d	3.79	M11	[7.96 .. 8.02]
12	8.22	1	d	3.91	M12	[8.19 .. 8.25]

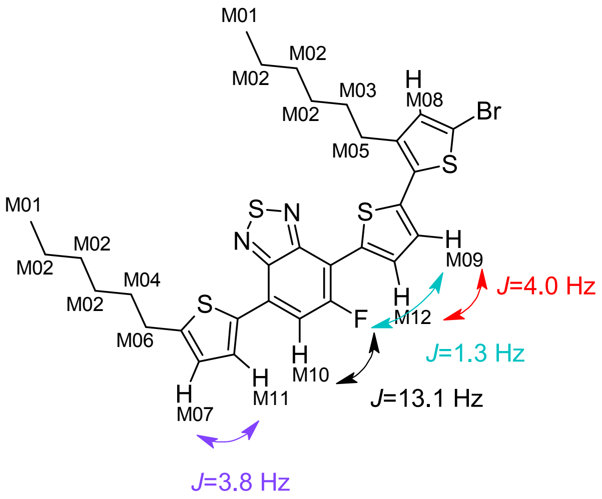

Figure S14. ^{1}H-^{1}H 2D NOE NMR of compound 6 in $CDCl_3$

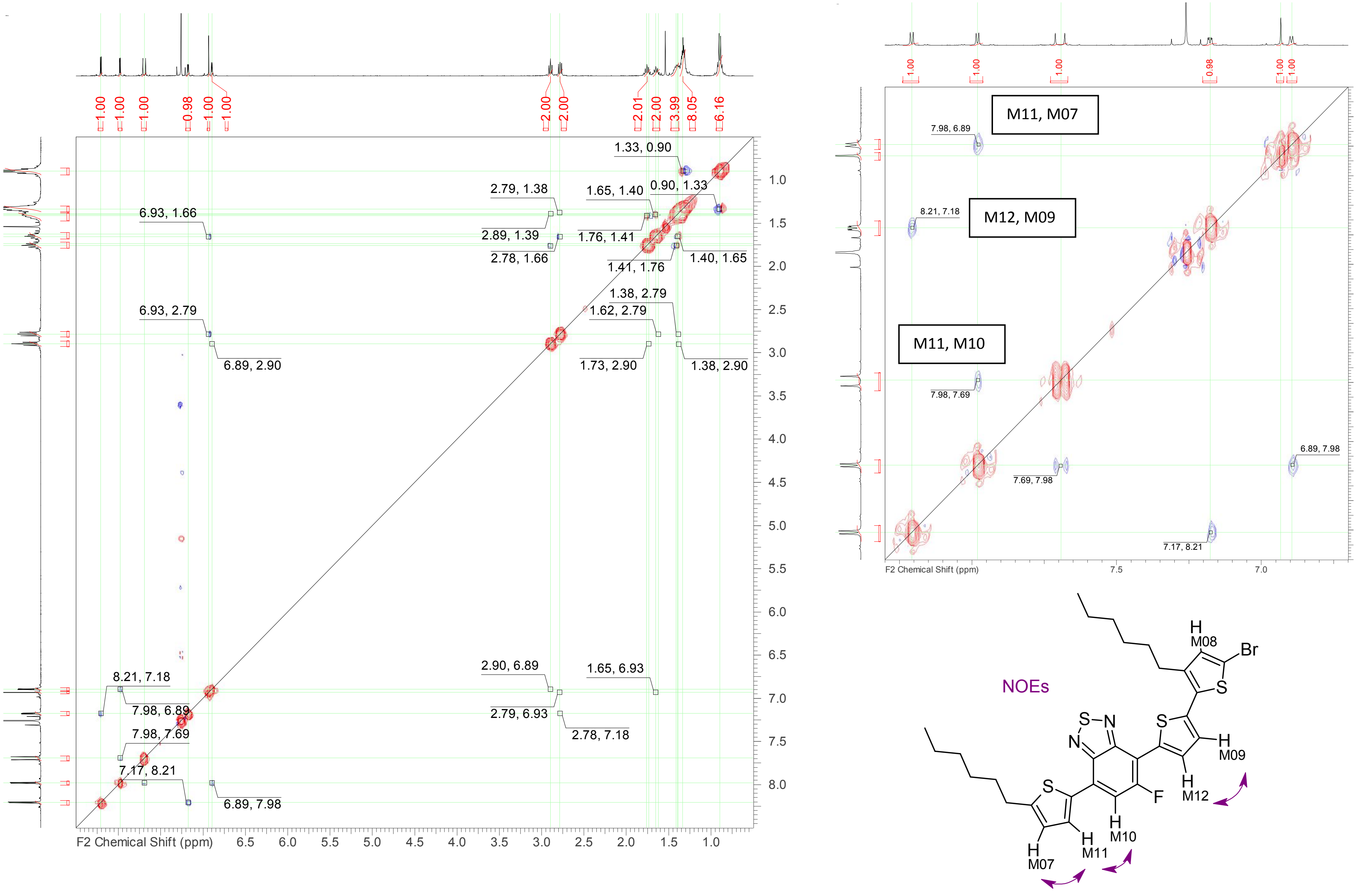

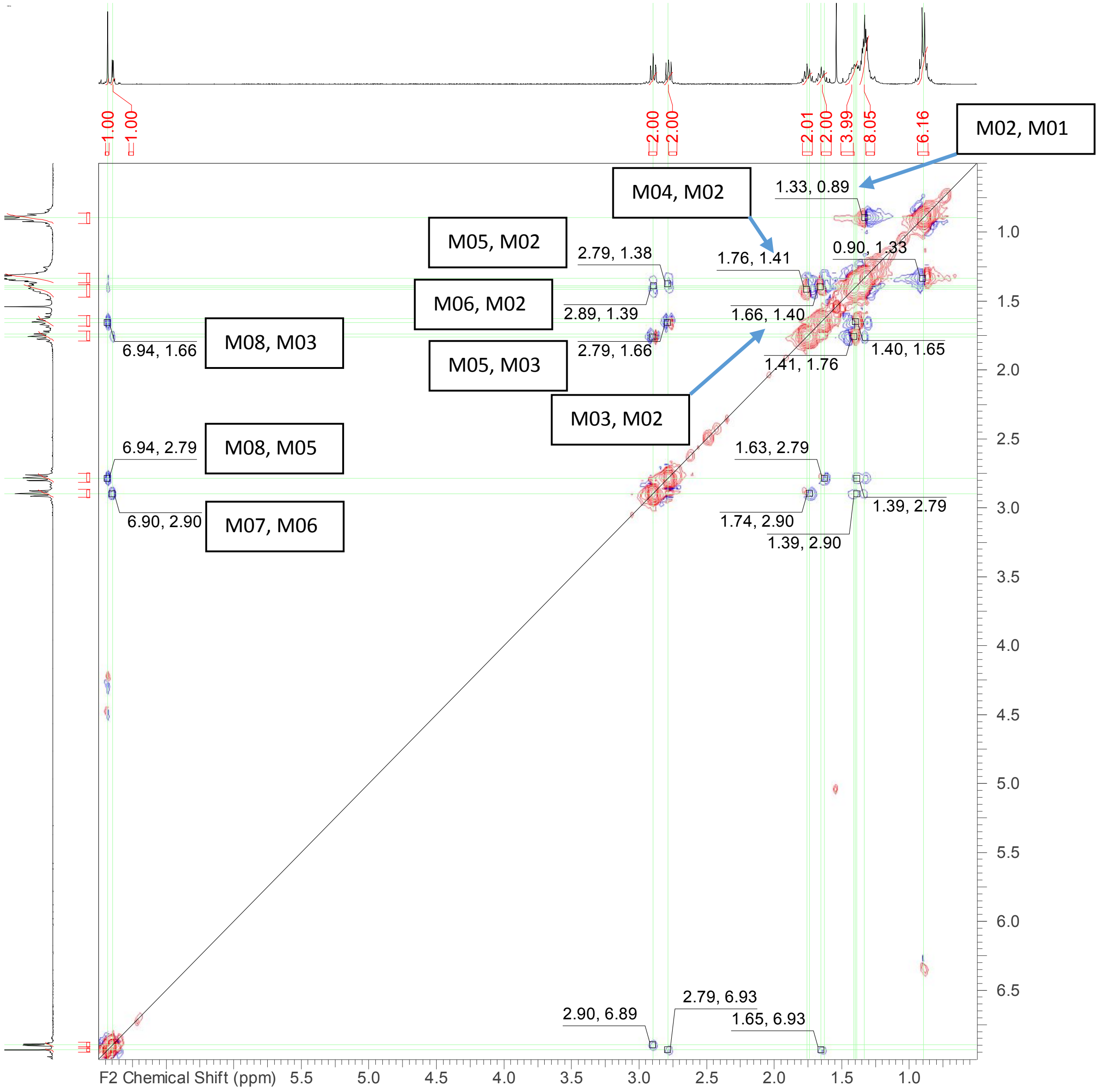
M02, M01
M04, M02
M05, M02
M06, M02
M08, M03
M05, M03
M03, M02
M08, M05
M07, M06
1.33, 0.89
0.90, 1.33
2.79, 1.38
1.76, 1.41
2.89, 1.39
1.66, 1.40
6.94, 1.66
2.79, 1.66
1.40, 1.65
1.41, 1.76
6.94, 2.79
1.63, 2.79
6.90, 2.90
1.74, 2.90
1.39, 2.79
1.39, 2.90
2.90, 6.89
2.79, 6.93
1.65, 6.93
1.00
1.00
2.00
2.00
2.01
2.00
3.99
8.05
6.16
F2 Chemical Shift (ppm)

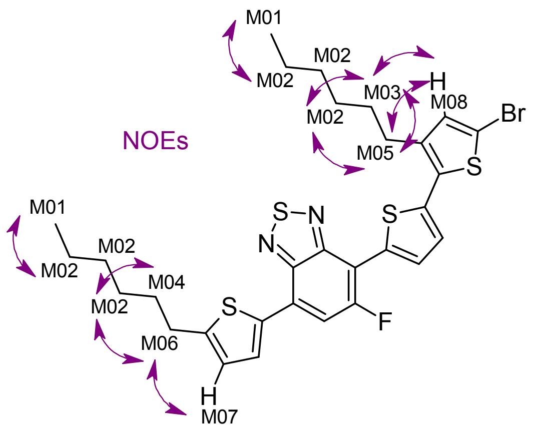
NOEs
M01
M02
M02
M03
H
M08
Br
M02
M05
S
S
S—N
N
M01
M02
M02
M04
M02
M06
S
F
H
M07

Figure S15. ^{1}H NMR spectrum of 7 in $CDCl_3$

J630.002.001.1r.esp

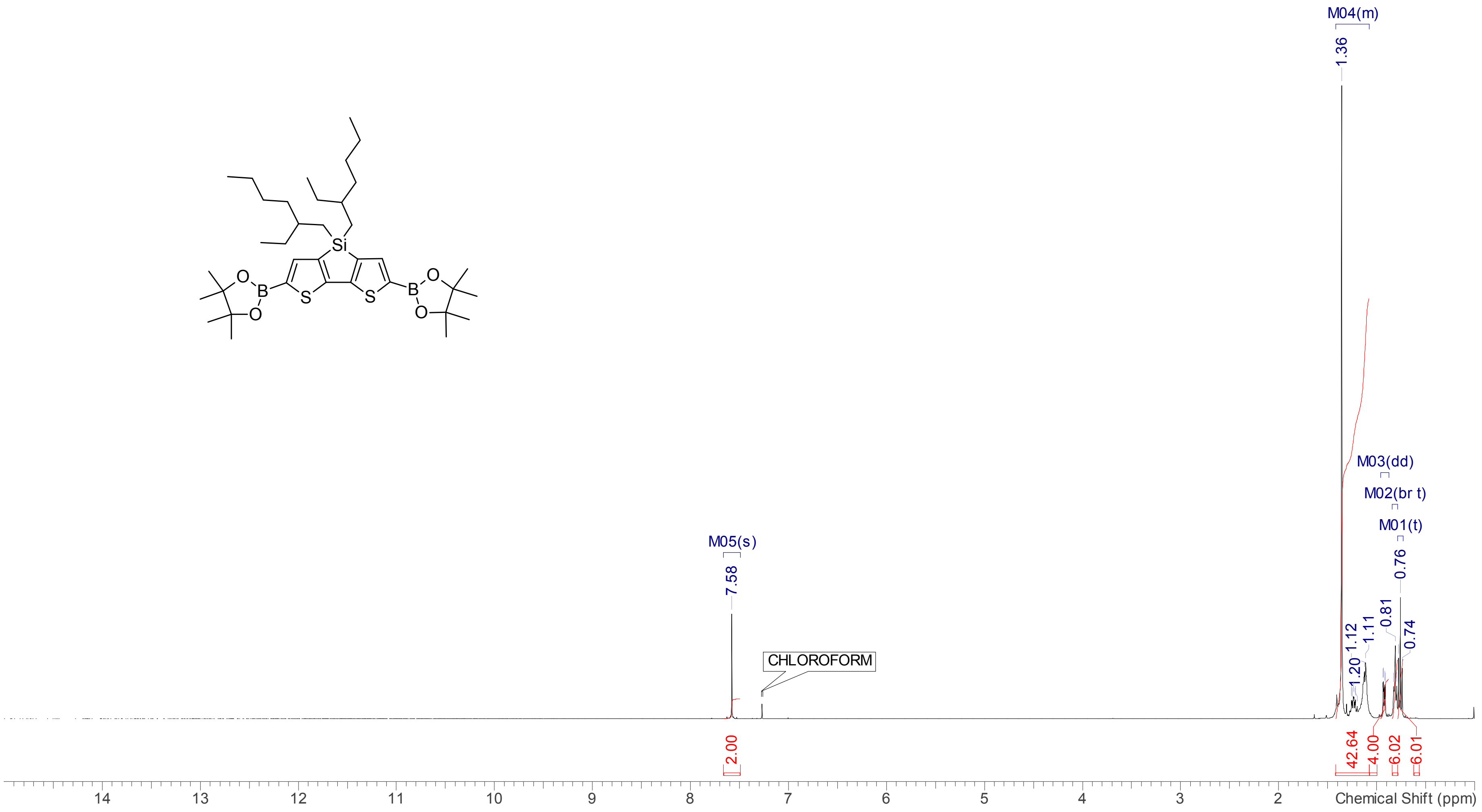

Figure S16. ^{1}H NMR spectrum of 10 in $CDCl_3$

J601.002.001.1r.esp

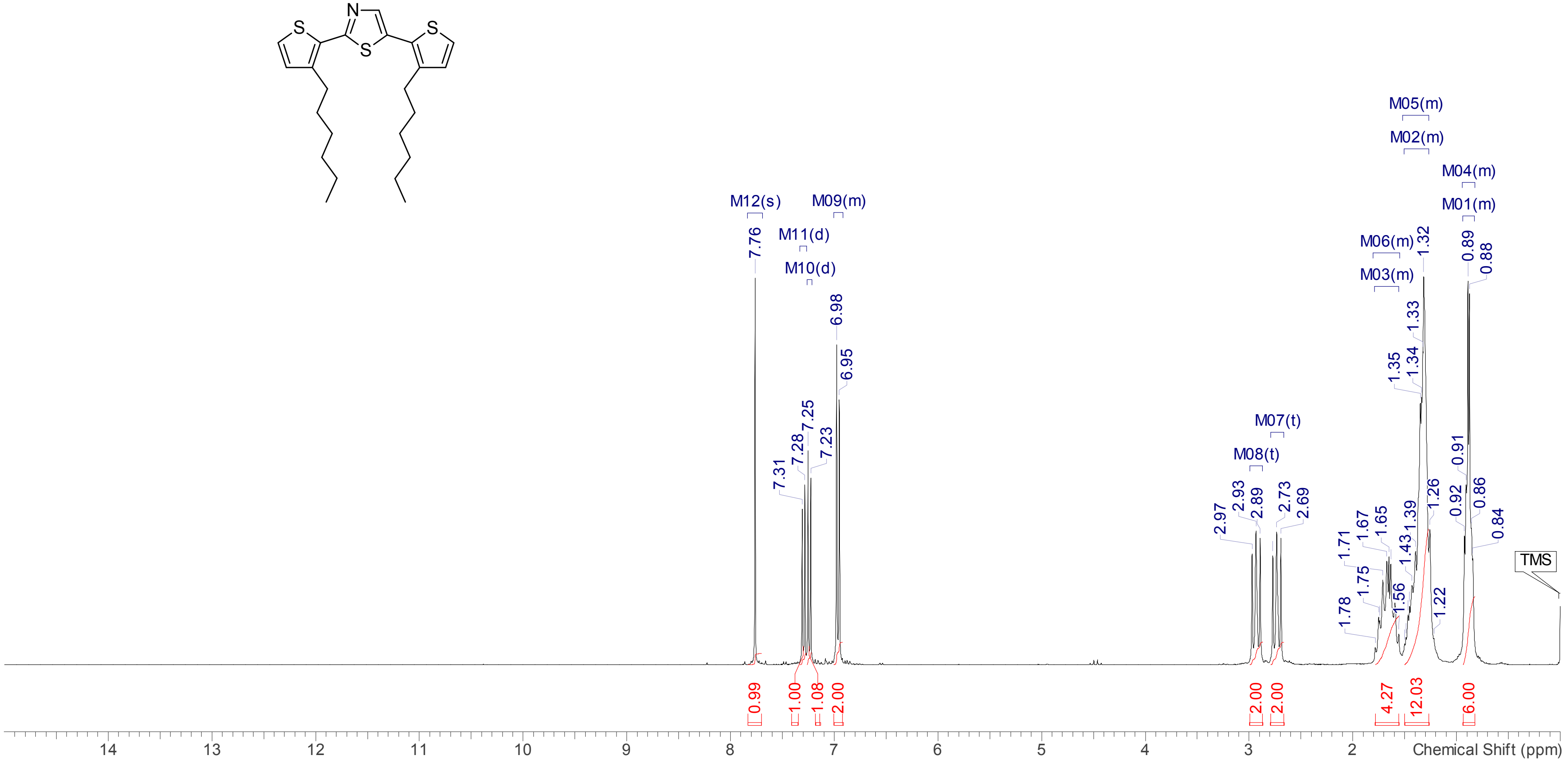

Figure S17. Local zoom of assigned ^{1}H NMR spectrum of 10

J601.002.001.1r.esp

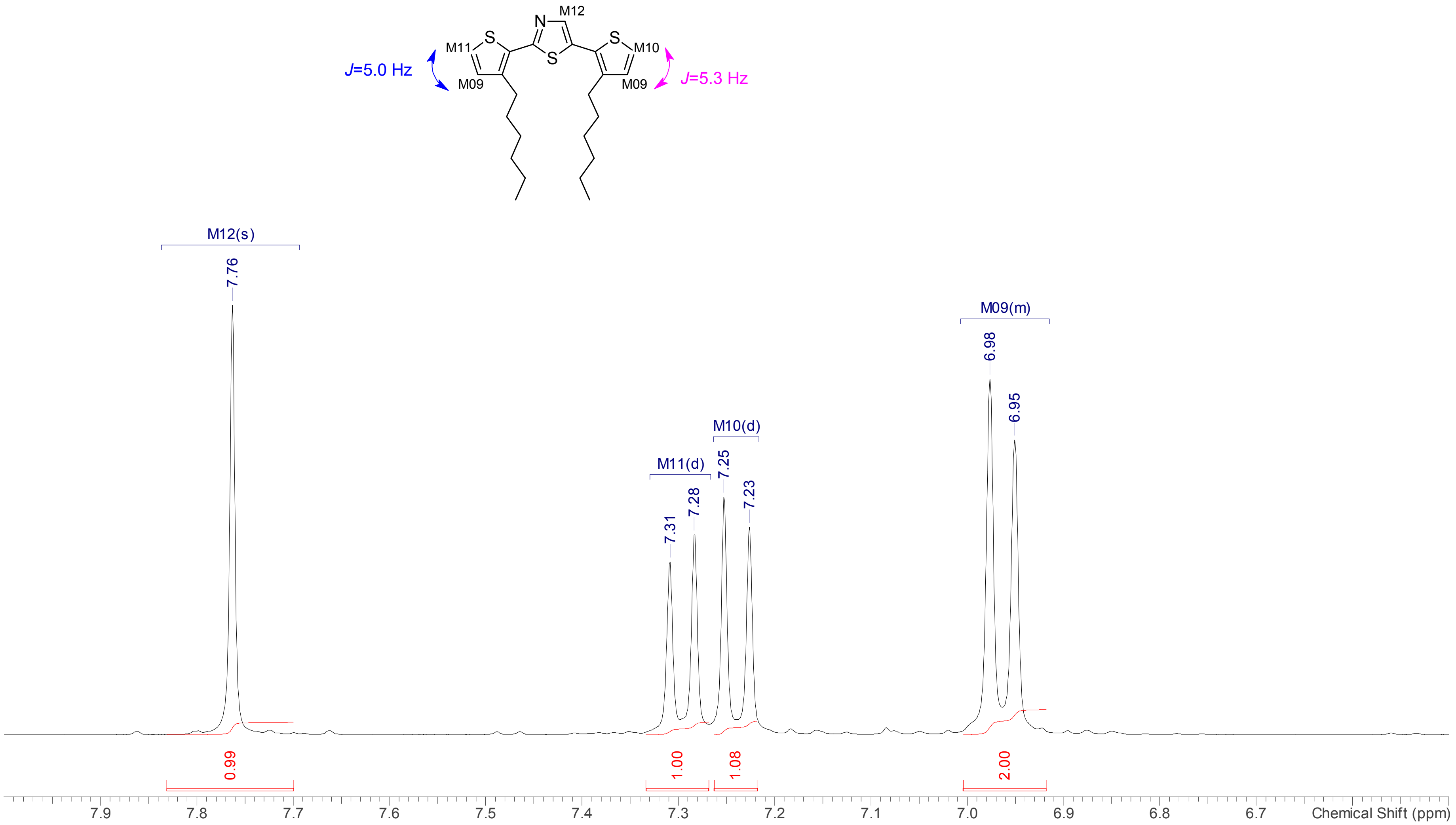

Figure S18. ^{13}**C NMR spectrum of 10 in CDCl$_3$**

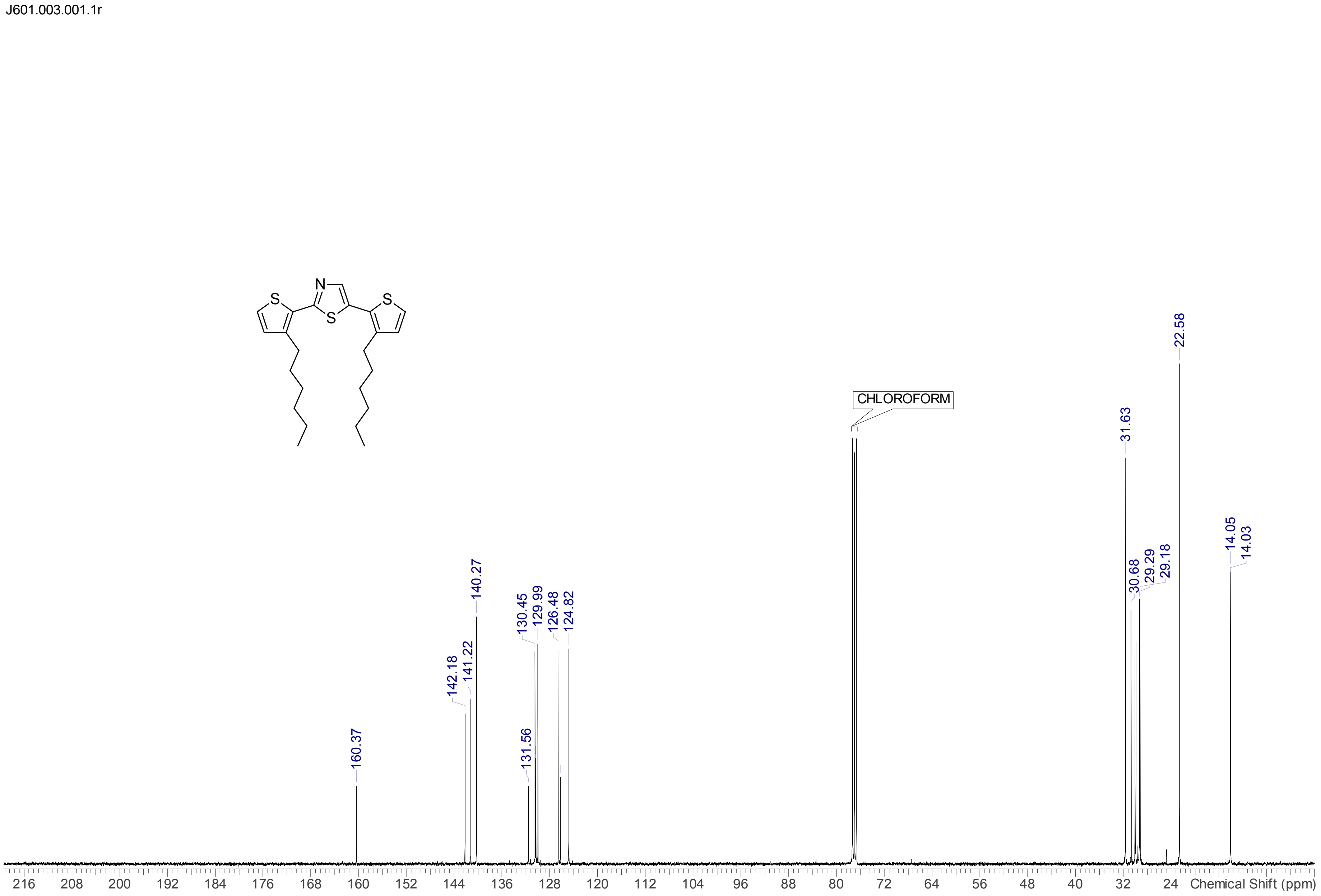

Table S1. Peak list of ^{13}C NMR spectrum of 10 in $CDCl_3$

No.	(ppm)	(Hz)	Height
1	14.03	1411.9	0.5747
2	14.05	1413.3	0.5931
3	22.58	2271.6	1.0000
4	29.18	2936.3	0.5388
5	29.29	2946.5	0.5291
6	29.31	2948.7	0.4975

No.	(ppm)	(Hz)	Height
7	29.91	3008.9	0.4447
8	30.03	3021.4	0.4182
9	30.68	3086.6	0.5084
10	31.63	3182.0	0.8123
11	124.82	12558.1	0.4299
12	126.23	12700.4	0.1734

No.	(ppm)	(Hz)	Height
13	126.48	12725.4	0.4291
14	129.99	13078.2	0.4406
15	130.33	13112.7	0.2111
16	130.45	13125.2	0.4250
17	131.56	13236.7	0.1556
18	140.27	14112.6	0.4947

No.	(ppm)	(Hz)	Height
19	141.22	14208.7	0.3298
20	142.18	14305.5	0.3002
21	160.37	16135.1	0.1555

Figure S19. ^{1}H **NMR spectrum of 11a in** $CDCl_3$

J602.005.001.1r.esp

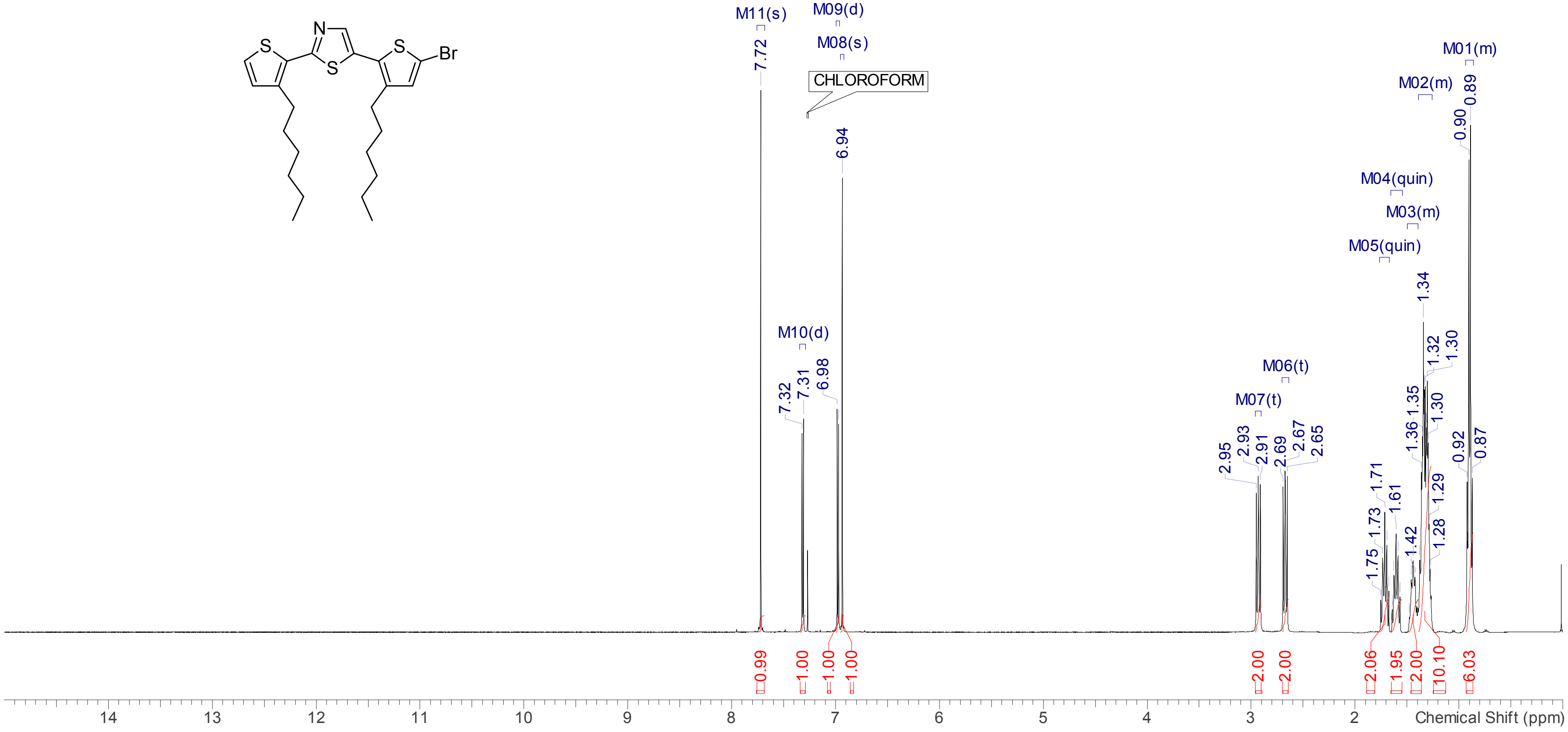

Figure S20. Local zoom of assigned ^{1}H NMR spectrum of 11a

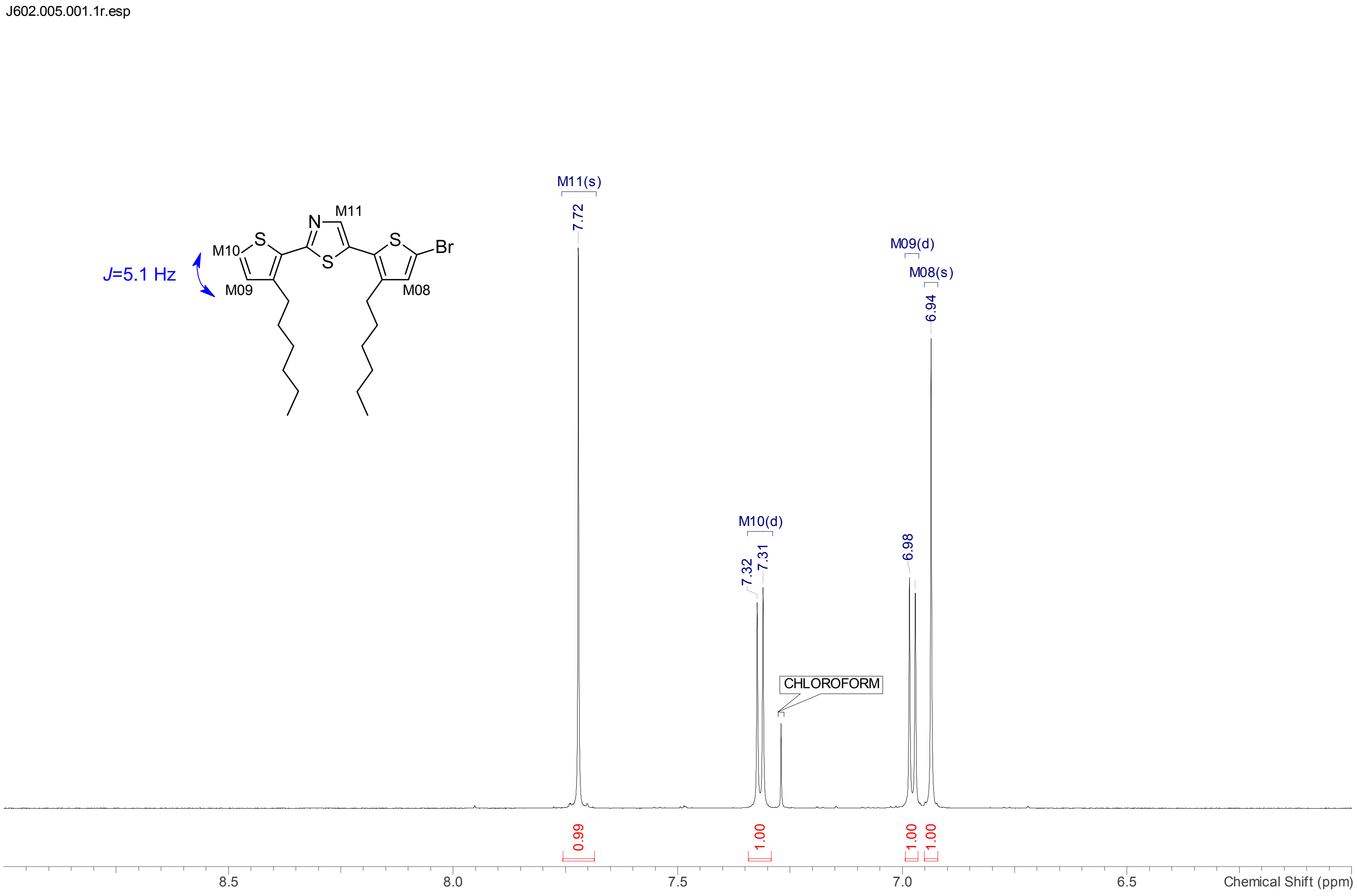

Figure S21. Local zoom of assigned ^{1}H NMR spectrum of 11a

J602.005.001.1r.esp

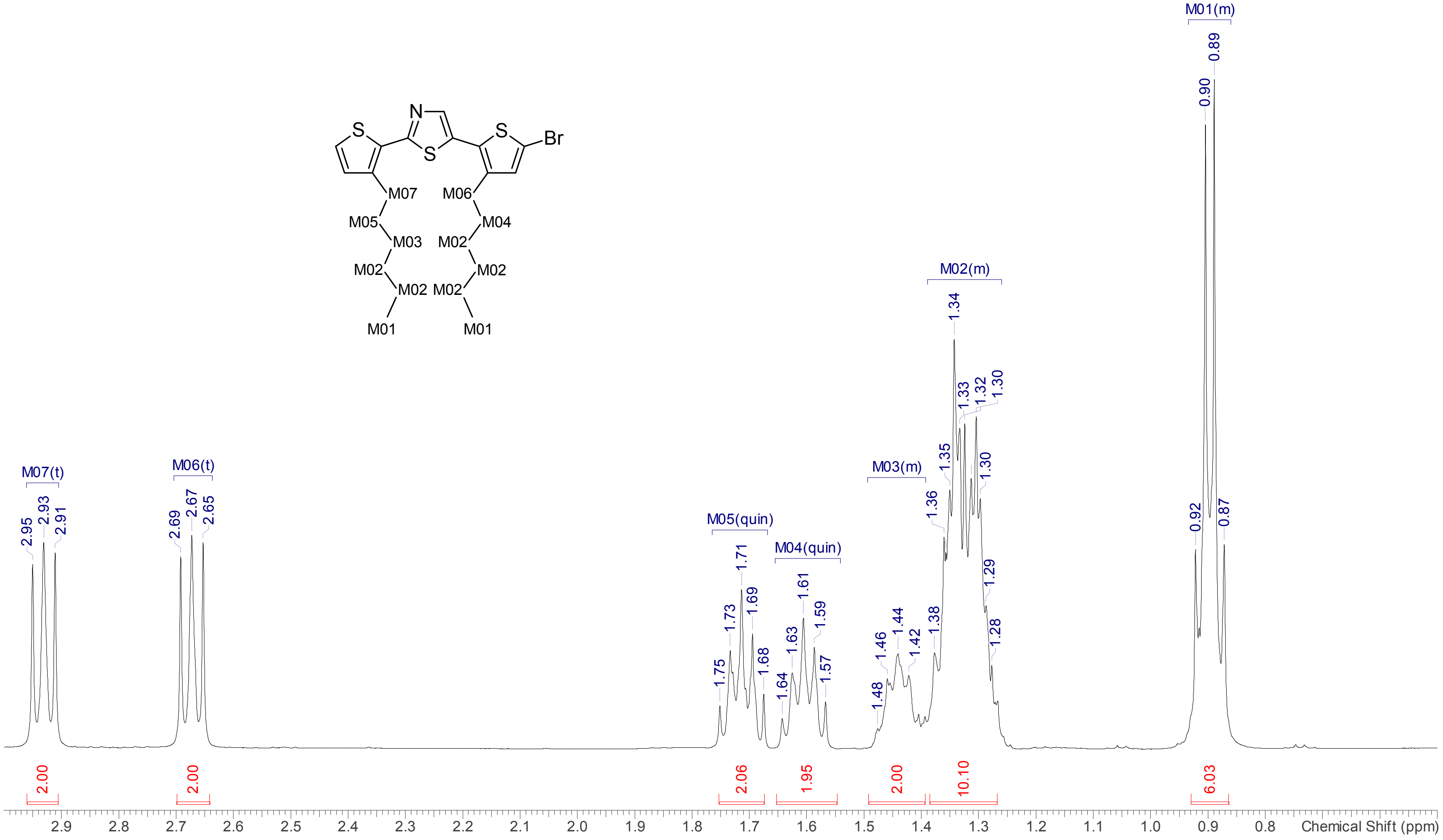

Figure S22. ^{13}C **NMR spectrum of 11a in** $CDCl_3$

J602.004.001.1r.esp

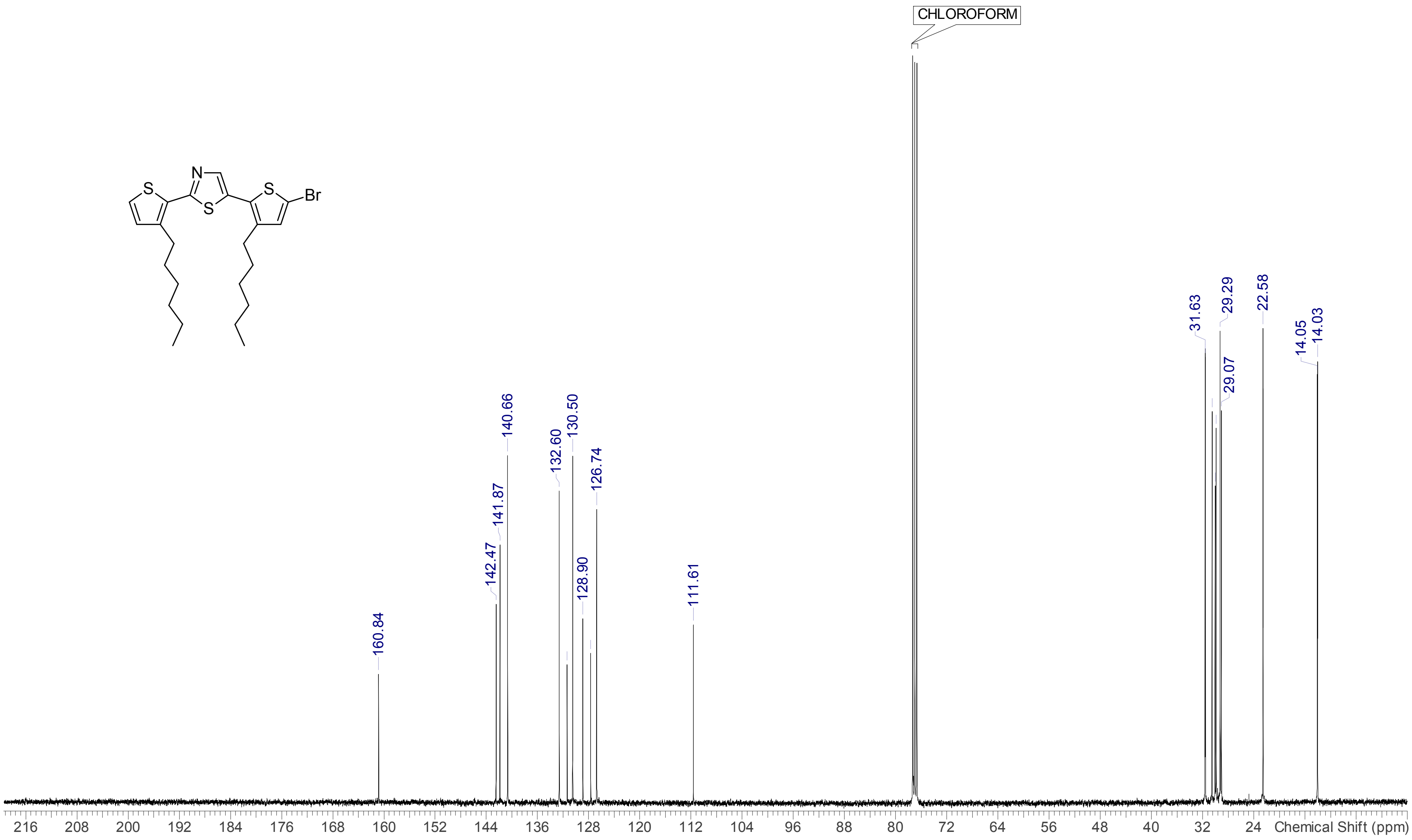

Table S2. Peak list of ^{13}C NMR spectrum of 11a in $CDCl_3$

No.	(ppm)	(Hz)	Height
1	14.03	1411.1	0.5877
2	14.05	1414.1	0.5704
3	22.55	2268.7	0.5508
4	22.58	2271.6	0.6321
5	29.07	2925.3	0.5218
6	29.26	2944.3	0.4394
7	29.29	2946.5	0.6289
8	29.88	3006.0	0.4978
9	30.05	3023.6	0.4204
10	30.53	3072.0	0.5203
11	31.57	3176.1	0.5934
12	31.63	3182.0	0.6052
13	111.61	11229.6	0.2329
14	126.74	12751.8	0.3884
15	127.67	12844.9	0.1948
16	128.90	12968.9	0.2411
17	130.50	13129.6	0.4605
18	131.35	13215.4	0.1796
19	132.60	13340.9	0.4134
20	140.66	14152.2	0.4612
21	141.87	14274.0	0.3406
22	142.47	14334.1	0.2605
23	160.84	16182.1	0.1663

Figure S23. Local zoom of HSQC spectrum of 11a with assignments

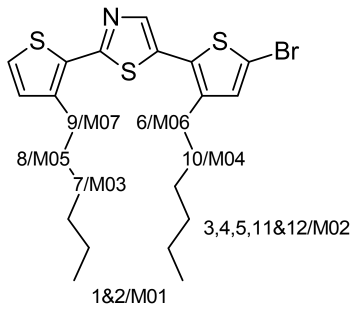

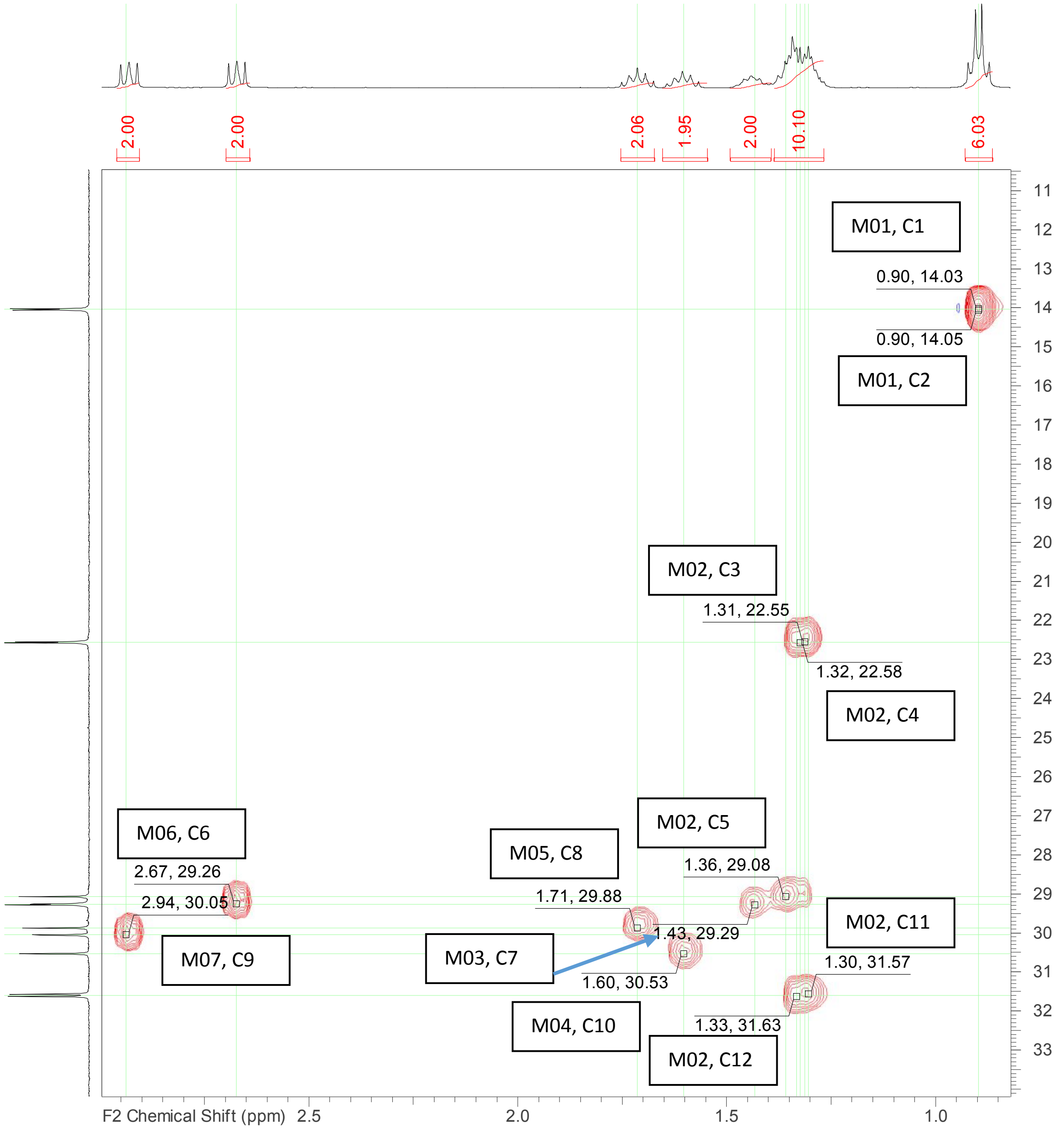

Figure S24. ^{13}C DEPT45 and the local zoom of HSQC spectrum of 11a

J602.010.001.1r.esp

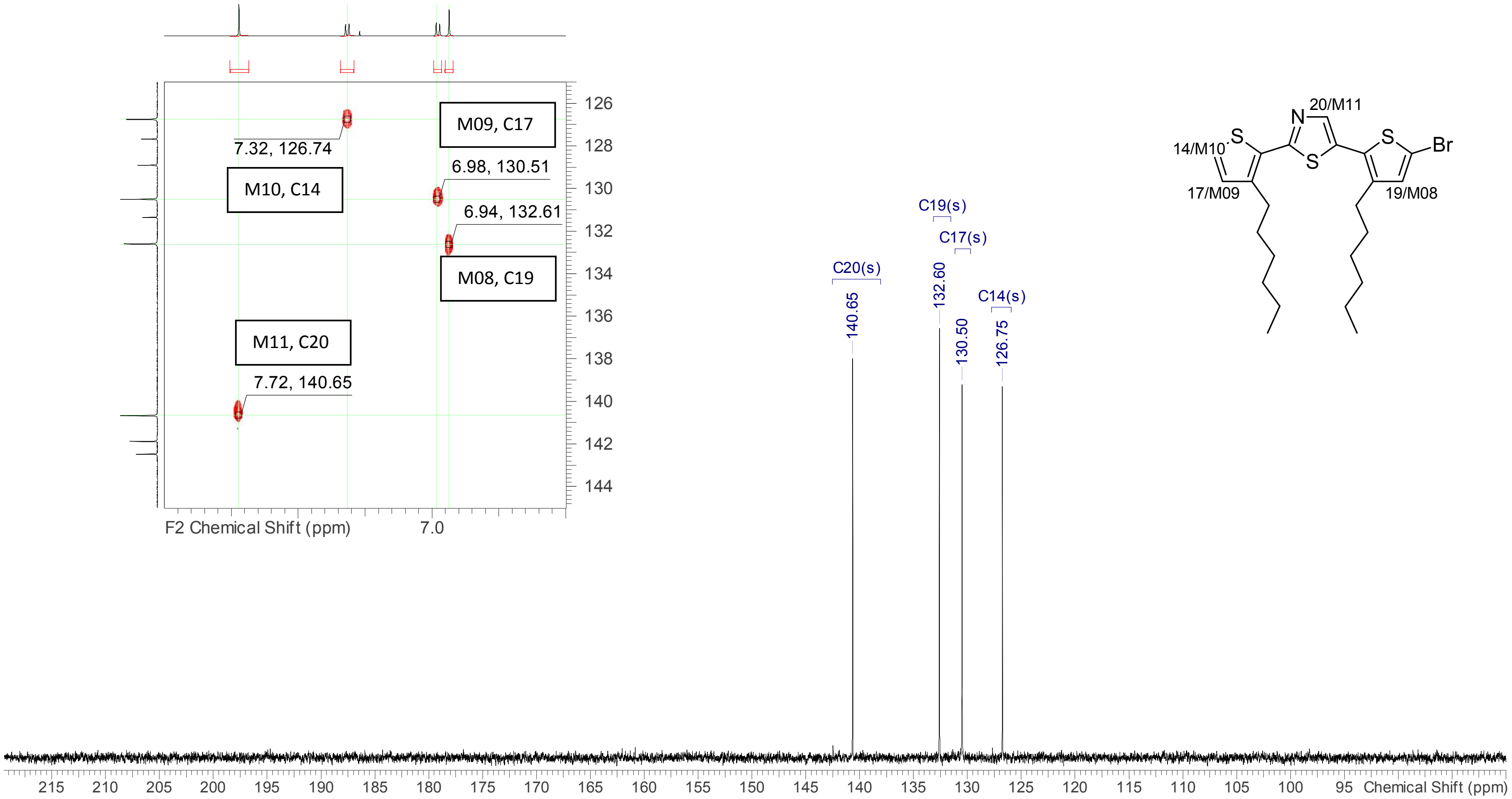

Figure S25. HMBC spectrum of 11a

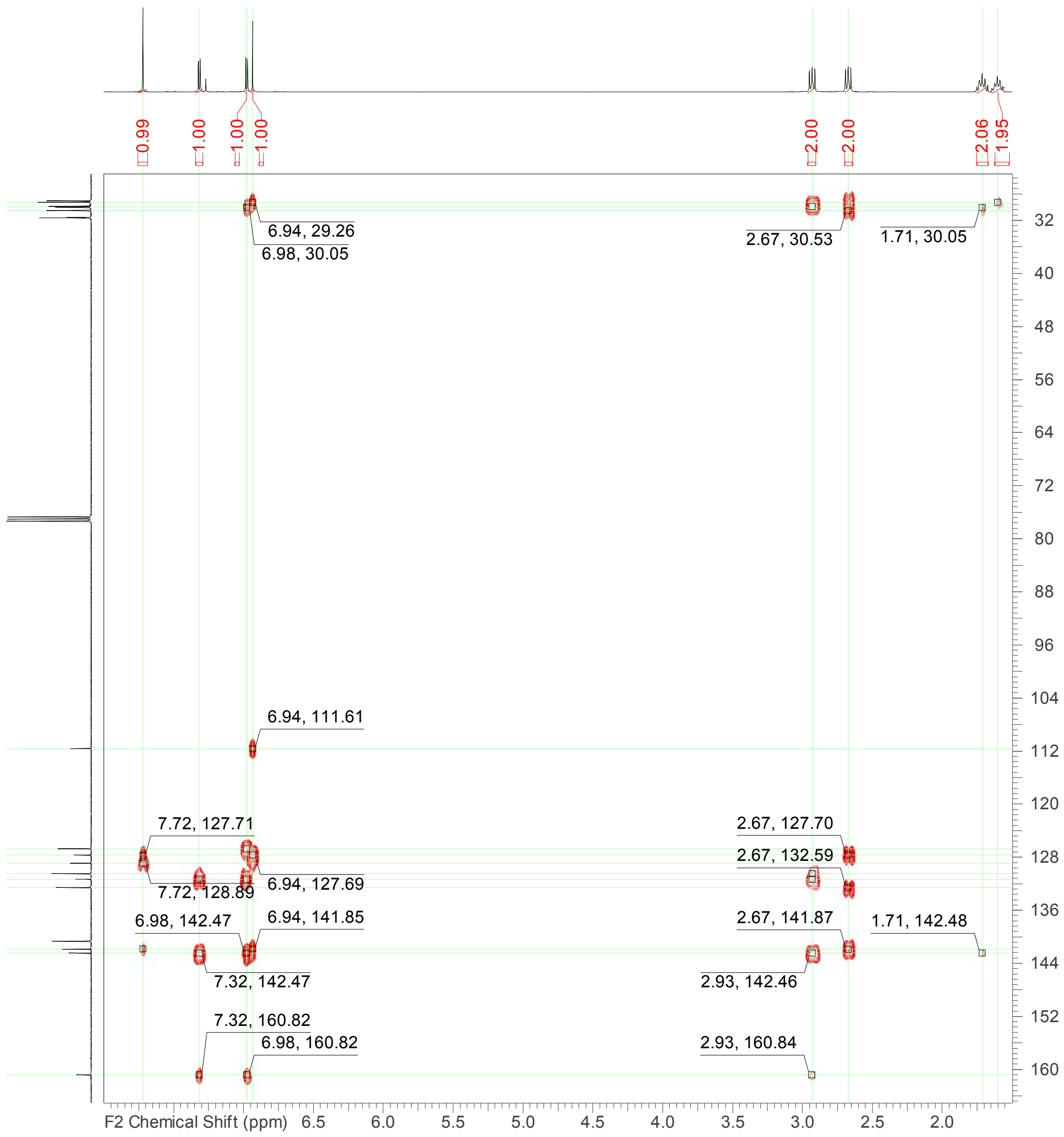

Figure S26. Local zoom of assigned HMBC spectrum of 11a

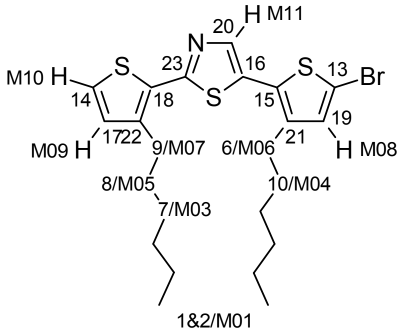

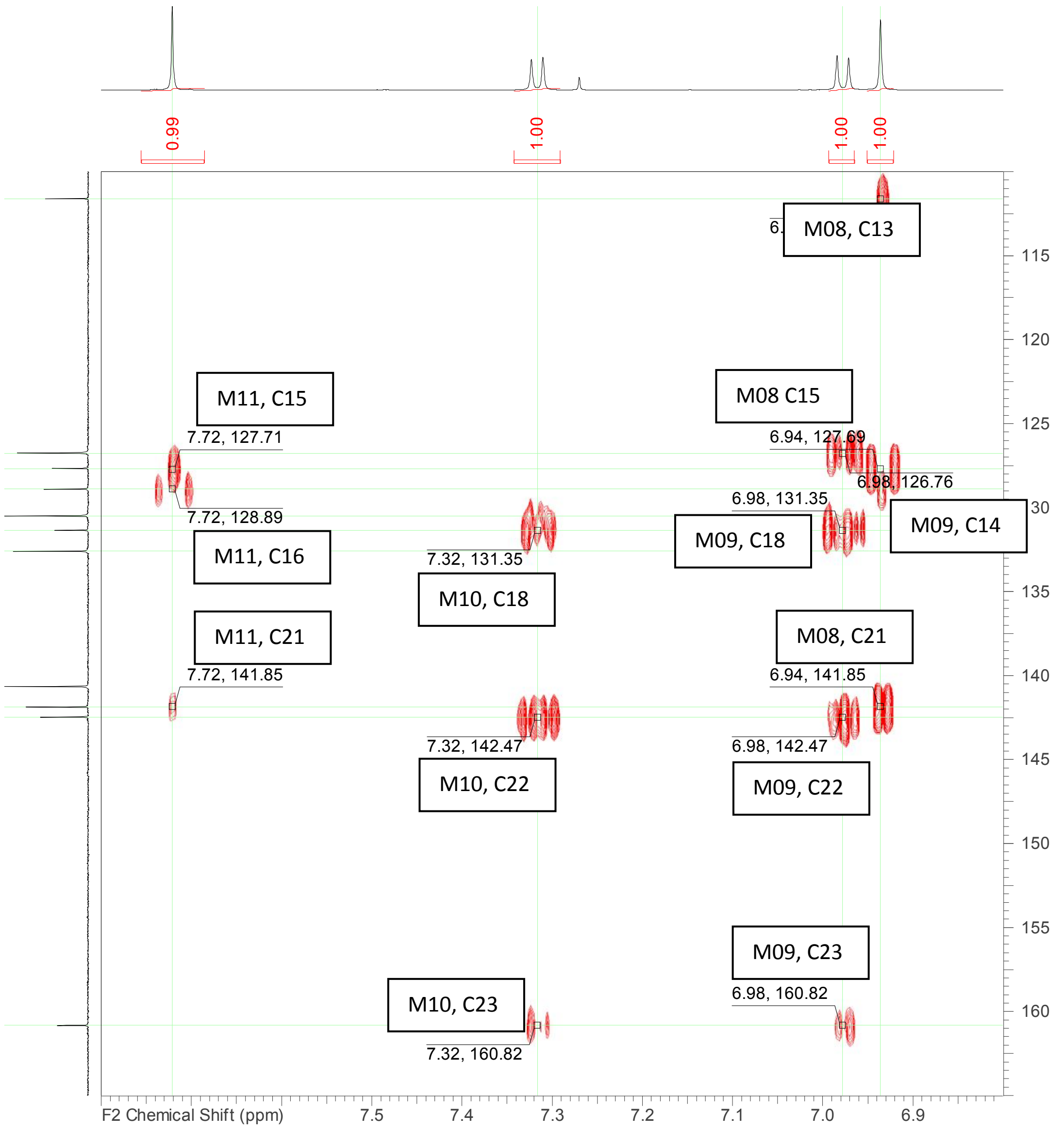

Figure S27. Local zoom of HMBC spectrum of 11a

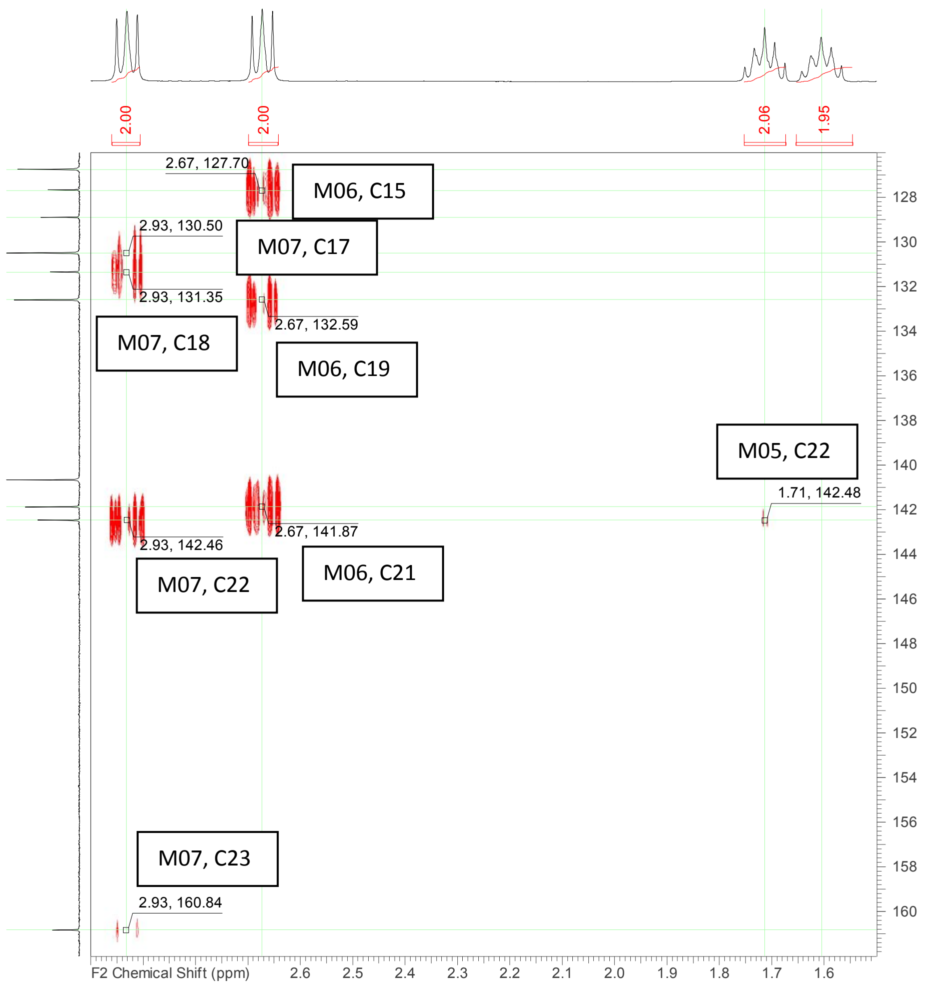

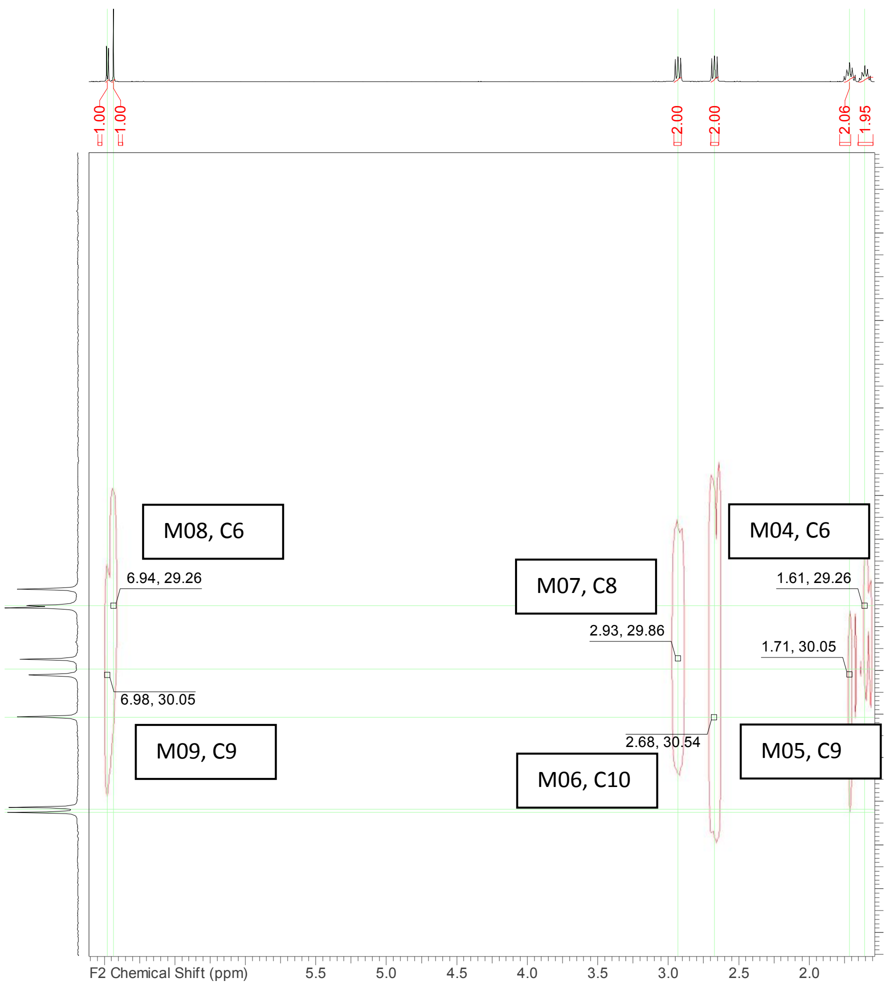

Table S3. Assignation of NMR signals of 11a

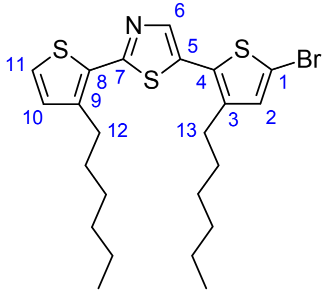

Position	^{1}H chemical shift (ppm)	^{13}C chemical shift (ppm)	Connection observed in HMBC
1	-	111.6	2
2	6.94 (s, 1H)	132.6	1, 3, 4, and 13
3	-	127.7	2,6, and 13
4	-	141.9	2, 6, and 13
5	-	128.9	6
6	7.72(s, 1H)	140.7	3, 4, and 5
7	-	131.4	10, 11, and 12
8	-	160.8	10, 11, and 12
9	-	142.5	10, 11, and 12
10	6.99 (J = 5.1 Hz, d, 1H)	130.5	7, 8, 9, 11, and 12
11	7.32 (J = 5.1 Hz, d, 1H)	126.7	7, 8, 9, and 10
12	2.93 (J = 7.8 Hz, t, 2H)	30.1	7, 8, 9, and 10
13	2.67 (J = 7.7 Hz, t, 2H)	29.3	2, 3, and 4

Figure S28. ^{1}H **NMR spectrum of 11b in** $CDCl_3$

RS43di.001.001.1r.esp

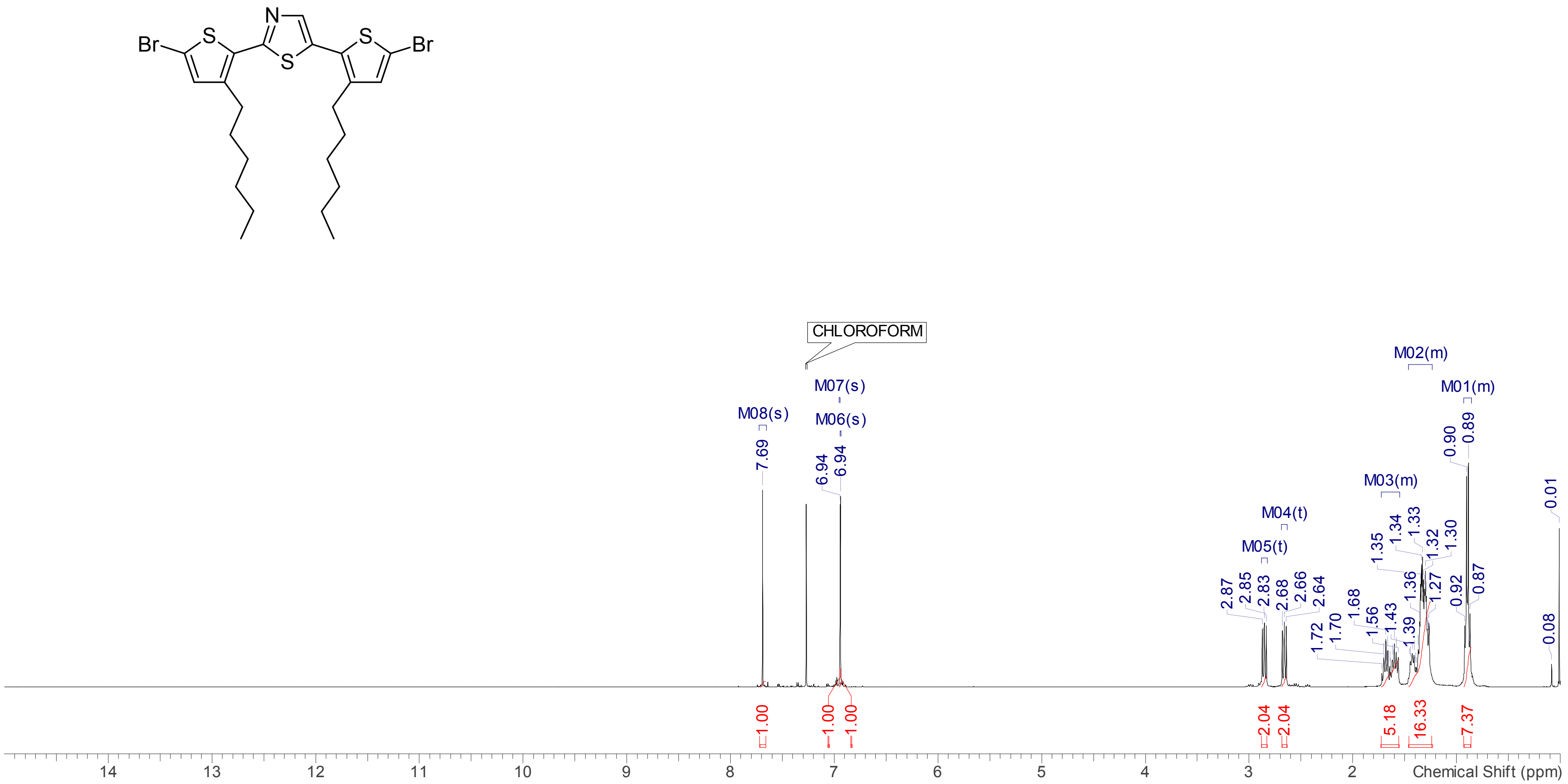

Figure S29. Local zoom of assigned ^1H NMR spectrum of 11b

RS43di.001.001.1r.esp

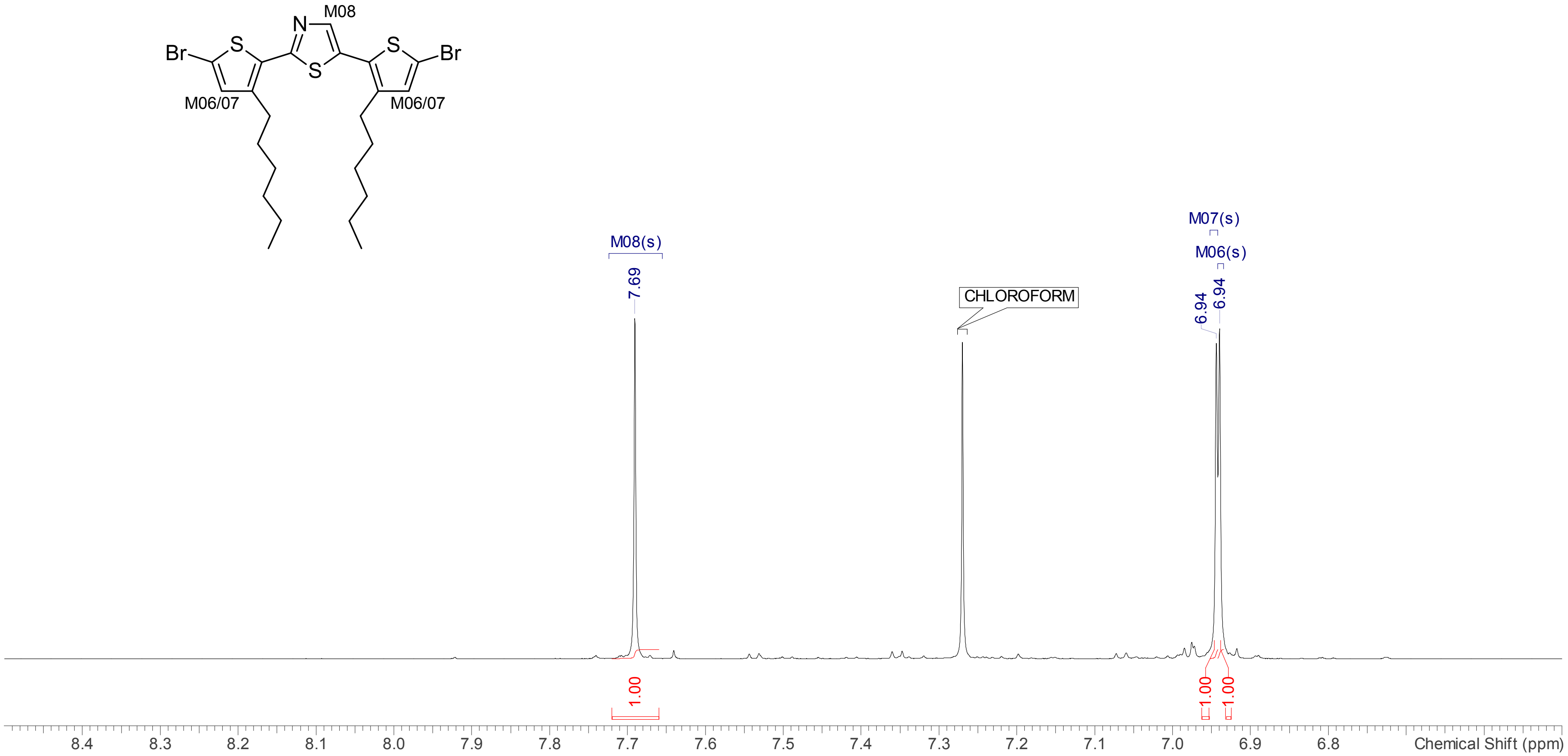

Figure S30. ^{1}H NMR spectrum of 12 in $CDCl_3$

J610.003.001.1r.esp

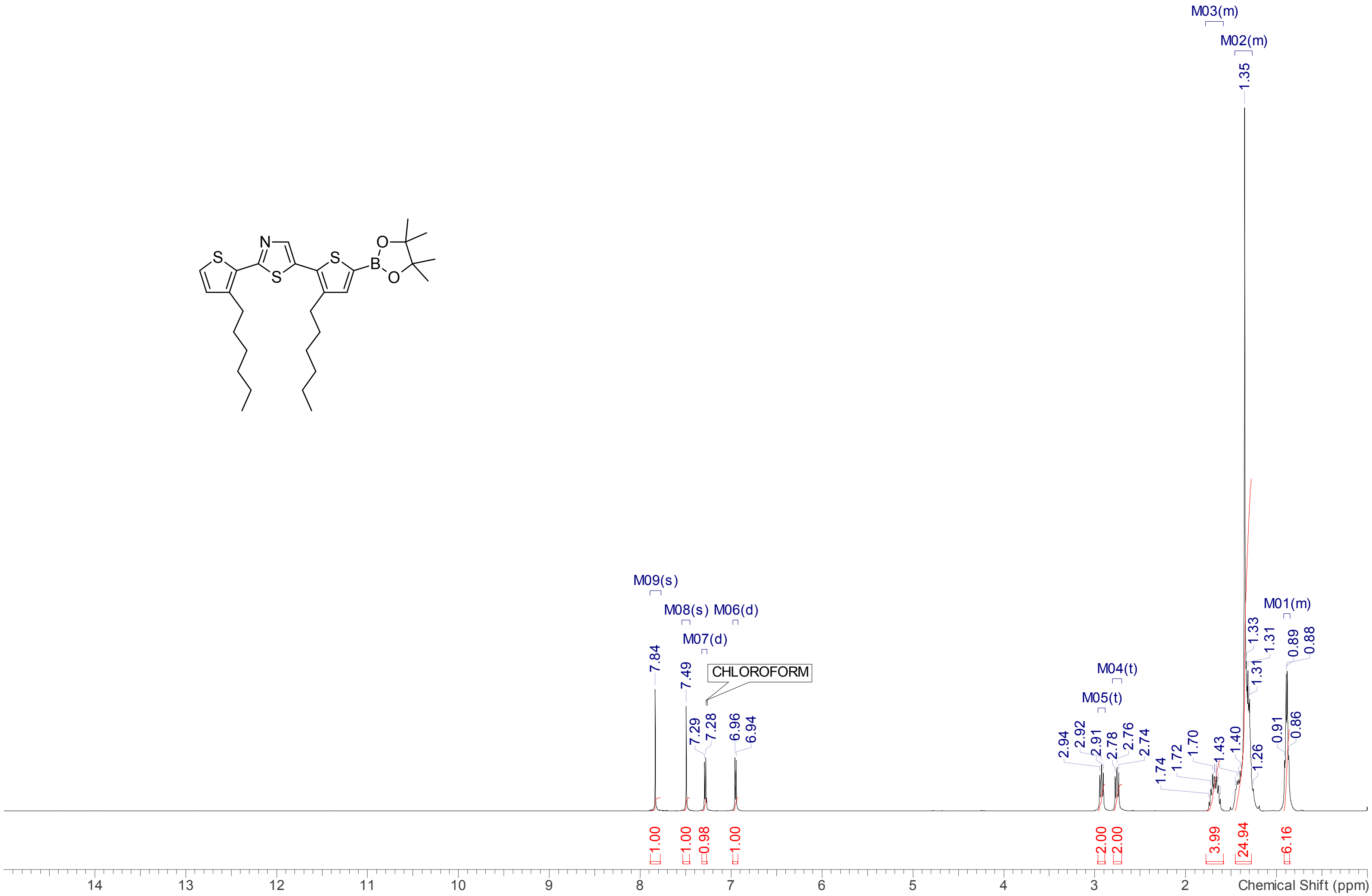

Figure S31. Local zoom of assigned ^{1}H NMR spectrum of 12

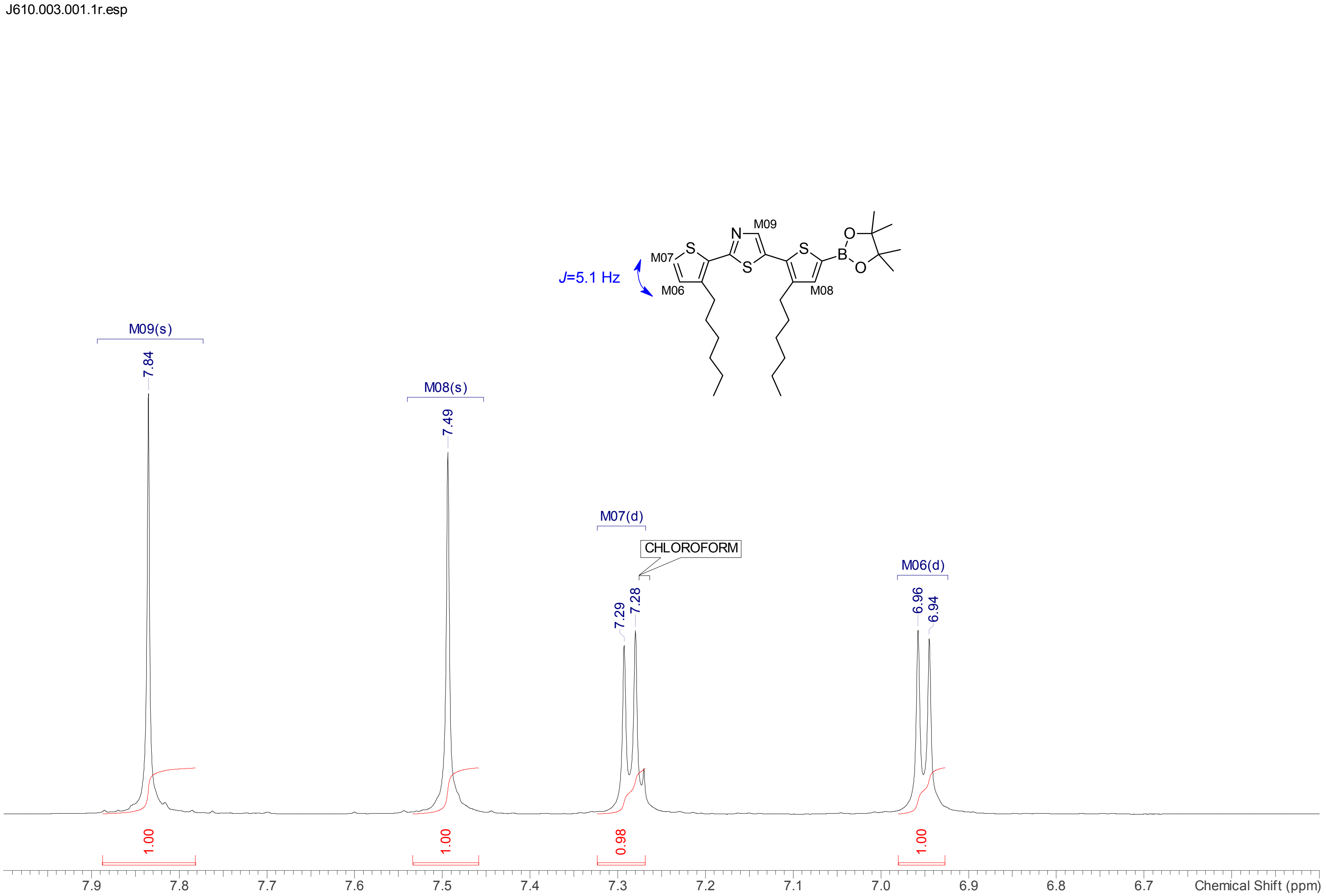

Figure S32. ^{13}C NMR spectrum of 12 in $CDCl_3$

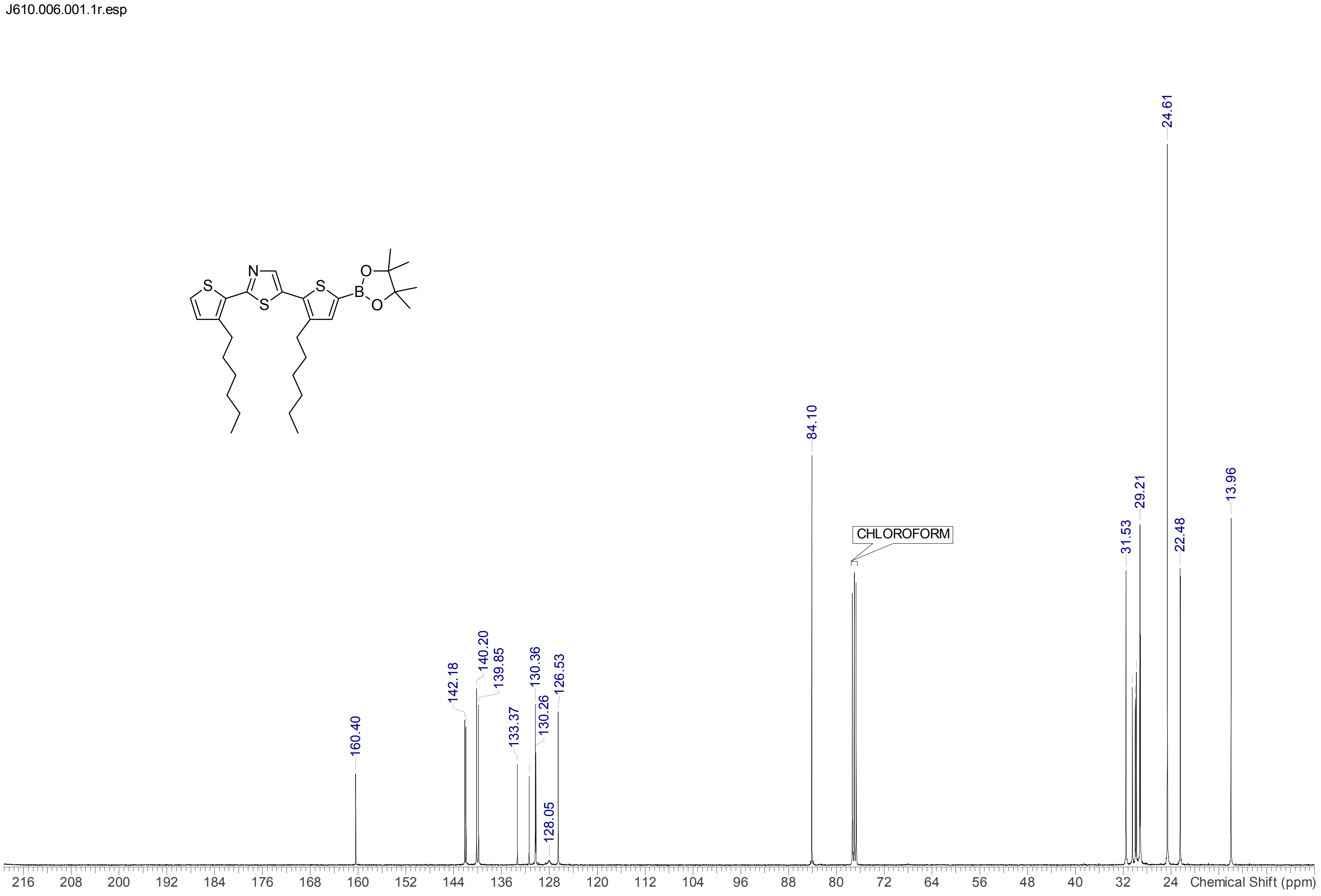

Table S4. Peak list of ^{13}C NMR spectrum of 12 in $CDCl_3$

No.	(ppm)	(Hz)	Height
1	13.96	1404.5	0.4809
2	22.45	2259.2	0.4003
3	22.48	2262.1	0.4115
4	24.61	2476.3	1.0000
5	29.13	2930.4	0.3196
6	29.21	2939.2	0.4720
7	29.79	2997.1	0.2675
8	29.97	3015.5	0.2313
9	30.47	3065.4	0.2463
10	31.51	3170.3	0.3953
11	31.53	3172.5	0.4087
12	84.10	8461.7	0.5679
13	126.53	12730.5	0.2125
14	128.05	12883.1	0.0070
15	130.26	13106.1	0.1557
16	130.36	13115.6	0.2231
17	131.39	13219.8	0.1230
18	133.37	13418.6	0.1396
19	139.85	14070.8	0.2214
20	140.20	14106.0	0.2449
21	141.96	14282.8	0.1917
22	142.18	14304.8	0.2009
23	160.40	16138.8	0.1265

Figure S33. ^{1}H NMR spectrum of 13 in $CDCl_3$

TP33.001.001.1r.esp

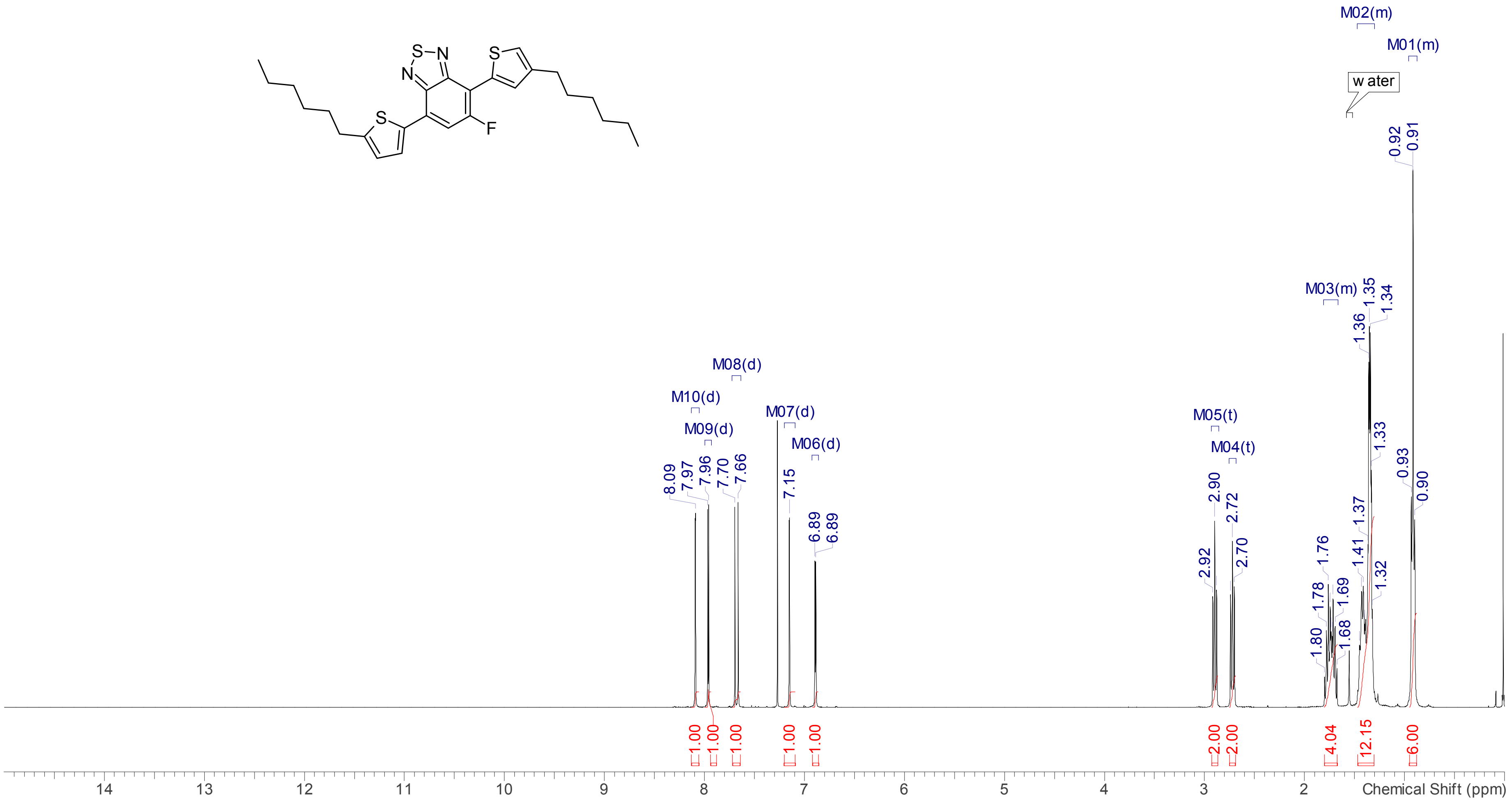

Figure S34. Local zoom of assigned ¹H NMR spectrum of 13

TP33.001.001.1r.esp

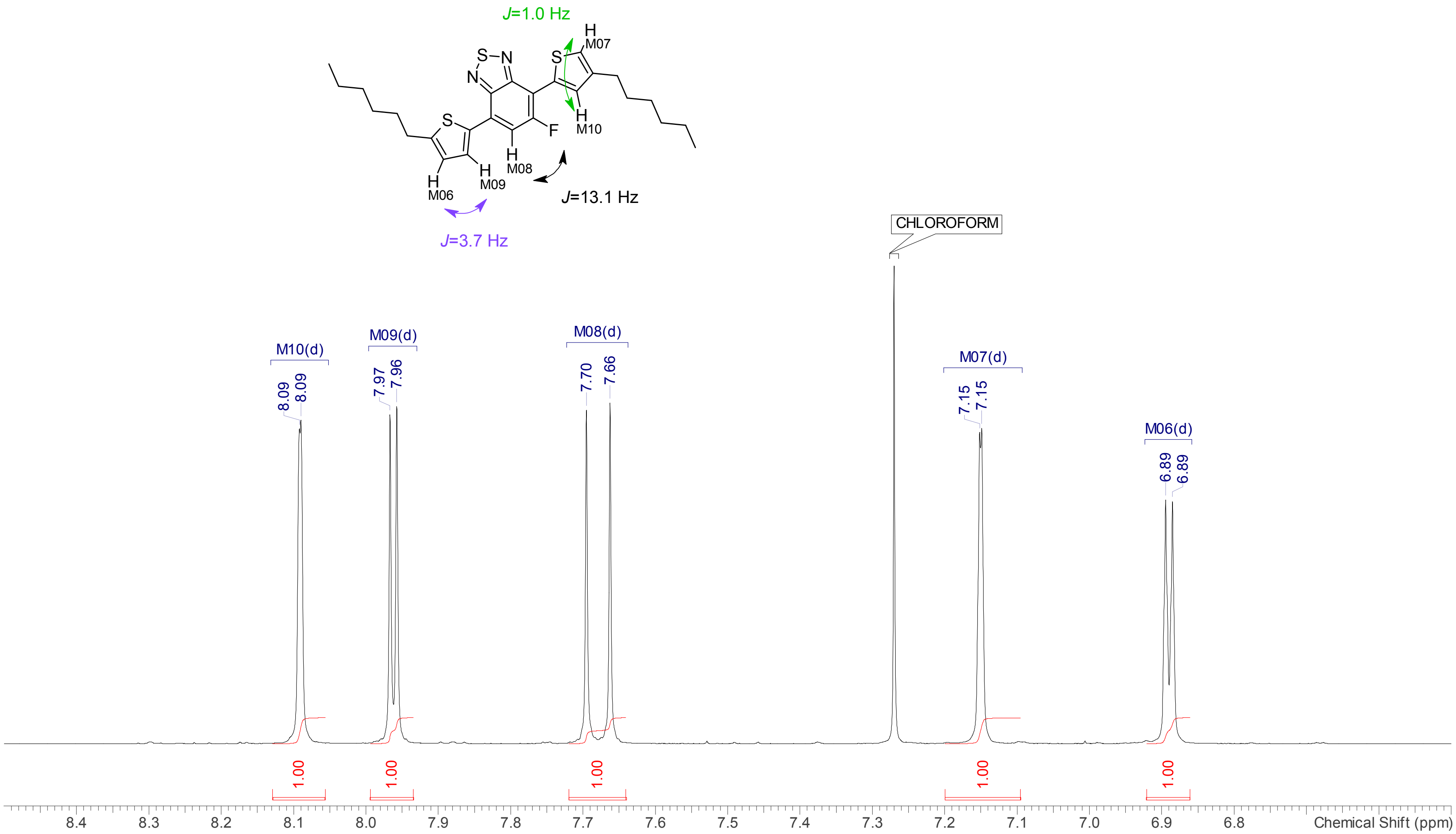

Figure S35. ¹H NMR spectrum of 14 in $CDCl_3$

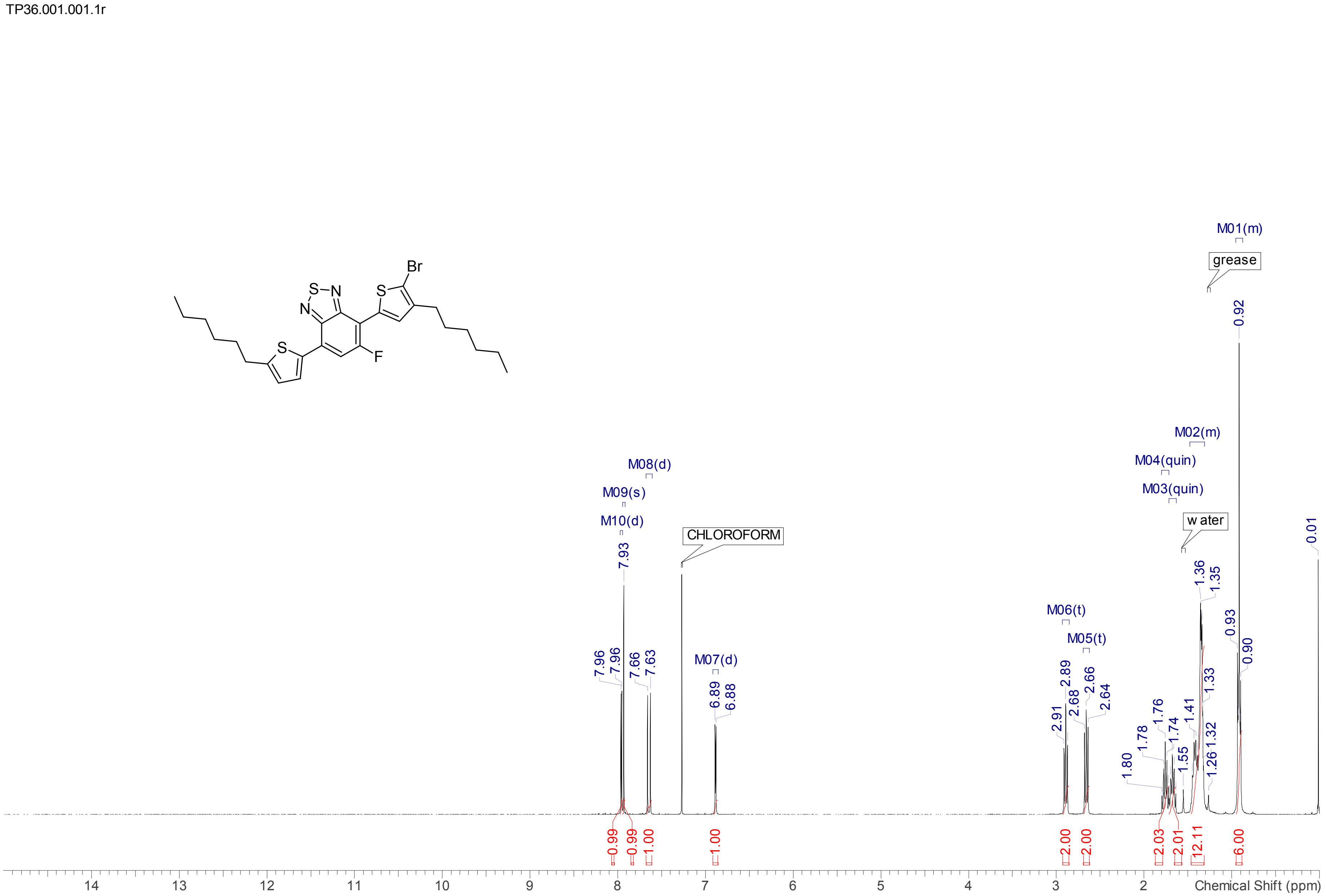

Figure S36. Local zoom of assigned ^{1}H NMR spectrum of 14

TP36.001.001.1r

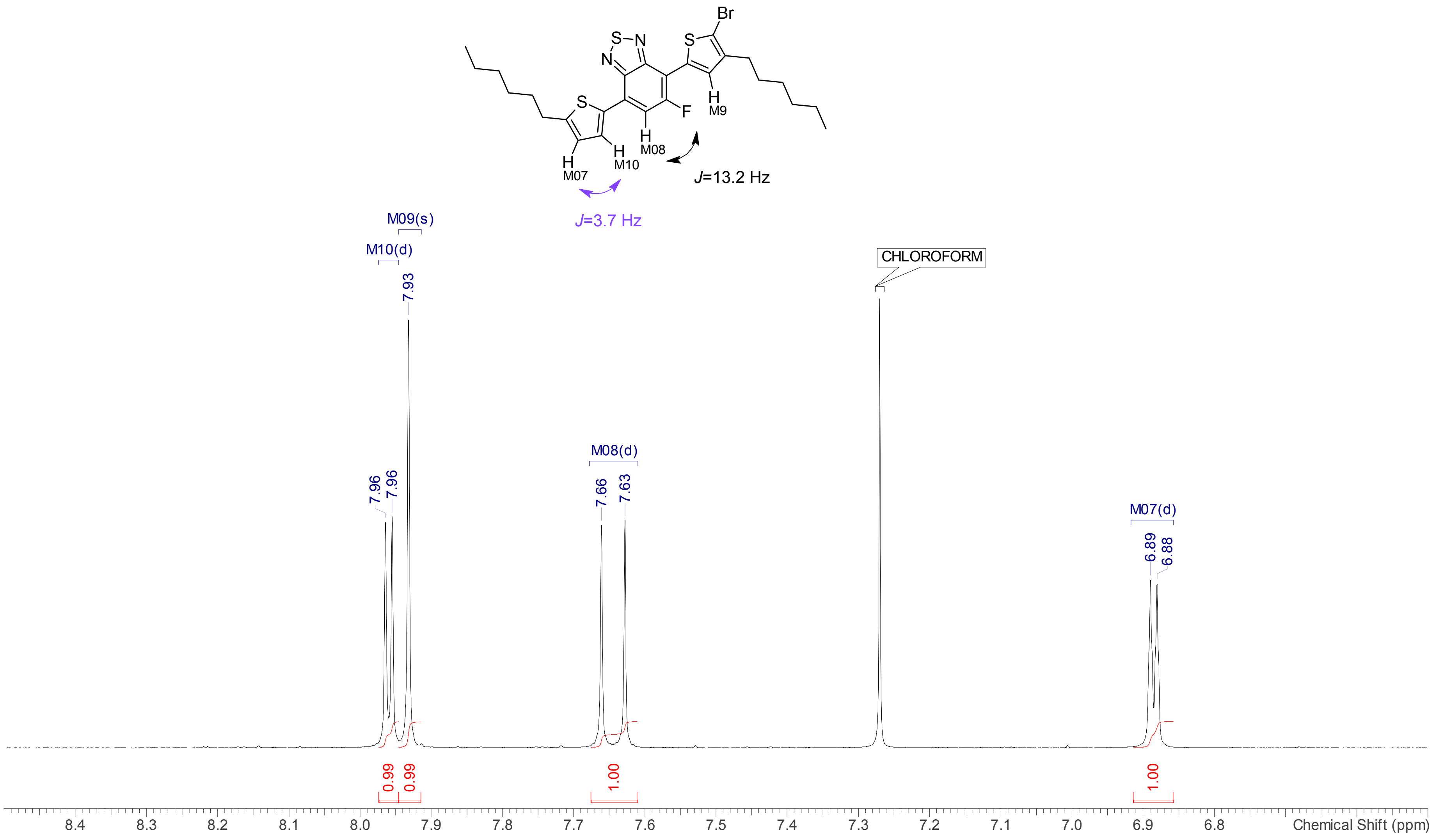

Figure S37. ^{1}H **NMR spectrum of 15 in** $CDCl_3$

TP37.001.001.1r

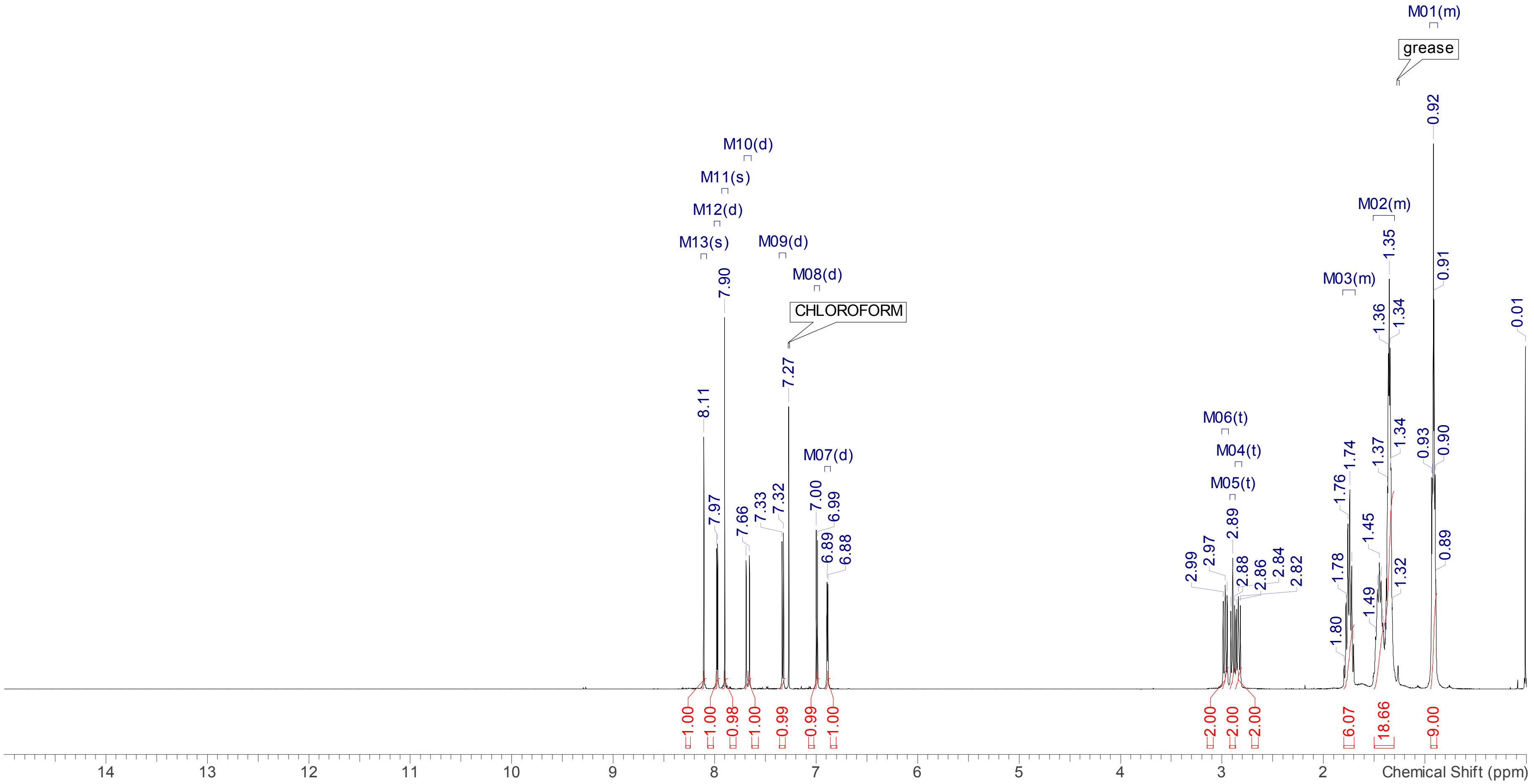

Figure S38. Local zoom of assigned ^{1}H NMR spectrum of 15

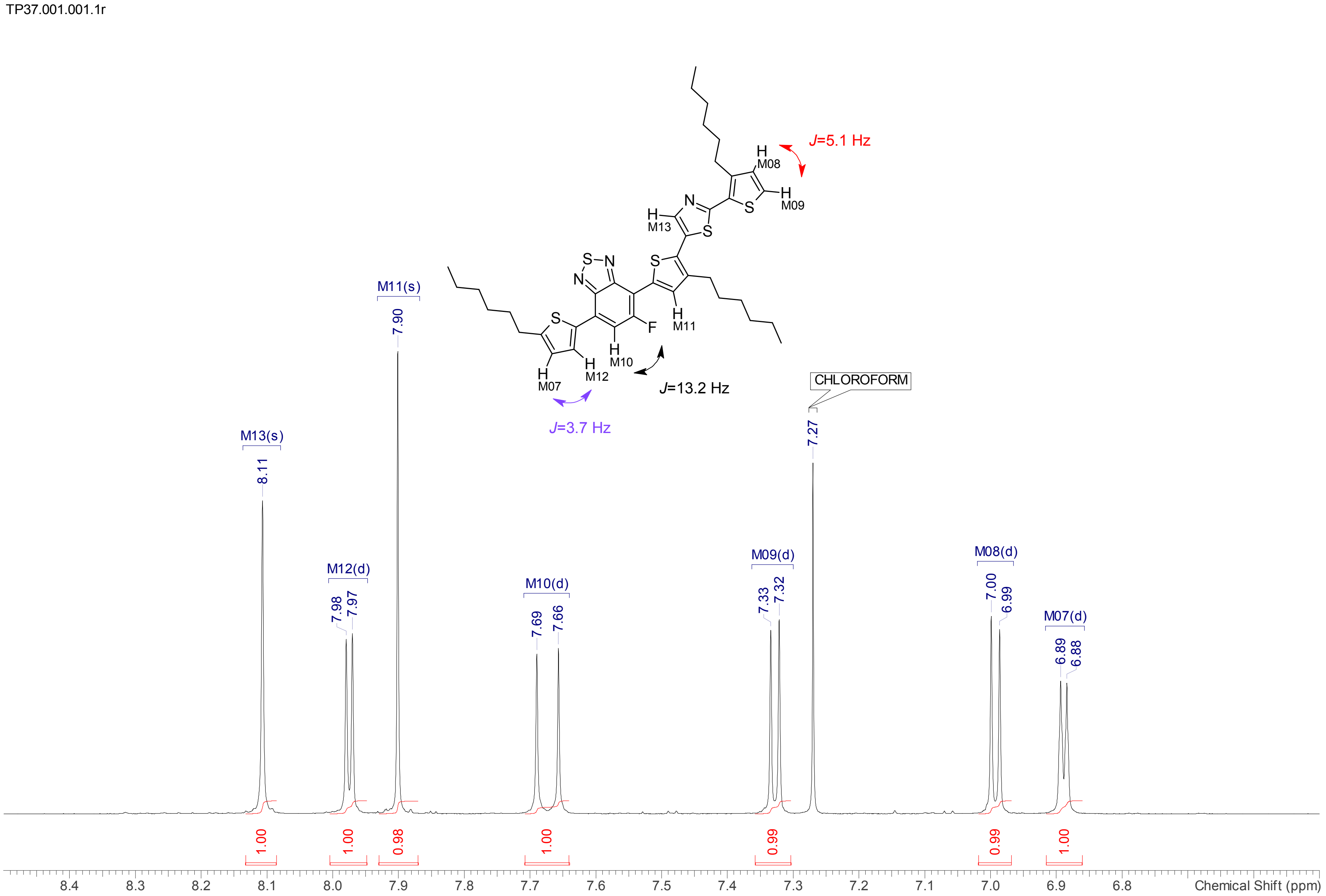

Figure S39. ^{1}H **NMR spectrum of 16 in** $CDCl_3$

TP30.001.001.1r.esp

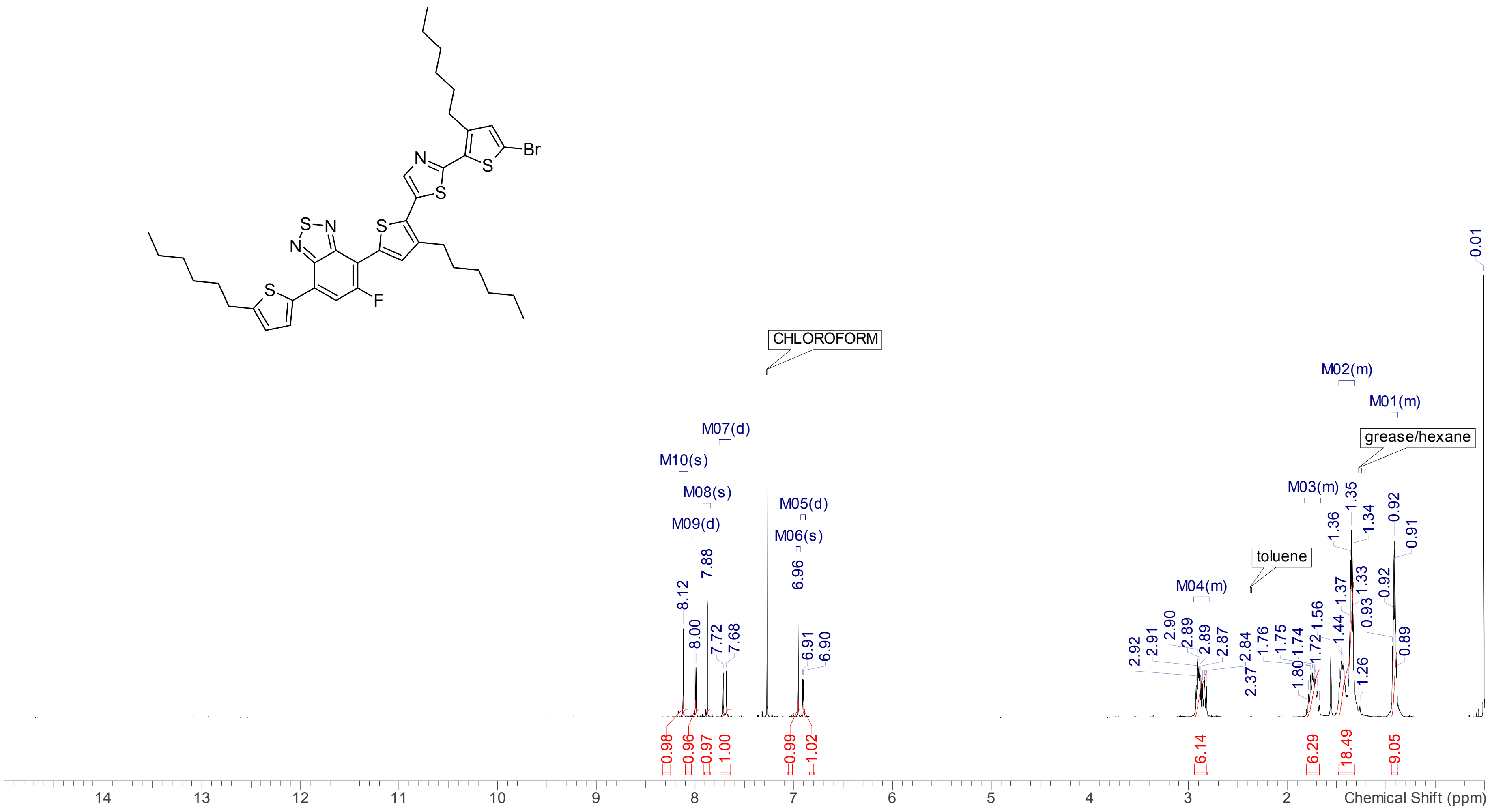

Figure S40. Local zoom of assigned ^{1}H NMR spectrum of 16

TP30.001.001.1r.esp

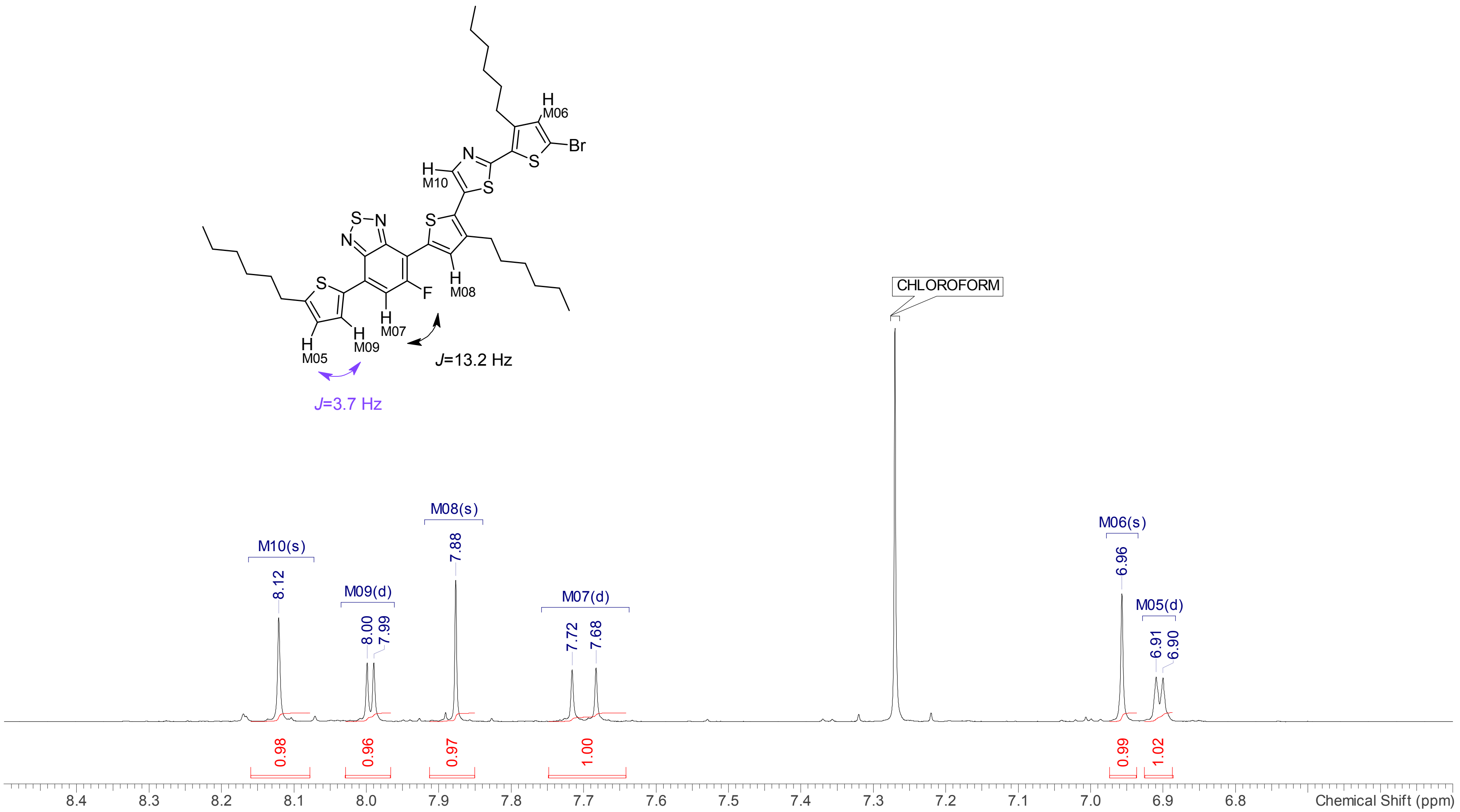

Figure S41. ^{1}H NMR spectrum of compound DTS(Th_2FBTTh)$_2$ in $CDCl_3$

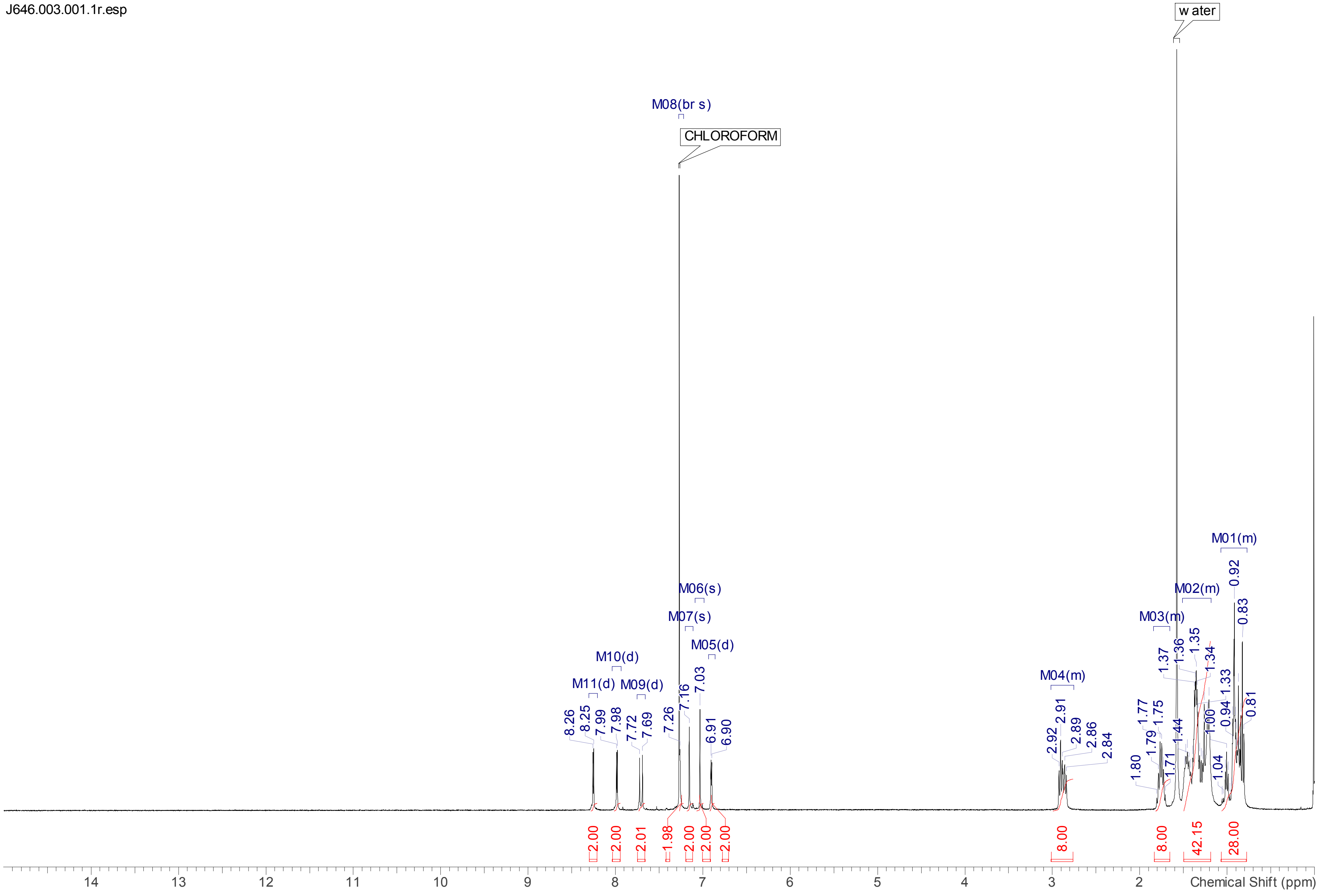

Figure S42. Local zoom of assigned ^{1}H NMR spectrum of compound DTS(Th_2FBTTh)$_2$ in $CDCl_3$

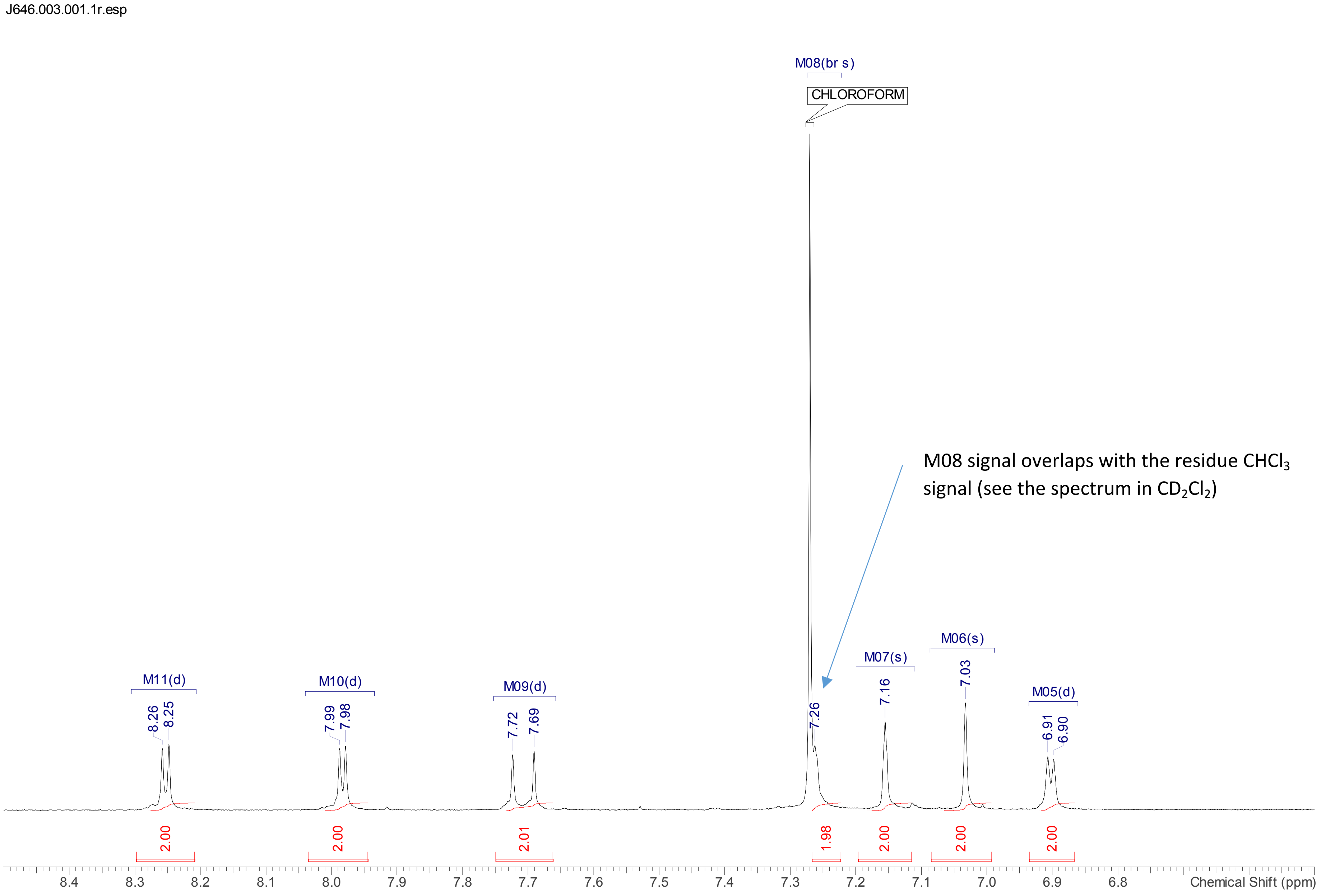

Figure S43. ^{1}H **NMR spectrum of compound** $DTS(Th_2FBTTh)_2$ **in** CD_2Cl_2

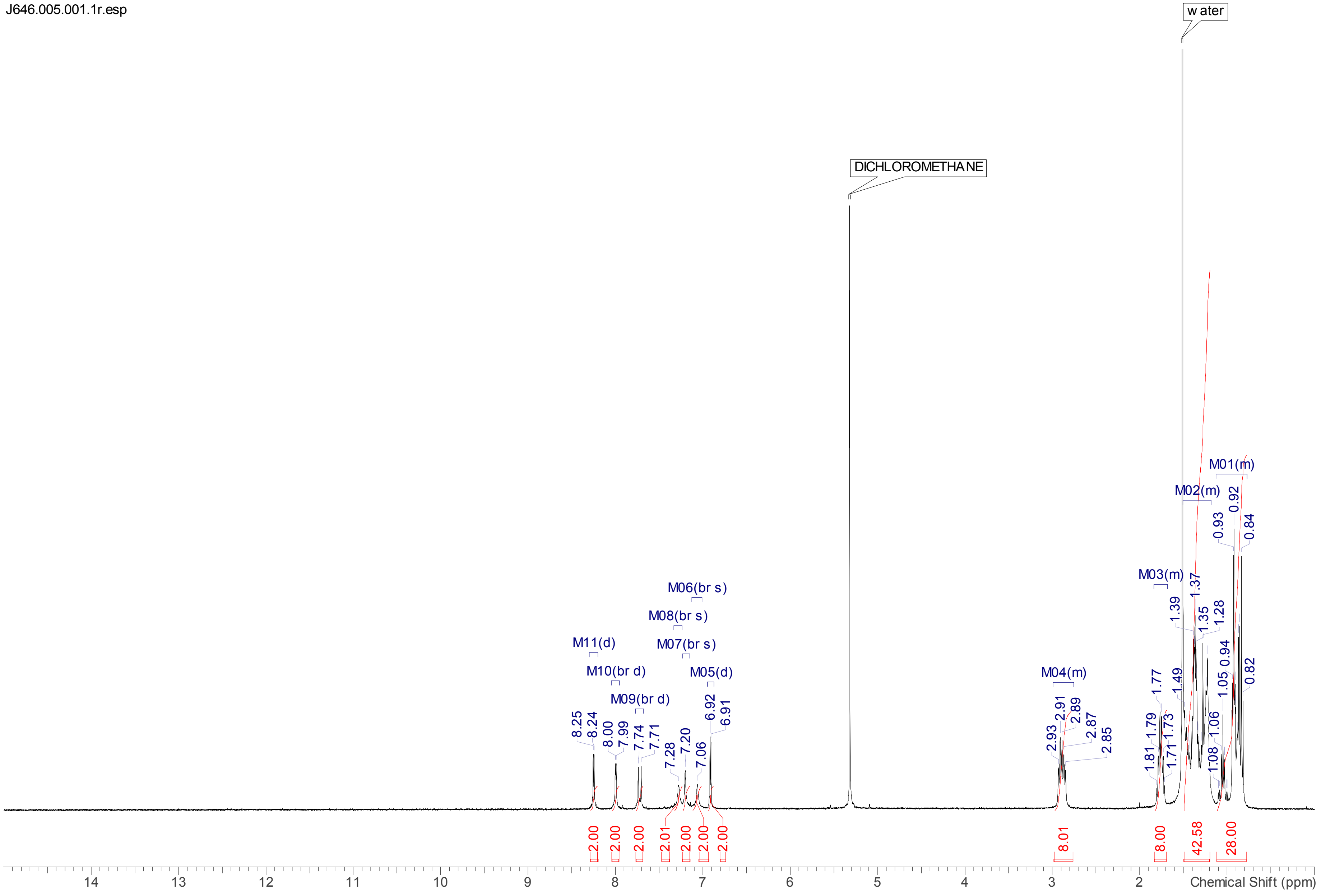

Figure S44. Local zoom of assigned ^1H NMR spectrum of compound DTS(Th_2FBTTh)$_2$ in CD_2Cl_2

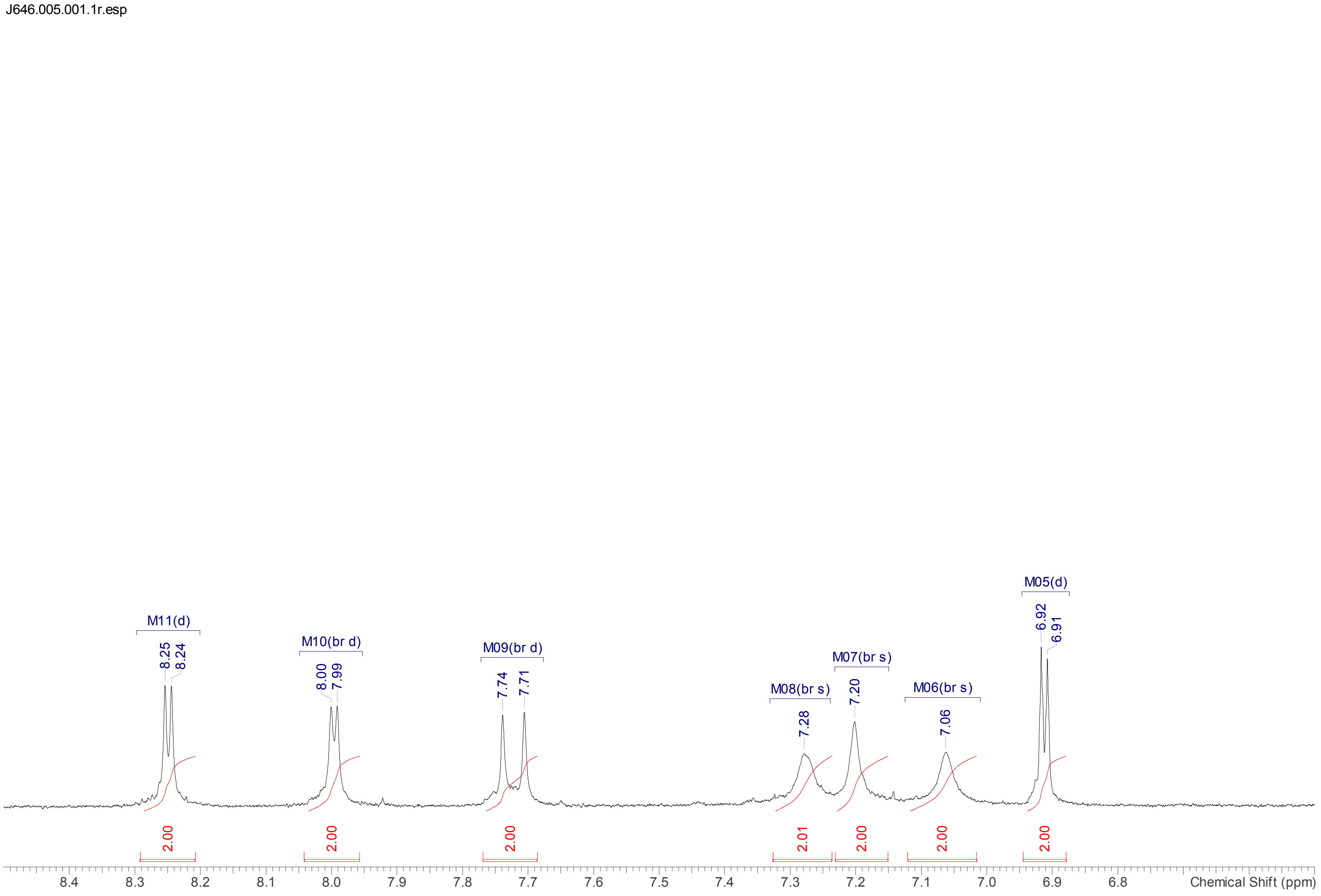

Figure S45. ^{1}H NMR spectrum of compound DTS(ThFBTTh)$_2$ in CDCl$_3$

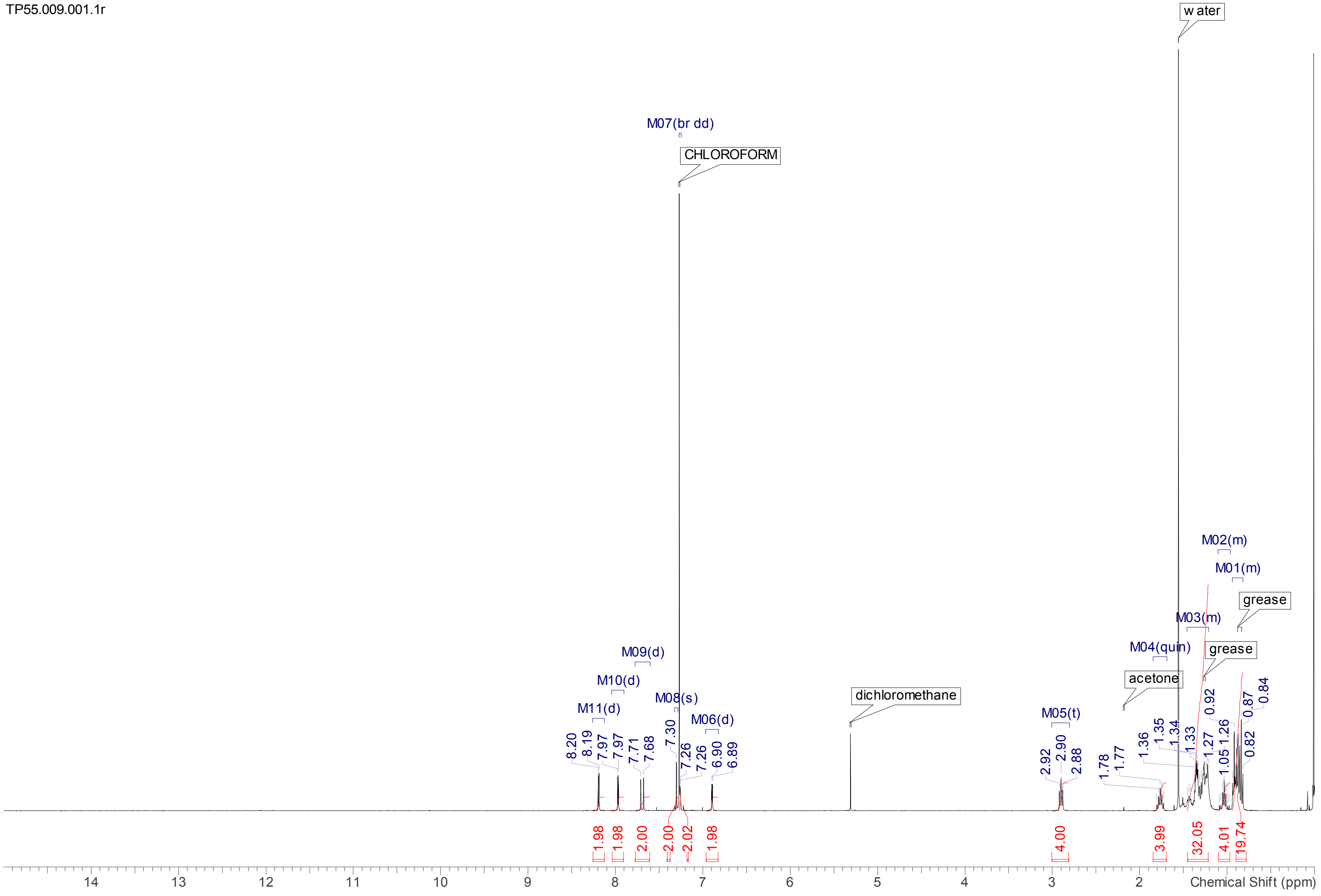

Figure S46. Local zoom of assigned ^{1}H NMR spectrum of compound DTS(ThFBTTh)$_2$ in $CDCl_3$

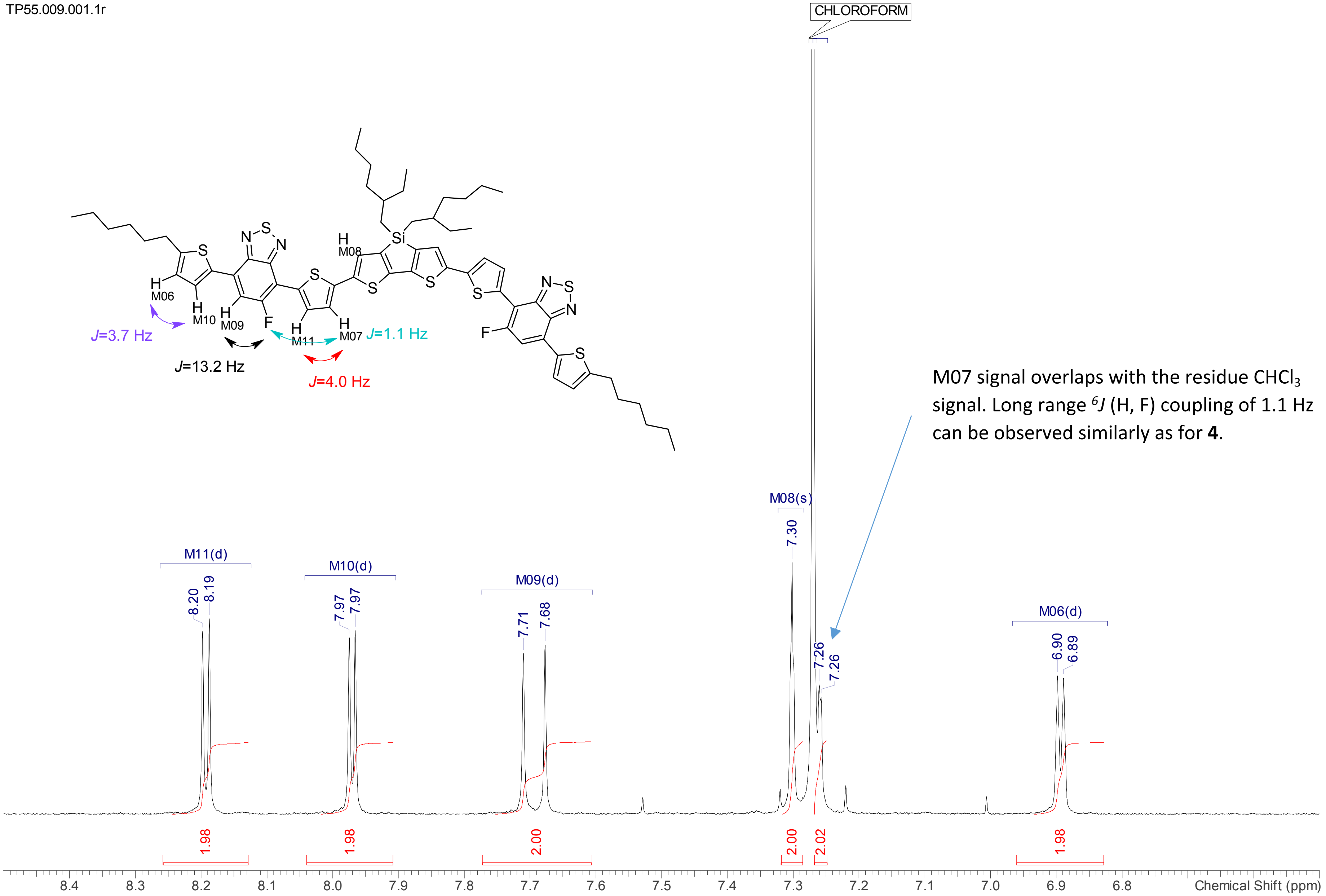

M07 signal overlaps with the residue $CHCl_3$ signal. Long range ^{6}J (H, F) coupling of 1.1 Hz can be observed similarly as for **4**.

Figure S47. Differential scanning calorimetry curve of DTS(Th_2FBTTh)$_2$

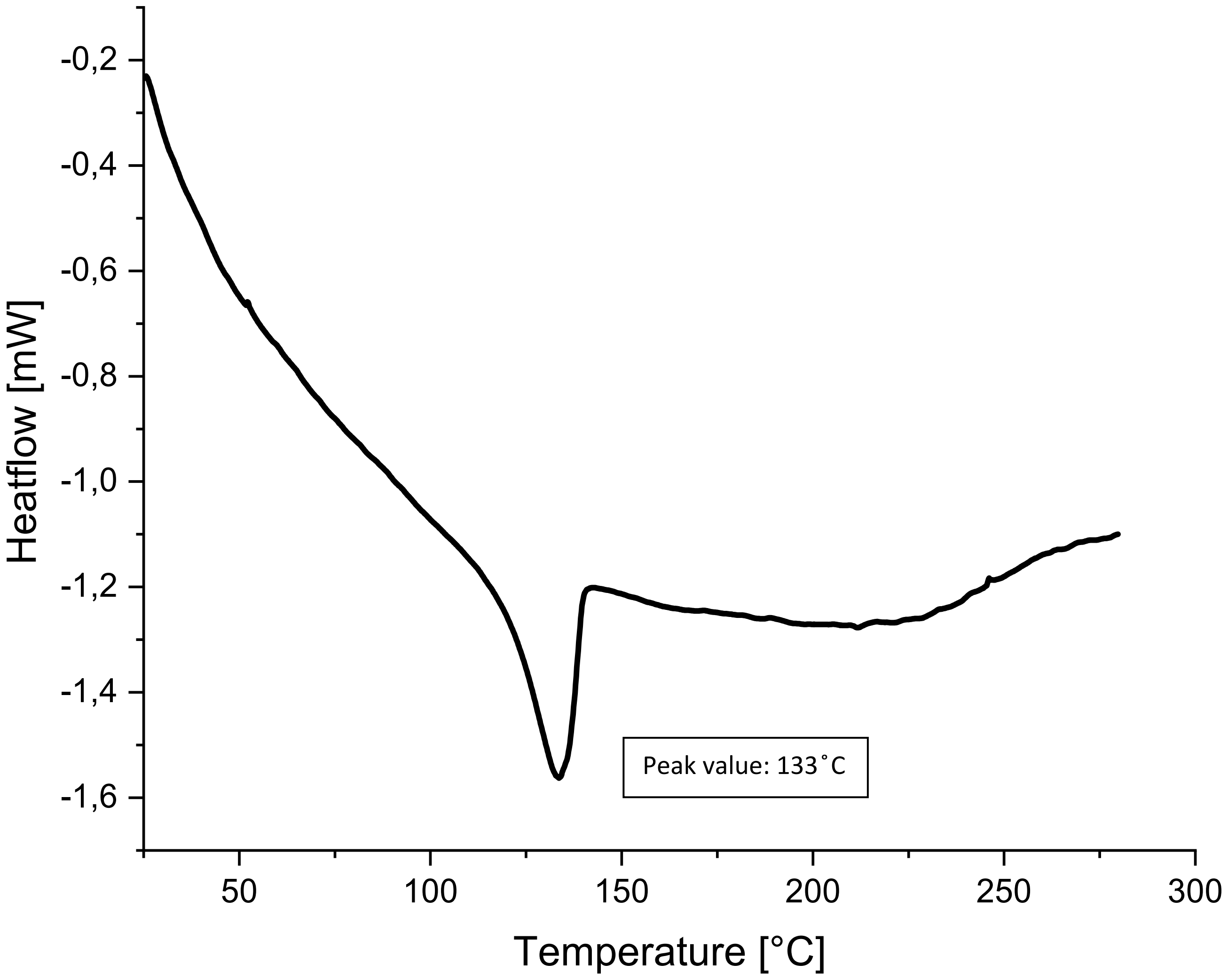

Figure S48. Differential scanning calorimetry curve of DTS(ThFBTTh)$_2$

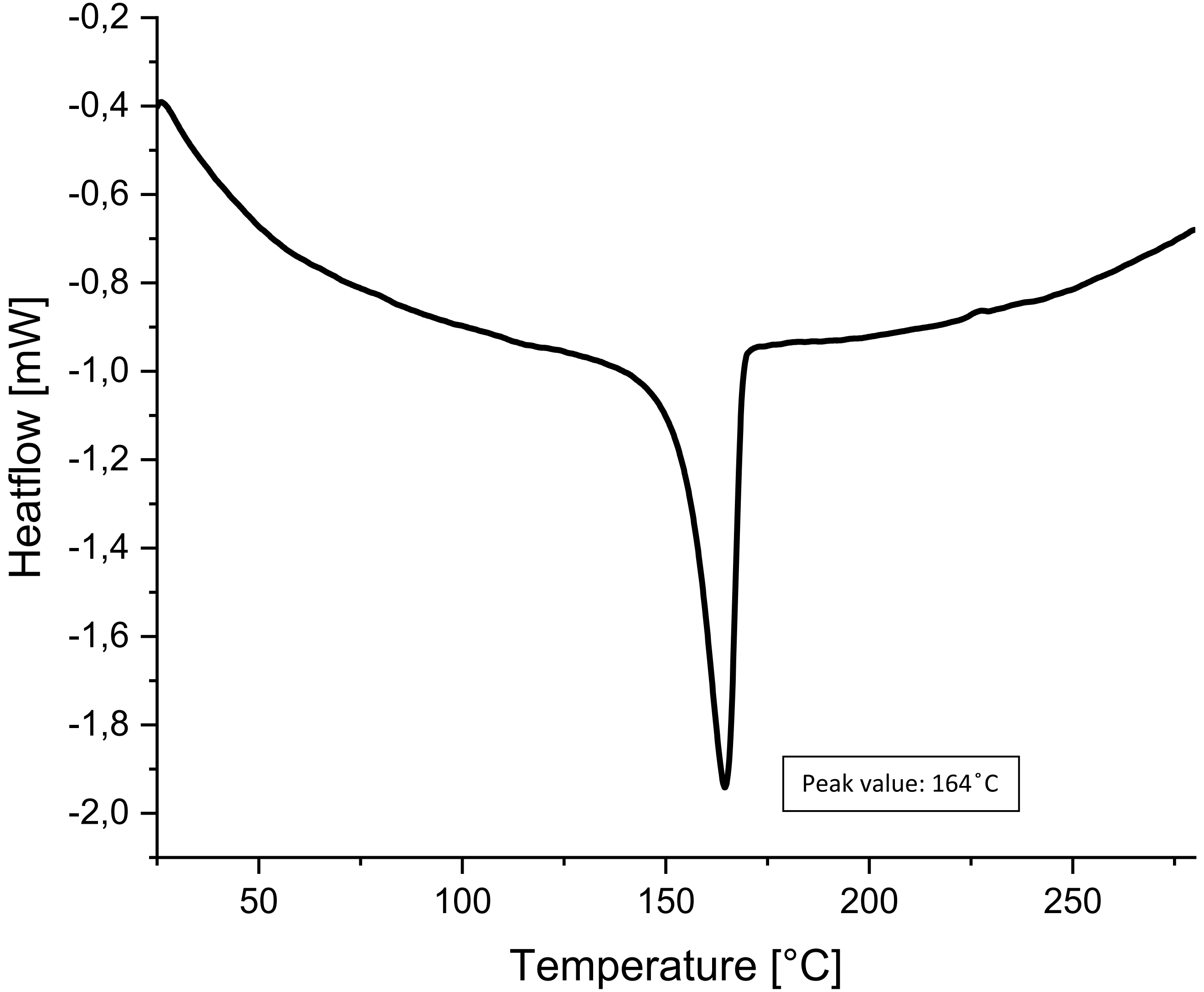

Supplement: Supplementary file 1 — ao2c02195_si_001.pdf [file ao2c02195_si_001.pdf]
